# State-of-the-Art Organic- and Inorganic-Based Hollow Fiber Membranes in Liquid and Gas Applications: Looking Back and Beyond

**DOI:** 10.3390/membranes12050539

**Published:** 2022-05-22

**Authors:** Hui Shen Lau, Siew Kei Lau, Leong Sing Soh, Seang Uyin Hong, Xie Yuen Gok, Shouliang Yi, Wai Fen Yong

**Affiliations:** 1 School of Energy and Chemical Engineering, Xiamen University Malaysia, Sepang 43900, Selangor, Malaysia; mce2009001@xmu.edu.my (H.S.L.); mce2104002@xmu.edu.my (S.K.L.); mce2109006@xmu.edu.my (L.S.S.); mce2109001@xmu.edu.my (S.U.H.); mce2104001@xmu.edu.my (X.Y.G.); 2 U.S. Department of Energy, National Energy Technology Laboratory, 626 Cochrans Mill Rd, Pittsburgh, PA 15236, USA; shouliang.yi@hotmail.com; 3 College of Chemistry and Chemical Engineering, Xiamen University, Xiamen 361005, China

**Keywords:** hollow fiber membranes, organic polymer, inorganic materials, gas separation, liquid separation

## Abstract

The aggravation of environmental problems such as water scarcity and air pollution has called upon the need for a sustainable solution globally. Membrane technology, owing to its simplicity, sustainability, and cost-effectiveness, has emerged as one of the favorable technologies for water and air purification. Among all of the membrane configurations, hollow fiber membranes hold promise due to their outstanding packing density and ease of module assembly. Herein, this review systematically outlines the fundamentals of hollow fiber membranes, which comprise the structural analyses and phase inversion mechanism. Furthermore, illustrations of the latest advances in the fabrication of organic, inorganic, and composite hollow fiber membranes are presented. Key findings on the utilization of hollow fiber membranes in microfiltration (MF), nanofiltration (NF), reverse osmosis (RO), forward osmosis (FO), pervaporation, gas and vapor separation, membrane distillation, and membrane contactor are also reported. Moreover, the applications in nuclear waste treatment and biomedical fields such as hemodialysis and drug delivery are emphasized. Subsequently, the emerging R&D areas, precisely on green fabrication and modification techniques as well as sustainable materials for hollow fiber membranes, are highlighted. Last but not least, this review offers invigorating perspectives on the future directions for the design of next-generation hollow fiber membranes for various applications. As such, the comprehensive and critical insights gained in this review are anticipated to provide a new research doorway to stimulate the future development and optimization of hollow fiber membranes.

## 1. Introduction

Global water and air pollution are on the rising trend due to the rapid growth of industry sectors. Industrial discharges and effluents account for approximately 70% of total environmental pollution [1]. With increasing environmental awareness and energy conservation, treatment of wastewater and exhaust gases from the industrial process is necessary [2]. Conventional gas separation technologies such as cryogenic distillation, pressure swing adsorption and activated sludge systems have been established to purify gases and wastewater, respectively [3,4,5]. These conventional methods are energy-intensive as they account for 10–15% of the world’s energy consumption [6]. Therefore, developing effective and clean technology is important to enhance the efficiency of energy production and energy storage for power generation. Membrane separation has emerged as one of the promising technologies for water and air purification because of its low capital cost, simple operation, small footprint, and low energy consumption [7].

Selecting a suitable membrane module is the key to its application. Tubular, capillary fiber, plate-and-frame and spiral wounds are the main configurations used in industrial applications. The plate-and-frame module consists of flat sheet membranes which are held together by frame-like supports [8]. It has a very low packing density with a typical specific surface area in the range of 100–400 m^2^/m^3^ [9]. Meanwhile, spiral wounds, which are made from layers of flat sheet membranes, have a better packing density of 300–1000 m^2^/m^3^. However, this module relies on feed spacers to promote turbulence within the feed channels to reduce the pressure drops in the system [10]. Notably, the capillary fiber module comprises a large number of hollow fibers which are suitable for various separations [11]. This module offers an excellent packing density of up to 9000 m^2^/m^3^, owing to the high surface-to-volume ratio of the hollow fiber. Not only that, the self-mechanical support property of hollow fiber membrane with high flexibility provides the ease of module fabrication and assembly [12].

**Figure 1:**
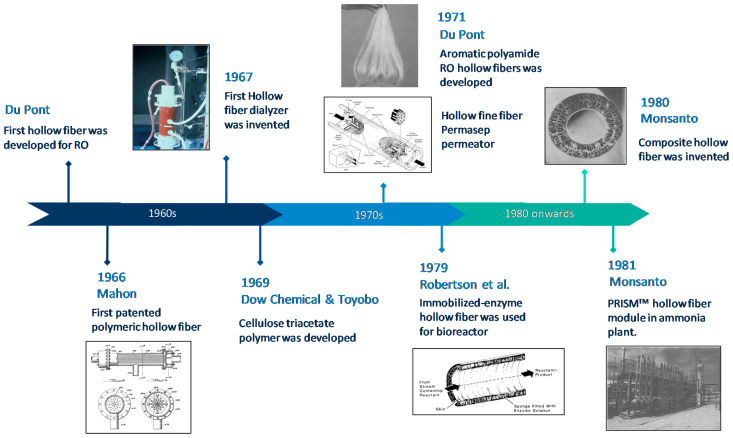
Milestone of hollow fiber membranes development.

Figure 1 illustrates the milestone development of the hollow fiber membranes. The history of hollow fiber membranes was traced back to the 1960s, when the first hollow fiber membrane was developed for the reverse osmosis (RO) process [13]. The first patent on polymeric hollow fiber membrane was known from Mahon [14]. In late 1969, Du Pont commercialized its aramid-based hollow fiber device for water desalination [15]. Then, it was closely followed by Dow Chemical Co. and Toyobo Co. Ltd. (Japan), who developed a cellulose triacetate polymer [16,17]. In the 1970s, hollow fiber membranes had been investigated for various applications like gas separation, water treatment and the medical field. In 1971, the Monsanto PRISM^TM^ system attained momentous breakthroughs in gas separation with its dual-layer polysulfone hollow fiber, which consisted of a relatively dense skin on the exterior surface [15]. In the following year, more patents were identified with the incorporation of thin, non-selective and highly permeable film on the fibers’ surface [18,19,20]. In 1979, for the first time, Waterland et al. developed an anti-fouling membrane by embedding enzymes into pores of hollow fiber membranes [21]. From the 1980s to the 1990s, hollow fiber membrane has been slowly commercialized in biofuel, chemical, medical, petroleum and other fields [22]. For instance, PRISM^TM^ hollow fiber module was used in an ammonia plant for 600 tons/day production.

**Figure 2:**
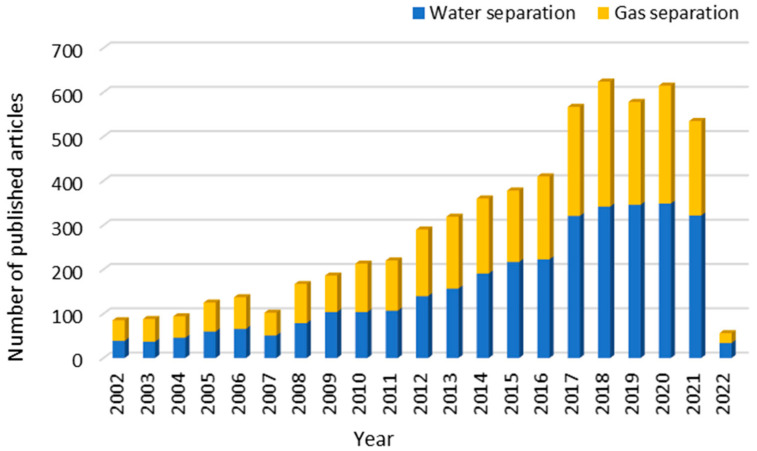
A number of publications in Scopus with the terms “hollow fiber membrane for water separation” and “hollow fiber membrane for gas separation” (accessed on 22 March 2022).

A number of articles on hollow fiber membranes have been published from the year 2000 onward. Figure 2 shows the number of papers established during the past 20 years, where an increasing number of hollow fiber membrane papers has been reported from academic and industrial communities associated with water quality, energy, environment and health sciences [23]. In water separation, the applications of hollow fiber membranes include microfiltration (MF), ultrafiltration (UF), nanofiltration (NF), RO, forward osmosis (FO), membrane contactors, pervaporation and membrane distillation. Non-solvent induced phase separation (NIPS), thermally induced phase separation (TIPS) and diffusion-induced phase separation (DIPS) are the most common fabrication methods for porous hollow fiber support [24,25,26]. A composite membrane is formed by coating the dense and selective layer onto the porous support [27]. It can be fabricated through dual-layer spinning [28,29], interfacial polymerization [30], dip-coating [31] and plasma polymerization [32]. Mixed matrix hollow fiber membranes are another type of membrane that has been widely applied for gas purification, owing to the unique features given by combining porous polymer and inorganic fillers [23]. Incorporating nanostructure fillers into hollow fiber membranes provides excellent gas separation performance. Hollow fiber membranes have been widely used in hydrogen purification, natural gas sweetening, carbon capture and storage, and landfill gas upgrading [33,34,35,36,37,38]. Recently, green and sustainable approaches for hollow fiber preparation techniques have been developed to minimize chemical or solvent and energy usage [39]. The methods being reported are melt/solution integrated homogeneous-reinforcement, melt spinning-stretching (MS-S) interfacial phase separation and electrospinning methods [40,41,42]. These methods hold great promise for an environment-friendly fabrication.

However, hollow fiber membranes exhibited some limitations in both gas and liquid separation. For gas separation, most polymeric membranes face a trade-off between permeability and selectivity [43]. This phenomenon is significantly related to the non-uniform distribution of free-volume elements, which allows a wide range of gases to diffuse through the membrane. Physical aging and plasticization are challenges encountered during gas separation [44,45]. Physical aging is caused by the tightening of ultramicropores, which results in a rapid decrease in polymer free volume, thus causing poor separation performances [46]. Upon plasticization, which is caused by highly condensable carbon dioxide (CO_2_) and heavy hydrocarbons, the polymer matrix becomes swollen, thus increasing permeability which causes loss in selectivity [47]. Similarly, liquid separation membranes are also confined by the trade-off effect between permeability and solute rejection. Apart from that, the membranes are hindered by membrane fouling, which is the accumulation of particles, colloids, suspensions and microorganisms on or in the membrane [48,49]. Hollow-fiber unit is prone to fouling and plugging by particulate matter due to a relatively low free space between fibers as well as the small inside diameters [13,17]. Consequently, the separation performance and lifespan of the membranes are adversely affected, and additional costs may be imposed for membrane cleaning [50].

To tackle the aforementioned problems, researchers have synthesized hollow fiber membranes from two broad classes of materials, namely organic and inorganic materials. Various modification strategies, namely interfacial polymerization, dip-coating, etc., have been developed and optimized throughout the years. In the past decade, there have been several reviews on hollow fiber membranes published from the aspect of a specific application, for instance, gas separation [27,51,52,53], pervaporation [52], NF [54,55], RO [55] and wastewater treatment [56]. In addition, Ahmad et al. investigated the influence of fabrication parameters on the morphology and performance of hollow fiber membranes [57], while Huang et al. summarized sustainable and green fabrication methods of hollow fiber membranes [39]. Other than Dashti and Asghari’s work on ceramic hollow fiber membranes [58], most of the aforementioned hollow fiber membrane reviews only covered polymeric membranes. To the best of our knowledge, only two publications covering both liquid and gas separation applications of hollow fiber membranes were published 10 years ago [11,59].

**Figure 3:**
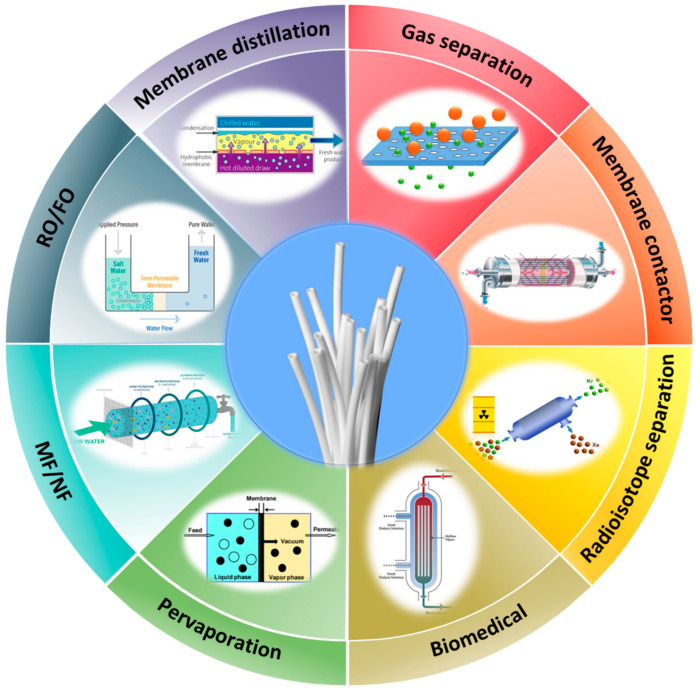
The applications of hollow fiber membranes.

Our previous work reviewed polymeric hollow fiber membranes for gas application, water vapor separation and particulate matter removal [12]. This work will extend not only the types of materials for hollow fiber membranes but also their respective applications. Figure 3 illustrates the main applications of hollow fiber membranes studied in this review. The aims of this review are to: (1) provide updates on the recent developments and fabrication methods of hollow fiber membranes; (2) summarize the state-of-the-art hollow fiber membranes in various fields such as liquid separation, gas and vapor separation, pervaporation, membrane distillation, and membrane contactor and (3) provide insights and recommendations on green technologies to accomplish fruitful advancement of hollow fiber membranes.

## 2. Fundamental Aspects of Hollow Fiber Membranes

### 2.1. Structural Analyses on Hollow Fiber Membrane

A hollow fiber membrane is a tube-like membrane consisting of an ultrathin dense selective layer supported on the outer layer of a highly porous substrate. The substrate acts as a mechanical support, while the dense layer acts as a selective layer. The thickness of a hollow fiber membrane ranges between 0.1–0.4 mm, with an inner diameter of <1 mm [60]. As a promising candidate in industrial practice, hollow fiber membranes offer vast advantages such as self-supporting characteristics, large surface area and high packing density [61,62]. For instance, a hollow fiber module of 20 cm × 1 m is able to purvey a membrane area of ~300 m^2^ with fibers of 100 μm diameter, while a spiral wound module gives only 20–40 m^2^ membrane area with an equivalent dimension [63].

The development of high-performance hollow fiber membranes with ideal structural properties is indispensable. Generally, hollow fiber membranes are prepared following these five steps: (1) formulate the dope solution, (2) extrusion of dope solution through spinneret, (3) coagulation of fiber after passing through a certain air gap via phase inversion, (4) collection, washing and drying of as-spun fibers, and (5) post-treatment (if required). The phase inversion process has been widely applied during the fabrication of hollow fiber membranes owing to its scalability and simplicity for large-scale production [63]. Phase inversion is introduced via several factors, where NIPS and TIPS are the most common ones. Phase separation in NIPS and TIPS is induced by composition changes after the introduction of nonsolvent and temperature changes, respectively. TIPS has been widely used to prepare membranes from semicrystalline polymers [64]. Detailed discussions on these phase separation processes are available in Section 2.2.

There are a number of factors that are intrinsically intertwined which affect the structural morphology and separation performance of the resultant membrane during the fabrication of hollow fiber membranes. These include the (1) selection of spinning techniques, (2) formulation of dope solution, (3) design of spinneret, and (4) proper tuning of spinning parameters [65]. The individual effects of all these factors/parameters require critical experimental analysis. For instance, the critical concentration of a polymer/solvent system can be pre-determined via the correlation of polymer concentration and shear viscosity [11,66]. A dope solution with a low concentration reduces the degree of polymer chain entanglement during the phase inversion process, thus leading to a higher porosity membrane but with low selectivity [67]. Meanwhile, an increase in take-up speed during hollow fiber spinning increases the selectivity due to a reduction in the duration of phase inversion [68,69]. It leads to a greater molecular orientation and chain packing within the fiber due to the gravitational and elongational forces, subsequently reducing the overall diameter and thickness of the as-spun fiber [57]. Despite the vast variables, the hollow fiber membrane is prepared with a single, dual or triple nozzle configuration spinneret. A comprehensive analysis of hollow fiber spinning techniques, spinning parameters, spinning effects, and spinneret design has been reported elsewhere [12].

**Figure 4:**
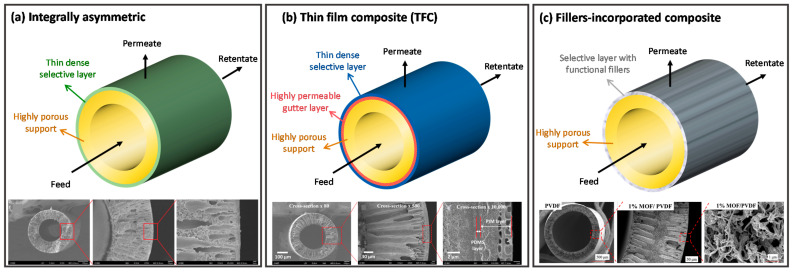
Schematic and cross section morphologies of (**a**) integrally asymmetric, (**b**) TFC, and (**c**) fillers-incorporated composite hollow fibers. Reprinted/adapted with permission from Refs. [66,70,71]. Copyright 2018 and 2021, Royal Society of Chemistry, Elsevier and SpringerLink.

Single-layer hollow fiber membranes such as polymeric-based, perovskite-based, carbon molecular sieve (CMS) and thermally rearranged (TR) hollow fiber membranes are made from the same material. On the other hand, a hollow fiber membrane that is composed of two or more layers with distinctive roles prepared via dual or triple nozzle spinneret, dip coating, interfacial polymerization and deposition is referred to as a composite membrane. Common examples of composite membranes are dual-layer and thin film composite (TFC) hollow fiber membranes. Figure 4 shows the configuration and morphologies of (a) integrally asymmetric and (b) composite hollow fiber membranes.

### 2.2. Phase Inversion Mechanism during Hollow Fiber Formation

Among all the membrane fabrication methods, phase inversion is the major and substantial technique to synthesize most commercial membranes [72]. Compared to the common flat-sheet membranes, the phase inversion process for hollow fiber membranes is distinct from the aspect of dope formulation and coagulant usage [11]. For hollow fiber membranes, the spinning process necessitates a higher polymer dope concentration, and they require an internal coagulant that determines the inner skin structure and an external coagulant that influences the outer skin morphology. In order to understand the essence of membrane formation, this section delineates the fundamentals of phase inversion. Phase inversion is defined as the transformation of a polymer from a liquid to a solid state in a controlled approach [65]. During this process, a thermodynamically stable polymer solution is converted into a polymer-rich phase and a polymer-lean phase to lower the Gibbs free energy of mixing, ΔG_M_ of the system, which is determined by the Flory–Huggins theory. The polymer-rich phase primarily forms the membrane matrix, while the membrane pores are formed by the polymer-lean phase, leading to a phenomenon known as liquid–liquid demixing.

**Figure 5:**
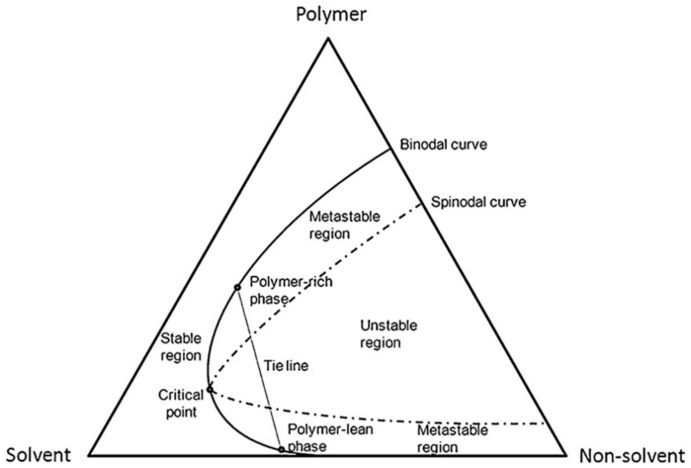
Typical ternary phase diagram of a polymer-solvent-non-solvent system. Reprinted/adapted with permission from Ref. [11]. Copyright 2012, Elsevier.

Generally, the phase inversion process is explained using a ternary phase diagram (Figure 5). The ternary phase diagram was first proposed by Tompa [73] in the 1950s and later applied by Strathmann et al. [74] and Michaels [75]. Located at the corners of the triangle are the pure components in the system (polymer, solvent, and nonsolvent), and any point which lies in the triangle consists of all three components. The crucial elements in this diagram are the binodal and spinodal curves, tie-line, critical point, and metastable region [11,76]. The spinodal curve delimits the unstable region of the miscibility gap, whereas the tie-line connects two points that are in thermodynamic equilibrium on the binodal. The intersection between the binodal and spinodal curves is called the critical point. Moreover, the area between the binodal and the spinodal curve is named the metastable region, where the polymer solution is thermodynamically unstable, but it does not precipitate.

Notably, the two factors that influence the phase transition are the thermodynamic equilibrium of a ternary system (polymer/solvent/nonsolvent) and the kinetic factor, which includes mass transfer between all the components during precipitation [77]. The concept of phase inversion can be applied in various methods, for instance, immersion precipitation, evaporation-induced phase separation, vapor-induced phase separation, and thermally induced phase separation. Each of the methods will be discussed thoroughly in the following sections.

## 3. Types of Hollow Fiber and Their Preparation Route

Membranes can be categorized according to the nature of the material used for their fabrication, characteristics, applications, and reaction mechanisms. Based on the types of material used, membranes are classified into organic (e.g., polymeric and conducting polymer) and inorganic (e.g., metallic, ceramic, etc.) membranes. Organic-based polymeric membranes possess good chemical and mechanical properties. On the contrary, inorganic membranes consisting of oxides, metals, or elementary carbon have better stability in harsh conditions such as high pressure or temperature and are highly selective and permeable for specific molecules.

**Figure 6:**
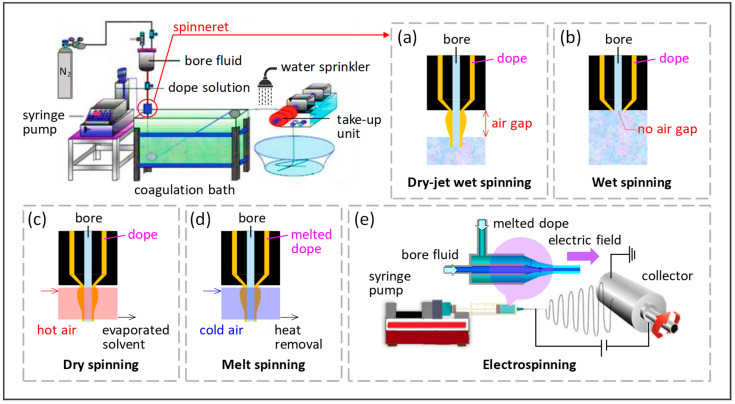
The illustration of different hollow fiber spinning techniques including (**a**) dry-jet wet-spinning, (**b**) wet spinning, (**c**) dry spinning, (**d**) melt spinning, and (**e**) electrospinning.

Figure 6 illustrates the conventional methods to fabricate hollow fibers, including dry spinning, wet spinning, dry-jet-wet-spinning (also known as gel spinning), melt spinning, and electrospinning. Spinneret is constructed in the tube-in-an-orifice structure to produce fibers with desirable dimensions. During spinning, bore fluid and polymer dope are extruded from the spinneret and travel through an air gap before being subjected to a coagulation bath for phase inversion. The presence of an air gap in the dry-jet-wet spinning process allows the orientation and coalescence of polymers on the external surface of fibers prior to gelation in the coagulation bath, while the lumen surface coagulates right after extrusion from the spinneret (Figure 6a). High air gap distance will result in flow instability, thus producing fiber with a non-uniform dimension. On the other hand, no air gap is required for wet spinning (Figure 6b). Spinneret is submerged in the coagulation bath, allowing instant entering of polymer dope after extrusion for immediate precipitation and solidification of fibers. The as-spun fibers are then collected in a collecting drum guided by a take-up unit. Subsequently, the fibers are subjected to solvent exchange before drying and post-treatment [9]. The washing protocol can be found elsewhere [12]. After washing, the fibers are dried and ready for post-treatment or further characterization.

Dry spinning utilizes air or inert gas to evaporate the residual solvents present in the polymer dope, allowing the formation of solid fiber directly after extrusion (Figure 6c). The velocity of gas flow in the evaporating chamber will be the dominant factor affecting the membrane morphology. Meanwhile, a melted polymer is used as the dope in melt spinning, and fiber is formed upon exposure to quenching air (Figure 6d). For the spinning techniques that involve the use of an air gap, the relative humidity in the air gap has a pronounced effect on the membrane porosity. As the nascent hollow fiber is exposed to a higher degree of humidity, the increased water content in the outer layer gives rise to a more porous structure which results in higher permeation rates [78,79]. The control of relative humidity in the air gap thus represents a pragmatic tool to induce a porous outer surface which may be of particular importance for the modification of surface properties such as wettability. Likewise, electrospinning is an electrostatic fiber fabrication technique that also utilizes melted polymer as polymer dope (Figure 6e). This technique produced fibers on the nanoscale by extruding the charged dope under a high-voltage electric field.

### 3.1. Organic and Inorganic Materials-Derived Hollow Fiber Membranes

Organic materials are suitable candidates for hollow fiber membrane fabrication as they are cheap, processable, and scalable. A surge of interest in developing polymeric hollow fiber membranes has been witnessed in the past few decades. Examples of polymers reported include polysulfone (PSF) [80,81,82,83], polyethersulfone (PES) [84,85,86,87], cellulose acetate [88,89,90], polyimide [91,92,93,94], polyetherimide [95,96,97,98], polycarbonate (PC) [99,100], polyvinylidene fluoride (PVDF) [83,86,101,102,103,104,105,106,107], and polybenzimidazole (PBI) [108,109]. The fabrication of polymeric hollow fiber membranes follows the common practice mentioned previously, which often includes a post-treatment such as silicon rubber coating to seal the defects on hollow fiber membranes for superior separation performance [110]. However, daunting challenges remain in polymeric-based membranes, including overcoming the trade-off of permeability/selectivity, producing an ultrathin selective layer with maximum transport efficiency, and robustness under harsh operating conditions.

Electroconductive polymers, also known as conjugated polymers, consist of extraordinary physical, chemical, and electrical properties due to the presence of delocalized π-bond. These polymers include polypyrrole, polyaniline (PANI), polyacetylene, polythiophene, polyphenylene vinylene, polyp-phenylene, and their other derivatives [111]. Among these conductive polymers, PANI, polypyrrole, and substituted polythiophene are highly stable even under long-term exposure to air [112,113,114,115].). These polymers carry charge in the presence of potential, benefitting from the alternative double bond (e.g., π-bond) and single bond (e.g., σ-bond). Furthermore, the conductive polymers are easy to be doped due to the presence of conjugated chain bonding [116]. As compared to conventional polymers, these polymers possess the advantages of a small electronic bandgap (e.g., 1–3.5 eV), easily modified by the charge transfer process, and having high electrical conductivity [117]. Hence, conjugate polymers are widely found in many applications, such as solar cells [118], fuels cells [119], gas sensing [120], and particularly the synthesis membranes [121,122]. Their fabrication in hollow fiber configuration remains challenging, while only a handful of research was reported for liquid and gas applications that will be covered in the later section.

**Figure 7:**
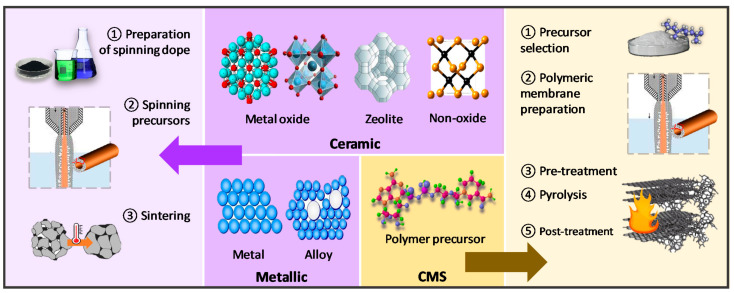
The categorization and preparation routes of inorganic hollow fiber membranes.

Inorganic materials such as ceramic, metallic, and carbon derivatives have received adequate attention as membrane materials due to their explicit mechanical and thermal stabilities. Ceramic hollow fiber membranes are commonly fabricated from metal oxides, such as alumina [123,124,125], perovskites [126,127,128,129], zeolites [130,131], kaolin [132], and non-oxides, such as silicon carbide [133]. Correspondingly, ceramic membranes show outstanding stability against extreme temperature, pH, and fouling conditions. Metallic hollow fiber membranes are recognized for their harsh thermal and mechanical properties, for instance, stainless steel [134], and catalytic transition metals such as nickel [135] and nickel-based alloy [136], which can be integrated into membrane reactor applications. Carbon-based materials are another important class of inorganic materials to be derived into hollow fiber membranes with excellent size-sieving characteristics [137,138,139,140,141,142,143,144,145]. The types of inorganic hollow fiber membranes and their preparation routes are summarized in Figure 7.

The preparation of ceramic and metallic hollow fiber membranes follows the four principal steps: (1) preparation of the spinning dope, (2) spinning of precursors, (3) phase inversion, and (4) sintering [58]. Generally, the spinning dope is prepared by milling the ceramic or metal powder into desired particle size, followed by the addition of solvent, dispersant, polymer binders, and plasticizer. The particle size, shape of the precursor powder, and dispersibility will affect the final membrane morphology and performance. Correspondingly, the dispersant aids in breaking any soft agglomerates within particles and enhances homogeneity [146]. The binder to plasticizer ratio is adjusted to improve the flexibility of precursor dope that eases the fiber formation during the spinning process. After degassing, the dope is spun, and the fiber is coagulated in non-solvents, often water, for NIPS. Following that, the sintering step is essential to convert loose fine particles into a coherent mass without melting to the point of liquefaction, resulting in the densification, grain coarsening, and pores closing of the hollow fibers [147]. There are three main steps in sintering, namely pre-sintering, thermolysis, and final sintering. For instance, the hollow fibers are sintered at 400–800 °C for 1–2 h to burn off the polymer binder via thermolysis, then raised to the final sintering temperature of 1000–1400 °C for 1–8 h [123,131,134,135]. Perovskite-based hollow fiber membranes are directly subjected to the final sintering condition since they are synthesized without polymeric binders [126,127,128,129].

On the other hand, CMS hollow fiber membranes are prepared via a more complex strategy. Pristine polymeric hollow fibers are prepared before pre-treatment, pyrolysis, and followed post-treatment. Polymeric precursors shall possess dominant characteristics such as flexible molecular arrangement and thermal resistance that favor tuning the membrane morphology and optimizing the separation performance. Some polymer precursors such as cellulose [137], PBI [144], polyimides [138,139,140,141,142,143], and polymers of intrinsic microporosity (PIMs) [148,149,150] are frequently used to prepare CMS hollow fiber membranes. Following that, the polymeric hollow fibers were pretreated at 200–300 °C to stabilize the cross-linked polymer chains to withstand the pyrolysis at around 500–1000 °C [146]. The pyrolysis process is the key step in the preparation of CMS hollow fiber membrane to activate sieving properties through carbonizing the membrane pore structure. Moreover, the final pore size of CMS hollow fiber membranes can be increased and reduced through post-oxidation [151,152] and post-pyrolysis [140,141,153], respectively.

### 3.2. Composite Hollow Fiber Membranes

**Figure 8:**
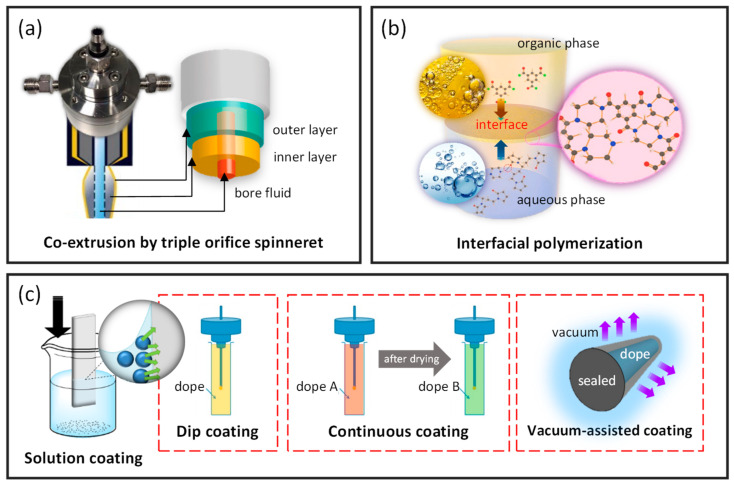
Various composite hollow fiber membranes fabrication techniques, including (**a**) co-extrusion, (**b**) interfacial polymerization, and (**c**) different solution coating methods.

There are several composite hollow fiber membranes, including dual-layer, TFC, thin film nanocomposite (TFN), polymer blends, and mixed matrix prepared via the incorporation of various distinctive materials. Figure 8 summarizes the preparation routes of these composite hollow fiber membranes.

Dual-layer hollow fiber membranes are made up of a microporous supporting inner layer with a dense-selective outer layer, or vice versa, prepared via direct spinning of two polymer dope solutions. The support layer shall have a smooth surface and high porosity to minimize the transport resistance as well as substructure resistance [7,154]. The bi-layer structure in these membranes is simultaneously formed via the co-extrusion method using a triple-orifice spinneret (Figure 8a) during the spinning process [7,27,51,155,156], whereby the dual-layer interface should be carefully handled to avoid delamination. Dual-layer hollow fiber membranes offer attractive advantages such as cost- and time- effectiveness where cheap materials are used as a support layer and a small amount of novel polymer as the selective layer prepared by a one-step co-extrusion process [12].

TFC membranes consist of a thin film of a selective layer loaded on top of a porous substrate. The TFC membranes have a high mechanical stability and cost-effectiveness as they require only a low amount of material as the dense selective layer of less than 100 nm, for instance, approximately 4 wt% [7]. TFC hollow fiber membranes are commonly prepared via interfacial polymerization [157] and dip coating [34,158,159,160,161,162]. Interfacial polymerization is performed by loading two immiscible solutions (e.g., aqueous and organic phases) on the inner or outer surface of the nascent hollow fiber (Figure 8b). The amine groups from the aqueous phase will react with the acyl chloride group in the organic phase through nucleophilic substitution, thus forming an ultrathin polyamide selective layer. To increase the flux of TFC membranes, they are post-treated with protic acids such as concentrated formic acid, leading to the hydrolysis of the polyamide film [163]. Another common post-treatment process is the curation of membranes in the oven for a short duration [164,165]. Sun and Chung proposed a vacuum-assisted interfacial polymerization where the vacuum was applied from the lumen side of the hollow fibers before they were immersed in the organic phase solution to maintain the trans-membrane pressure [166]. Recently, a continuous outer-selective interfacial polymerization was developed to achieve performance controllability and mass production [167]. The setup comprises an unwinder, tension meter, circular air knives, wetting unit (aqueous solution), reaction unit (organic solution), curing unit, and winder. At the end of all these interfacial polymerization processes, the membranes are kept in deionized water before use. These TFC membranes are widely utilized in various applications such as water treatment, pervaporation, gas separation, and energy production [7,168]. Meanwhile, the dip coating method is performed by immersing a nascent hollow fiber into a coating solution to obtain a desired selective layer (Figure 8c). In addition, introducing a gutter layer between the hollow fiber substrate and selective layer is indeed beneficial for enhancing their adhesion strength and minimizing solution intrusion [169]. Other solution coating methods, such as continuous coating [70,170] and vacuum-assisted coating [171,172], have also been reported (Figure 8c).

Apart from that, polymer blends are another form of composite membranes that combine the synergistic properties of different materials within the matrix to achieve targeted performance by overcoming the deficiencies of the individual components [173]. It has the advantages of simplicity, reproducibility, and cost-effectiveness. The preparation route of polymer blends starts with the selection of a proper parent polymer with desired physical and chemical properties to complement another polymer. The composition of polymer blends requires proper adjustment to form a miscible blend with desired characteristics, followed by the spinning of the polymer dope with phase inversion [174,175,176,177,178].

In order to diversify the practical application of polymeric hollow fiber membranes, nanofillers are frequently introduced into the polymer matrix to develop advanced hybrid membrane systems with robust performance. Nanofillers can be introduced into hollow fiber membranes by adding the fillers into spinning dope and extruded in a dual- or triple-orifice spinneret [179,180,181] or by adding the fillers into the selective layer forming a thin nanofillers-incorporated film on the surface or in the lumen side of the porous hollow fiber support [182,183,184,185]. The composite membrane fabricated by the former strategy is known as a mixed matrix hollow fiber membrane, while the latter is TFN hollow fiber membrane. Correspondingly, various nanomaterials have been reported in composite hollow fiber membranes, such as metal oxides [186,187,188,189], zeolites [190,191,192], silica [193,194,195], metal organic frameworks (MOFs) [196,197,198,199], two-dimensional (2D) materials (e.g., graphene oxide (GO) and MXene) [200,201,202,203,204], and mixed nanofillers [205,206,207]. The incorporation of nanofillers alters the mobility of polymer chains, induces interfacial voids between the two phases, enhances sorption and affinity toward certain species, and hence ameliorates membrane performance.

## 4. Applications of Hollow Fiber Membranes

### 4.1. Microfiltration (MF)

MF is a pressure-driven separation process via a semi-permeable membrane, where the pore sizes can range from 0.1–1.0 μm. It is widely utilized to purify or separate macromolecules and suspended particles from a liquid mixture [208]. MF separation process is often employed for wastewater treatment [209,210] and the purification of process streams for biotechnology purposes [211,212,213].

**Figure 9:**
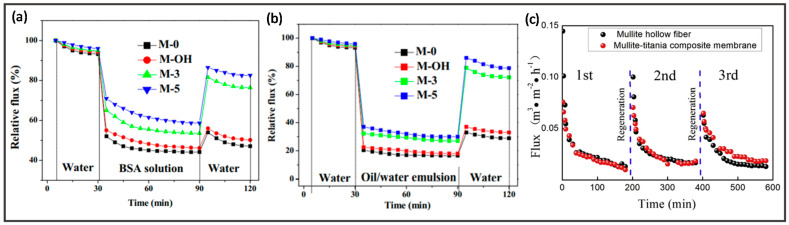
Time−dependent relative flux during filtration of (**a**) BSA solution and (**b**) oil/water emulsion. Reprinted/adapted with permission from Ref. [214]. Copyright 2016, Elsevier. (**c**) Variation of permeation flux with time during membrane filtration of oil/water emulsion using membrane regeneration for three cycles via a backflushing of 0.1 wt.% NaOH aqueous solution. Reprinted/adapted with permission from Ref. [216]. Copyright 2016, Elsevier.

Aslan et al. synthesized a novel polyacrylonitrile nanofiber electrospun membrane that demonstrated excellent water flux coupled with high turbidity, total organic carbon, and UV_254_ removal efficiencies of 95%, 25%, and 45%, respectively [42]. In addition, zwitterionic poly(lysine methacrylamide) brushes were grafted onto PVDF MF membranes to impart fouling resistance toward protein and oil emulsion in wastewater (Figure 9a,b) [214]. Hernández et al. utilized water-based green chemistry to hydrophilize PVDF membranes using polyvinylpyrrolidone and persulfate, followed by functionalization with poly(acrylic acid) and iron (Fe)/palladium (Pd) nanoparticles, preventing the leaching and aggregation of nanoparticles [215]. The modified membrane showed superior catalytic activity in the reduction of trichloroethylene, which is a low biodegradability pollutant that contaminates the soil and groundwater.

On the other hand, hollow fiber membranes were also utilized in food industries, such as the filtration of protein and wine. Schopf et al. compared the fractionation efficiency of milk protein using hollow fiber membranes, ceramic tubular membranes, and spiral wound membranes [217]. The developed hollow fiber membrane had a high membrane packing density and low fouling tendency while maintaining excellent fractionation. To fractionate milk protein, the same research group discovered that the optimum length for the PES membrane was 0.6 m to achieve the highest flux and whey protein transmission [218]. Moreover, Giacobbo et al. made use of hollow fiber membranes to recover antioxidant compounds, namely polyphenols and polysaccharides, from winery effluents [219]. Mondal and De developed a polyacrylonitrile (PAN) copolymer-based hollow fiber membrane that could achieve 80% purity of total polyphenol content and 75% purity of epigallocatechin gallate in the permeate of green tea extract [220]. Furthermore, optimal conditions were discovered using the relationship between transmembrane pressure and Reynolds number to guarantee maximum throughput of the membrane.

Ceramic membranes have been employed in MF due to their high selectivity, excellent thermal and mechanical stability as well as outstanding resistance to high oil concentration and robust cleaning chemicals [221]. Aziz et al. developed a cost-effective hollow fiber membrane from naturally occurring ball clay which demonstrated good mechanical strength and water flux [222]. Apart from that, ceramic membranes demonstrated superior antifouling properties compared to polymeric membranes using three common types of organic foulants that are humic acid, sodium alginate, and bovine serum albumin (BSA), which is also proven by the Extended Derjaguin-Landau-Verwey-Overbeek (XDLVO) theory [223]. In one study, composite ceramic membranes showed an enhancement in separation performance via the enrichment of functionalities in the active layers [224]. For example, silica nanoparticles were coated onto Si_3_N_4_ hollow fiber membranes to treat oily wastewater [225]. The resultant membrane rejected submicron contaminants effectively and mitigated membrane fouling. In addition, researchers added polyethylene glycol (PEG) with different molecular weights as a pore agent into the kaolin hollow fiber membranes [226]. The mechanical strength and oil rejections were improved using this modification. By recycling coal fly ash as the raw material for membrane support, Zhu et al. prepared a multi-layer-structured titanium dioxide (TiO_2_)/mullite membrane, which displayed excellent separation and could be easily regenerated via sodium hydroxide (NaOH) solution backwashing, as shown in Figure 9c [216]. This not only reduces the cost of membrane fabrication but also solves environmental issues.

### 4.2. Nanofiltration (NF)/Organic Solvent Nanofiltration (OSN)

NF membrane selectively separates small organic molecules and multivalent ions through steric hindrance and the Donna effect, respectively. It segregates them with a molecular weight cut-off (MWCO) between the range of 200 and 1000 Da [227] and consumes low operating pressure, thus requiring lesser energy as compared to RO [228,229,230].

Hollow fiber NF membranes have been widely used in various applications such as wastewater treatment [231,232], food and beverage industry [233,234], chemical and petrochemical industry [235,236,237,238,239], and biorefinery [240,241]. Dynamic deposition technology was introduced to develop robust membranes under real-time performance monitoring. Wang et al. synthesized a negatively charged hollow fiber NF membrane via dynamic deposition of zirconium dioxide (ZrO_2_) sols on P84^®^ polyimide substrates [242]. The synthesized membrane exhibited excellent mechanical strength and was able to withstand high pressure while maintaining a high sodium sulfate (Na_2_SO_4_) rejection of 93.4% due to its high hydrophilicity and negative charge. Furthermore, it showed a high rejection of >90% toward antibiotics and traditional Chinese medicines such as tea polyphenols, puerarin, tetracycline, and rifampicin. Besides negatively charged hollow fiber membrane [242], He et al. synthesized a positively charged membrane using chitosan lactate via interfacial polymerization [243]. As compared to the previous study, this membrane demonstrated slightly higher magnesium chloride (MgCl_2_) and zinc chloride (ZnCl_2_) rejections (e.g., 95.1% and 95.7%, respectively) due to the positively charged surface of the membrane. Moreover, it possesses a high potential for industrialization due to the short interfacial polymerization time and the use of chitosan lactate which is low cost, non-toxic, and environment friendly.

Other than charged membranes, conjugated polymers are also utilized to synthesize NF membranes. However, a limited study was performed on utilizing conjugated polymers to synthesize hollow fiber membranes due to their low mechanical strength. Instead, there are some studies on the preparation of flat sheet membranes via conjugated polymers for NF, such as polypyrrole/multiwalled carbon nanotubes (MWCNTs) membranes in treating Reactive Blue 50 dye [244] and conductive PANI-coated PVDF composite NF membranes with tunable separation selectivity prepared through in situ chemical oxidative interfacial polymerization [245]. Ma et al. synthesized conjugated PANI derivative membranes via polymerization of ammonia-rich monomers and a post-crosslinking process [246]. In this case, poly-*p*-phenylenediamine (PpPD)/hydrolysed PAN NF membranes were synthesized using PpPD with conjugate structure via in situ growth onto a porous substrate. As a result, the membrane exhibited an excellent water permeance of 297 LMH/bar with Congo Red (CR) or Direct Red 23 (DR23) rejections above 98.5% due to the co-effects of size sieving and charge repulsion.

**Figure 10:**
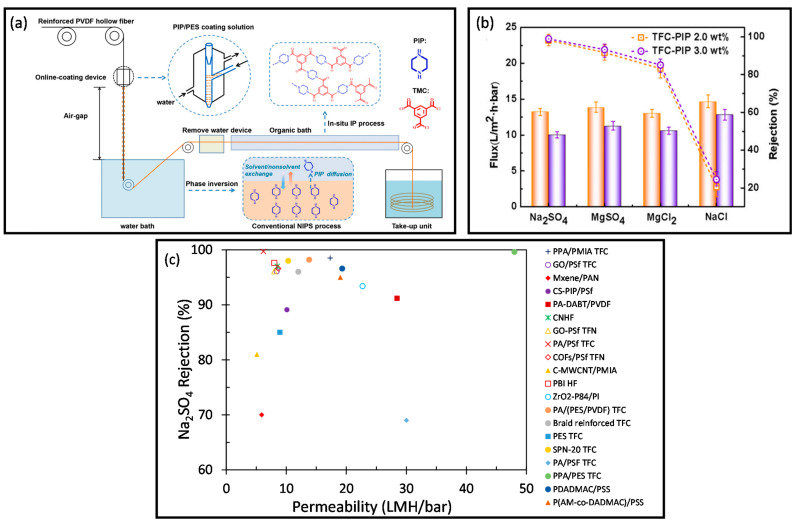
(**a**) The illustration of the synthesis procedure of PA/(PES/PVDF) TFC hollow fiber membrane and (**b**) various salt rejection of PA/(PES/PVDF) membrane. Reprinted/adapted with permission from Ref. [86]. Copyright 2021, Elsevier. (**c**) Performance plot of Na_2_SO_4_ rejection in nanofiltration.

In addition, hollow fiber membranes are also being fabricated into TFC membranes. Wang et al. have fabricated a TFC hollow fiber NF membrane, known as polyamide (PA)/(PES/PVDF), via in situ interfacial polymerization procedure with PA functional layer, PES coating layer, and PVDF supporting layer, as shown in Figure 10a [86]. The synthesized membrane exhibited high pure water permeability (PWP) (e.g., 13.8 LMH/bar) and Na_2_SO_4_ rejection (e.g., 98.2%) (Figure 10b) [86], which was comparable to other hollow fiber TFC membranes synthesized using various methods such as conventional interfacial polymerization [247,248] and construction-interlayer [249]. As compared to other synthesis methods, the reported method was found to be facile, scalable, cost-effective, and did not require any post-treatment, resulting in a simple and economical strategy for industrializing the production of TFC hollow fiber membrane. Recently, 2D materials have also gained attention due to their unique physicochemical properties, atomic thickness, and defined nanochannels [250]. For instance, Liu et al. utilized metal carbides and nitrides (MXene) to synthesize hollow fiber membranes for removing divalent ions from water [251]. Although the synthesized MXene/PAN membrane possessed lower Na_2_SO_4_ rejection of ~70% as compared to the aforementioned studies, the high packing density in the hollow fiber membrane module and the modifiable structure of the MXene laminar layer showed the potential to improve the performance of the membrane and the scale-up of MXene in the desalination process.

In terms of wastewater treatment, real industrial wastewater generally consists of multiple contaminants, such as various inorganic salts, heavy metals, and dyes, which can be reused and recycled [252,253]. However, normal NF membranes are difficult to fractionate the dye/salt mixtures due to smaller particle sizes [254]. Hence, loose NF hollow fiber membranes with high rejection of dyes and low rejection of salts are developed. For instance, Chu et al. have fabricated a reinforced PES loose NF hollow fiber membrane for textile wastewater treatment via a facile dry-wet spinning process [254]. The as-synthesized membrane possessed a high pure water flux of 8.72 LMH/bar with high CR rejection (e.g., 99.9%) and low sodium chloride (NaCl) rejection (e.g., <7%). Another study showed the synthesis of loose NF hollow fiber membrane using polyethyleneimine (PEI) to reject small molecule dyes, such as crystal violet and methyl orange, with a molecular weight of 407.0 g/mol and 327.3 g/mol, respectively [255]. The membrane exhibited excellent dye rejection of ~100% due to size sieving with permeate flux of ethanol of 10.4 LMH/bar.

In addition, fouling is another typical problem faced by NF membranes. Hence, some research has been done to improve the antifouling property of membranes. For instance, Wang et al. synthesized a high-flux TFC hollow fiber membrane for the desalination process using BSA, humic acid, emulsified oil, and Rhodamine B as fouling sources [86]. The flux recovery ratio (FRR) for BSA, humic acid, and emulsified oil were 94.8%, 92.3%, and 88.7%, respectively, with an irreversible pollution ratio below 11.5%, showing that the synthesized membrane possessed superior antifouling property. In addition, Shi et al. incorporated hydrophilic and compatible poly(*p*-phenylene terephthalamide) (PPTA)/PSF microparticles into the PA functional layer to synthesize a hollow fiber composite membrane [256]. After 6 h of dye filtration, the resultant membrane experienced a reduction of normalized water flux from 99 to 85%. However, the normalized water flux could be effectively recovered to 96.8% after cleaning with deionized water. Additionally, the PPTA/PSF incorporated PA hollow fiber membrane exhibited slower fouling compared to the pristine membrane. With the development of membranes with excellent antifouling properties, the potential of commercializing these membranes can be realized due to the reduction in cost and time required to clean and replace the membranes.

Figure 10c shows the Na_2_SO_4_ separation performance plot of various membranes. The PPA/PES TFC exhibited the best performance with the highest Na_2_SO_4_ rejection and permeability. It was due to the presence of interlayers as amines storage, which controlled the reaction between amines and acryl chlorides to form a thin PA layer [167]. Additionally, the use of PVA to manipulate the diffusion of monomer also enhanced the membrane performance owing to the Turing structure [257]. As observed from the plot, most of the membranes had a high Na_2_SO_4_ rejection of >90% with a moderate permeability within the range of 5 to 20 LMH/bar. Among these membranes, the MXene/PAN showed the lowest Na_2_SO_4_ rejection and permeability. In short, the development of membranes with high rejection and permeability should be promoted to enhance water permeance while maintaining high rejection performance.

Organic solvent nanofiltration (OSN) is a separation process whereby the membrane allows the passage of small solvent molecules while rejecting solutes with a molecular weight between 200 and 2000 Da [258]. OSN is categorized into solvent–solute separation, solute purification and solvent exchange, and it holds great potential as it can lower energy consumption, prevent degradation of sensitive molecular products, and minimize waste generation [54,259,260]. To ensure stability in organic solvents, the common materials for OSN membrane fabrication are polyimide, PAN, and PBI.

Polyimide membranes with various properties and solvent resistances have been investigated to surpass the commercial Starmem membranes [260]. For instance, poly(4,4′-oxydiphenylene pyromellitimide) (PMDA-ODA) polyimide membranes were synthesized by spinning PMDA-ODA polyamic acid (PAA) into hollow fibers, followed by chemical imidization [261]. In addition, a gelation/NIPS method was employed in synthesizing pyromellitic dianhydride-4,4′-diaminodiphenylmethane (PMDA-MDA) PAA/TiO_2_ mixed matrix membranes to reduce the formation of macrovoids in the membranes [262]. The gelation of PAA substantially increased the permeability of the membrane, while the addition of TiO_2_ in the dope enhanced the rejection of Rose Bengal (RB) in tetrahydrofuran (THF), dimethylsulfoxide (DMSO), and dimethylformamide (DMF) due to the densification of the skin layer. Goh et al. developed a solvent resistant polyimide HF membrane through interfacial polymerization, where its rejection could reach 91.8% and 99.9% for acid fuchsin and RB, respectively [263]. A solvent-activated TFC membrane obtained excellent permeability for different organic solvents while maintaining high selectivity [264]. This membrane holds promise in the pharmaceutical field due to its capability of concentrating levofloxacin in acetone. Apart from polyimide, PBI is another popular membrane material for OSN as it possesses excellent thermal and chemical stability [265,266,267]. Recently, Zhao et al. performed a green modification method (at room temperature and in an aqueous solution) to fabricate a PBI OSN membrane by cross-linking PBI with potassium persulphate [108]. The resultant membrane displayed outstanding stability in harsh solvents such as *N*-methyl-2-pyrrolidone (NMP), DMF, and DMSO and could be regenerated via methanol backwashing. 

Furthermore, conductive polymers are alternative materials for OSN membranes due to their advantages, such as high permeability and selectivity in separating small molecules from organic solvents. Currently, conductive polymers are employed to synthesize flat sheet membranes, while there are no reported works on hollow fiber membranes. A p-phenylenediamine/hydrolyzed PAN membrane displayed outstanding ethanol permeance of 242 LMH/bar with high rejection for various dyes, which was further evaluated using a two-stage OSN process [246]. Contrary to the commonly used polyamide materials, the conjugated microporous polymers (CMPs) are rich in permanent and interconnected micropores in 3D networks consisting of rigid π-conjugated backbones [268]. A synthesized CMP membrane could achieve higher permeance of non-polar solvent (e.g., hexane) compared to the conventional polar polyamide membrane [269]. Interestingly, the rigid structure of CMP membranes leads to a high structural rigidity, which resulted in a relatively high surface area and permanent pores, which are crucial for ultrafast OSN. Through postoxidation of the thiophene moieties, the pore size of the CMP membrane was fine-tuned at the angstrom scale to control its selectivity [270]. Furthermore, the CMP membranes exhibited excellent stability, for instance, (1) permeance stability in different organic solvents for a week [271], (2) chemical stability which was proven by the filtration performance after soaking the membranes in different organic solvents for one week [269], and (3) thermal stability up to 300 °C [269,271].

To further improve the separation performances of OSN membranes, nanomaterials such as MWCNT [272], molybdenum disulfide (MoS_2_) [273,274], graphene quantum dots [275,276], zeolitic imidazolate framework (ZIF)-8 [277,278,279] and GO [280,281,282] have been incorporated either into the membrane substrate or the selective layer. However, most of the modifications were carried out on flat sheet membranes, while hollow fiber TFN membranes were not widely studied. Su et al. doped GO nanosheets into hollow fiber TFN membranes to improve their permeance, rejection, and swelling resistance [283]. The resultant membrane also demonstrated great solvent and pollution resistance by rejecting >99% of Rhodamine B while maintaining similar ethanol permeance over 18 h of filtration. Similarly, GO-based nanohybrids decorated with TiO_2_ nanoparticles were incorporated into TFN membranes to enhance dye rejection and antifouling properties [284]. The permeate flux of the TFN was improved not only by the hydrophilicity of the nanohybrids but also by the formation of additional pathways in the dense layer as well as the solvent channelization through the gaps of GO nanosheets. In another work, a PI membrane was modified with amine-functionalized carbon nanotubes followed by cross-linking to enhance its mechanical properties, thermal stability, and solvent permeances [285]. The membrane possessed the ability to separate tetracycline/isopropanol, L-α-lecithin/hexane, and 2, 2-bis(diphenylphosphino)-1, 1-binaphthyl)/methanol solutions, demonstrating its potential in pharmaceutical, food, and petrochemical industries. Table 1 and Table 2 tabulate the separation performances in NF and OSN for hollow fiber membranes, respectively.

### 4.3. Reverse Osmosis (RO)/Organic Solvent Reverse Osmosis (OSRO)

RO is one of the most effective commercialized technologies in water and wastewater treatment. Unlike UF and NF technologies, RO technology can separate monovalent ions such as sodium ions (Na^+^) and chloride ions (Cl^−^) from water or wastewater through a semi-permeable membrane. As compared to FO, RO treatment is equipped with higher external pressure than the osmotic pressure, forcing the water molecules to flow in the opposite direction. Hence, compared with other conventional water or wastewater treatment and seawater desalination, RO is energy-efficient in producing high-quality freshwater due to its membranes with high permeability and selectivity [286,287].

**Figure 11:**
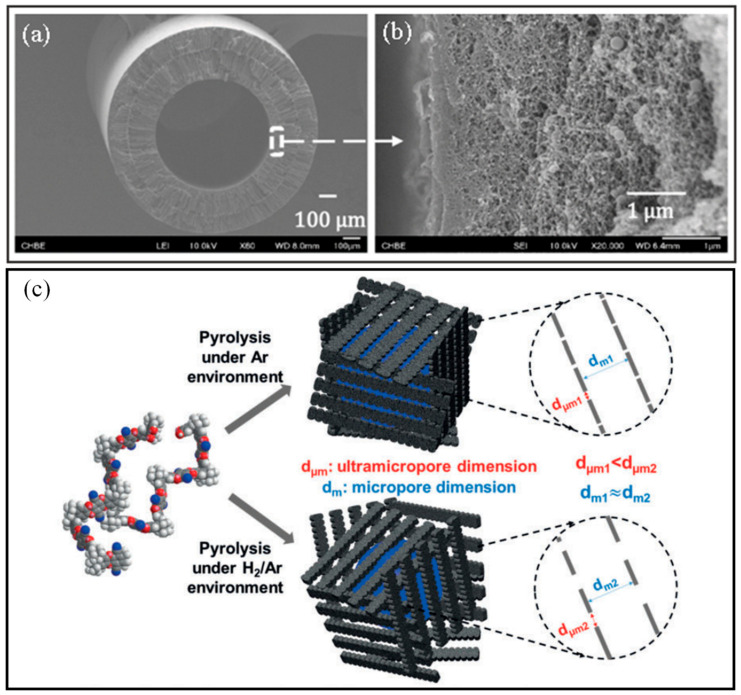
FESEM images of the (**a**) cross-section and (**b**) inner edge morphology of hollow fiber RO membranes with the highest flux. Reprinted/adapted with permission from Ref. [288]. Copyright 2021, Elsevier. (**c**) Illustration demonstrating the introduction of H_2_ to the pyrolysis environment leading to an enlargement of the ultramicropores with only minor changes in the size of the micropores. Reprinted/adapted with permission from Ref. [149]. Copyright 2019, Wiley.

Yang et al. synthesized thin-film composite (TFC) hollow fiber membranes with a defect-free PA selective layer for brackish water reverse osmosis (Figure 11a,b) [288]. The synthesized membrane exhibited high water permeability of 4.49 LMH/bar and NaCl salt rejection of ~99% under 20 bar. The membrane even showed high stability by maintaining normalized flux above 85% after 15 h of filtration. In addition, nanomaterials with unique and outstanding characteristics such as hydrophilicity, molecular-sieving selectivity, and chemical resistance were also introduced into RO membranes [289,290]. Gai et al. fabricated TFC membranes by combining amino-functionalized carbon quantum dots (CQDs) and hypochlorite treatment [291]. With the incorporation of amino-functionalized CQDs, the PWP of the membranes was at 5.5 LMH/bar with NaCl rejection of 98% under 15 bar. After the hypochlorite treatment, the PWP of the membranes further increased to 10.13 LMH/bar with NaCl rejection of 98.9%, resulting from a less cross-linked PA layer and greater charge repulsion effect against charged solutes. Other than CQDs, 2D materials such as layered double hydroxide, anionic clay materials (empirical formula: [M_1−x_^2+^M_x_^3+^(OH)_2_][A_x/n_^n−^]·mH_2_O) with positively charged brucite-like metal hydroxide layers intercalated with counterpart anions [292], were also incorporated into RO membranes owing to its flexible and tunable chemical composition [293].

Besides desalination, RO hollow fiber membranes are also being used for effluent treatment in a pharmaceutical plant. For instance, Liu et al. synthesized an antifouling RO membrane via a simple irradiation strategy to graft hydrophilic polymers on the membrane surface without the need for any catalyst and initiator [294]. The PA membrane with poly(vinyl alcohol) (PVA) modifier concentration of 1.0% exhibited outstanding hydrophilic properties and high salt rejection. Owing to the hydrophilicity and roughness of the membrane, the irreversible flux decline rate and FRR of the membrane were 1.7% and 98.3%, respectively. The membrane even possessed high foulant removal efficiency (e.g., the chemical oxygen demand of 99.5%) in an actual pharmaceutical plant effluent treatment after continuous operation for 28 days. To harvest more water and increase the concentration of RO brine, osmotically assisted reverse osmosis (OARO) has been developed [295,296,297]. Unlike the normal RO technique, OARO is a pressure-driven process combining the principles of both FO and RO [298], which often operates at extremely high pressures that most commercialized membranes are unable to [295,296]. For instance, Askari et al. developed a TFC hollow fiber membrane consisting of a PA layer on the inner surface of the PES hollow fiber substrate [298]. The synthesized membrane exhibited PWP around 3 LMH/bar and NaCl rejection of 97.5% to 98% for desalination of brackish water at 20 bars. So as to study the mechanical strength of the membrane, the structural parameter and burst pressure were used where the optimal values were 795 µm and 95 bars, respectively.

Over the past decade, organic solvent reverse osmosis (OSRO) has emerged as one of the most promising technologies to separate organic liquid mixtures due to its high energy efficiency. Compared to OSN, it has a greater molecular specificity with the ability to separate organic liquids, for instance, alcohols, alkanes, and aromatics [299]. There has been a number of works that studied OSRO using organic membranes [300,301,302], CMS membranes [148,149,303,304], silica-based membranes [305], and composite membranes [306,307,308], which yielded excellent results. However, most of these works focused on flat sheet membranes, while the research on hollow fiber membranes in the application of OSRO is still lacking.

Jang et al. fabricated a polyamide-imide, namely Torlon^®^ hollow fiber OSRO membrane, due to its superior mechanical, thermal, and chemical stability [302]. The fabricated membrane displayed excellent molecular separation, rejecting aromatic molecules as small as 185 g/mol from similarly sized solvents. Compared with commercial OSN polyimide membranes such as PuraMem^®^ and DuraMem^®^ membranes, Torlon^®^ membranes allow small alkyl aromatic separations that these OSN membranes are unable to accomplish. On top of that, this membrane has shown potential for industrial applications due to its scalable spinning process. Another type of solvent- and temperature-resistant membrane is the CMS membrane, which is synthesized via the pyrolytic decomposition of polymeric materials [309]. When fabricated into hollow fibers, these membranes are capable of withstanding high transmembrane pressures. Lively et al. achieved molecular separation of *para*-xylene (5.8 Å) from *ortho*-xylene (6.8 Å) via pyrolysis of cross-linked PVDF hollow fiber membrane [304]. The fabricated CMS membrane was capable of enriching an equimolar liquid feed to 81 mol% *para*-xylene, which corresponded to a separation factor of 4.3 without any phase change. In another work, Ma et al. studied the pyrolysis of the PIM-1 precursor under a mixed hydrogen (H_2_)/argon atmosphere to create “mid-sized” microstructures in CMS membranes (Figure 11c) [149]. Interestingly, these changes in the ultramicropore size drastically improved *para*-xylene permeability while maintaining excellent *para*-xylene/*ortho*-xylene selectivity through Wicke-Kallenbach tests under the conditions of low-pressure vapors. As such, these facile and straightforward pyrolysis methods are significant in paving new pathways for membrane-based applications of CMS materials.

### 4.4. Forward Osmosis (FO)/Organic Solvent Forward Osmosis (OSFO)

FO is an emerging process that uses FO membrane to separate water from feed solution (e.g., higher water chemical potential) to draw solution (e.g., lower water chemical potential) by natural gradient osmosis [310]. As a result, diluted draw solution and concentrated feed solution will be obtained. Forward osmosis has been widely applied in various fields such as wastewater treatment, desalination, and solvent recovery because of less energy consumption and high salt rejection [59]. Two critical factors that can significantly influence the FO technology are the membrane module and draw solutions [311,312]. Recently, efforts such as chemical grafting and the addition of highly porous particles have been demonstrated to improve water flux and salt rejection in hollow fiber membranes. 

**Figure 12:**
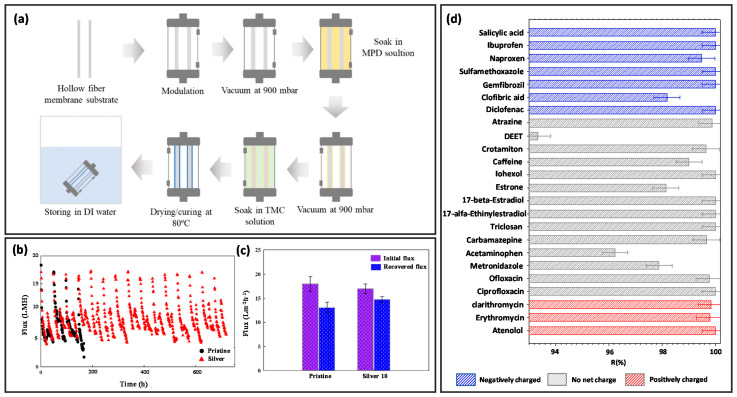
(**a**) Vacuum-assisted interfacial polymerization for thin film nanocomposite hollow fiber membrane. (**b**) Fouling test of the Ag-loaded membrane and pristine membrane was conducted for 30 days. (**c**) Comparison of flux recovery after the dewatering test followed by 1 h of salt cleaning. Reprinted/adapted with permission from Ref. [315]. Copyright 2022, Elsevier. (**d**) Performances of aquaporin hollow fiber membrane on CECs rejection. Reprinted/adapted with permission from Ref. [324]. Copyright 2021, Elsevier.

Several studies introduced nanoparticles into the active layer of the membrane to enhance the stability and lower the tendency of fouling, including GO [313,314], silver (Ag) nanoparticles [315], zeolite [316], carbon nanotube [317], aquaporins [318,319], ZIFs [320], and covalent organic frameworks (COFs) [321]. Choi et al. employed vacuum-assisted interfacial polymerization to incorporate Ag nanoparticles into outer selective-layer hollow fiber (Figure 12a) [315]. The Ag-incorporated membrane had better stability which lasted longer than 30 days and had higher fouling resistance than the pristine membrane (Figure 12b). In addition, the Ag-loaded membrane achieved 90% flux recovery, higher than the pristine membrane, which was only about 80% (Figure 12c). Aquaporins are another promising material that can be introduced into the active layer of hollow fiber membrane for enhancing separation performance, chemical resistance, and anti-fouling properties [322]. Sanahuja-Embuena et al. synthesized a Pluronic nanocomposite hollow fiber membrane by adding aquaporin into the active layer of the membrane in an osmotic membrane bioreactor [323]. The resultant membrane was rendered with synergistic effects in decreasing reverse solute diffusion from 0.36 gL^−1^ to 0.12 gL^−1^ without compromising the water flux. Contaminants of emerging concern (CECs) are harmful organic pollutants that are present in substantial amounts in pesticides, fertilizers, pharmaceuticals, and personal care products. Recently, Salamanca et al. applied FO to remove 24 types of CECs using their fabricated aquaporin-based hollow fiber membrane [324]. All the CECs were rejected by at least 93%, and particularly, 19 of them were rejected by about 99% (Figure 12d).

Conductive FO membranes are reported as promising anti-fouling membranes where the surface charge on the FO membrane repulse charged foulants in water via electrostatic repulsion [325]. Among the conductive materials, graphene nanosheets [326], MXenes [327], and PANI [328,329] have been studied in FO application, owning to adjustable conductivity and high mechanical and environmental stability [330]. They have been developed into a TFC membrane by coating on a PSF substrate. With the highly porous and hydrophilic nanostructure, this membrane effectively oxidized the targeted organic compounds and electrostatically removed the fouling layer [331]. However, there is no work reported on using conductive polymer in the fabrication of hollow fiber membranes for FO application.

Selecting a suitable draw solution is a crucial consideration in FO application. An ideal draw solution should give high water flux, low reverse solute flux, low fouling tendency, and high salt rejection. Monovalent draw solution, e.g., NaCl, is commonly used in wastewater treatment because it is abundantly available and cheap [332]. Not only that, the NaCl solution improves water flux and osmotic pressure in the FO process due to the small hydrated radius of the Na^+^ ion, which induces fast diffusion across the membrane, resulting in an effective driving force for water permeation [333]. However, several studies found that this kind of monovalent draw solution can result in high reverse solute flux and poor salt rejection [334]. Therefore, alternative solutions were studied to improve the performance of hollow fiber membranes in the FO process. Mohammadifakhr et al. reported that using a trisodium citrate solution draw solution yielded the membrane with a high separation performance with high water flux of 7.8 L/m^2^·h and a low reverse salt flux of 2.1 g/m^2^·h [335]. Huang et al. employed fertilizer as a drawn solution in polluted water treatment [336]. The diluted fertilizer can be applied to agriculture which could reduce the water consumption from crop irrigation. Almoalimi et al. found that FO separation using non-ionic draw solutions such as glucose, glycine, and ethanol demonstrated almost 100% of salt rejection but with a lower water flux due to a lower anion diffusion coefficient [337].

Other than draw solutions, tailoring in module design to optimize energy efficiency and fouling resistance have been studied. The module design modifications include configuration [338], length of module [339], operating parameter [340], and use of different operation modes [341] and the transverse vibration [342]. Akhtar et al. reported that the separation performance of FO membrane with counter-current flow configuration had a 7% higher water flux than the co-current flow configuration [338]. Some studies revealed that a long hollow fiber membrane module could reduce energy consumption and decrease water flux [339,340]. Next, vibrating submerged membranes received increasing attention because turbulence created on the membrane surface effectively interrupts the fluid boundary layer, thus giving better fouling control [343]. Low et al. revealed that using transverse vibration can significantly improve water flux while inhibiting the external concentration polarization of hollow fiber membranes [342].

Organic solvent forward osmosis (OSFO) is a neoteric promising process that concentrates the feed solution while transporting the organic solvent to the draw solution by applying the same principle of FO [344,345]. Unlike the aforementioned pressure-driven processes such as OSN and OSRO, OSFO does not require external hydraulic pressure but operates based on an osmotic pressure gradient. Therefore, OSFO saves on operation and maintenance costs [346]. Goh et al. demonstrated the potential of OSFO for pharmaceutical concentration [347]. The fabricated hollow fiber membranes showed a high acetone flux and low reverse solute flux when PEG-400 in acetone was used as a draw solution for the levofloxacin concentration process. Seo et al. reported CMS membranes derived from 4,4′-(hexafluoroisopropylidene) diphthalic anhydride (6FDA)-based polyimide precursors for liquid-phase hexane isomers separation. The ultramicropore membranes successfully separated hexane isomers with a sub-0.1 nm size difference by employing draw solutions as driving forces [348]. Designs of flat sheet membrane material for the OSFO process have been reported in the literature, such as Ti_3_C_2_T_x_ MXenes [349] and GO [350] nanosheets interlayered membrane. However, there is still limited work on hollow fiber membranes in OSFO application. Therefore, more studies are required to promote the use of hollow fiber membranes in OSFO.

### 4.5. Pervaporation

Pervaporation is renowned for its capability to separate azeotropic mixtures by selective partial vaporization [52]. Its separation efficiency depends on the preferential sorption and diffusion of the liquid feed components across the membrane selective layer, while the target component emerges as vapor permeate driven by vacuum or sweep gas [351]. In comparison with the conventional distillation process, pervaporation has overcome the limitation of thermodynamic vapor-liquid equilibrium with lower energy consumption, exhibiting favorability for the purification of thermally sensitive organics [352,353]. The pervaporation technology finds its major establishment in solvent dehydration of water-organic azeotropes, including water-alcohol [168,188,353,354,355,356,357,358,359,360,361,362,363,364,365,366,367,368,369,370], water-acetic acid [191,370,371,372,373], as well as organic solvents such as acetone, ethyl acetate, tetrahydrofuran, etc. [369,374,375,376,377,378]. Correspondingly, hydrophilic pervaporation membranes are favorable to this application owing to their selective transport of polar water molecules [379]. Other aspects of pervaporation applications, such as solvent recovery from organic-organic [380,381,382] and organic-water mixtures [199,383,384,385,386,387,388,389,390] and the removal of volatile organic compounds from wastewater [391,392,393,394] using hydrophobic membranes, have been well-reported.

**Figure 13:**
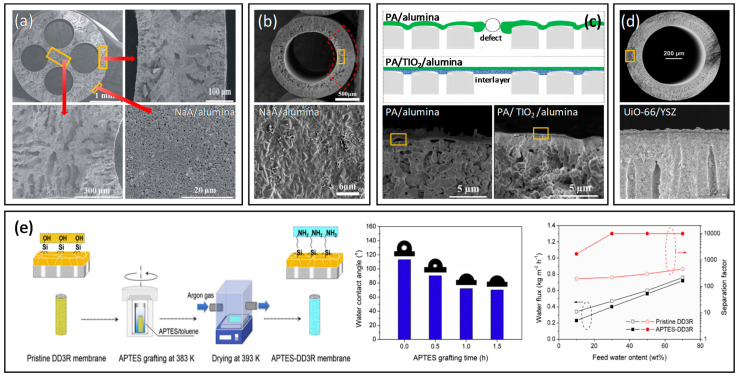
SEM images of NaA zeolites supported on the (**a**) outer surface and (**b**) inner surface of the alumina hollow fiber substrate. Reprinted/adapted with permission from Refs. [356,357]. Copyright 2015 and 2019, Elsevier and Royal Society of Chemistry. (**c**) The schematic and SEM images indicate the smooth and defect−free PA layer prepared on the TiO_2_ interlayer surface. Reprinted/adapted with permission from Ref. [188]. Copyright 2019, Elsevier. (**d**) SEM images of UiO−66/YSZ hollow fiber membranes. Reprinted/adapted with permission from Ref. [369]. Copyright 2017, Wiley. (**e**) The modification technique by grafting APTES to induce hydrophilic−functionalized surface and improve pervaporation separation factor. Reprinted/adapted with permission from Ref. [373]. Copyright 2019, Elsevier.

Table 3 summarizes the current state-of-the-art hollow fiber membranes for solvent dehydration via pervaporation. Liu et al. demonstrated that a novel four-channel alumina hollow fiber support (Figure 13a) endowed the outer-surface-coated NaA zeolite membranes with notably high water flux of 12.8 kg/m^2^ h and a separation factor >10,000 for ethanol dehydration [356]. Following that, Cao et al. employed a continuous gel solution recirculation system to synthesize the NaA zeolite layer on the inner surface of the substrate (Figure 13b) [357]. This work accomplished the best performance for ethanol dehydration, with a flux and separation factor of 19.7 kg/m^2^ h and >80,000, respectively. Liu et al. proposed the fabrication of thin polyamide (PA) film on the TiO_2_ modified ceramic hollow fiber substrate (Figure 13c) via interfacial polymerization of 1,3-diaminopropane (DAPE) and trimesoyl chloride (TMC) for pervaporation dehydration of isopropanol [188]. The resultant membrane demonstrated a permeation flux of 6.44 kg/m^2^ h and a separation factor of >12,000 due to the ameliorated surface smoothness and cross-linking degree. Moreover, Liu et al. reported well-intergrown Universitetet i Oslo (UiO)-66 MOF membranes fabricated on yttria-stabilized zirconia (YSZ) hollow fiber substrate via solvothermal protocol (Figure 13d), establishing an exceptional separation factor of >45,000 and water flux up to 6.0 kg/m^2^ h for the dehydration of isobutanol, furfural, and tetrahydrofuran [369]. These MOFs-based hollow fiber membranes remained robust over 300 h of long-term exposure to harsh chemical environments.

Apart from that, research has been expanding to the modification of the pervaporation membranes. Zhang et al. adopted a facile surface cross-linking method between chitosan and TMC to construct a thin separation layer (e.g., 1414 nm) on ceramic hollow fiber membranes with revitalized pervaporation performance [363]. In another work, mixed matrix hollow fiber membranes had been developed by incorporating UiO-66-NH_2_ into cellulose triacetate (CTA), which attained a rise in permeation flux through enhanced water affinity [359]. On the other hand, Zhang et al. attempted the surface functionalization of zeolite membranes with 3-aminopropyltriethoxysilane (APTES) to induce greater hydrophilicity, acquiring vast improvement in the pervaporation separation factor (Figure 13e) [373]. There were also other mentionable works venturing into more complex pervaporation of ternary mixtures, such as water-ethanol-acetic acid [395] and water-isopropanol-epichlorohydrin [370].

**Table 3:** Current state-of-the-art hollow fiber membranes for solvent dehydration via pervaporation.

Membrane ^a^	Water-Organic Mixture	Feed Composition (wt%)	Operating Temperature (°C)	Water Flux (kg/m^2^ h)	Separation Factor	Reference
CS-PSS/ceramic ^b^	water-ethanol	10/90	70	0.495	904	[355]
NaA/alumina ^b^	water-ethanol	10/90	75	12.8	10,000	[356]
NaA/alumina ^b^	water-ethanol	10/90	75	19.7	>80,000	[357]
T-type zeolite/YSZ ^b^	water-ethanol	10/90	70	0.78	>90	[358]
CTA/UiO-66-NH_2_/Ultem ^c^	water-ethanol	15/85	50	2.667	152	[359]
PA/PES/silicon rubber ^d^	water-ethanol	15/85	50	7.5	60	[360]
PA/TEPA/PAN ^d^	water-ethanol	10/90	25	0.342	366	[361]
CS-PVA/PVDF ^b^	water-isopropanol	10/90	60	0.306	2140	[362]
CS-TMC/alumina ^b^	water-isopropanol	10/90	70	0.908	8993	[363]
CTA/Ultem ^b^	water-isopropanol	13.3/86.7	125	13.41	1332	[364]
MoS_2_-PEI/TiO_2_/ceramic ^b^	water-isopropanol	10/90	70	5.697	320	[365]
PA/Ultem/PDMS ^b^	water-isopropanol	15/85	50	2.65	246	[366]
Teflon/Ultem ^b^	water-isopropanol	5/95	125	4.625	383	[367]
T-type zeolite/YSZ ^b^	water-isopropanol	10/90	75	7.36	>10,000	[368]
PA/TiO_2_/alumina ^d^	water-isopropanol	10/90	60	6.44	>12,000	[188]
UiO-66/YSZ ^b^	water-isobutanol	5/95	50	4.81	>45,000	[369]
SA/TDI-GA/CTA-PAN ^d^	water-isobutanol	2/98	25	0.021	3229	[370]
CHA zeolite/YSZ ^b^	water-acetic acid	50/50	75	12	>10,000	[372]
DD3R/ceramic ^b^	water-acetic acid	70/30	95	0.58	800	[191]
DD3R-APTES/ceramic ^b^	water-acetic acid	10/90	75	0.23	1700	[373]
SA-TDI-GA/CTA-PAN ^b^	water-acetic acid	5/95	25	0.012	708	[370]
P84/EDA ^b^	water-acetone	15/85	50	1.8	53	[374]
PBI/PEI’ ^b^	water-ethyl acetate	2/98	60	0.82	2478	[375]
UiO-66/YSZ ^b^	water-furfural	5/95	50	5.95	>45,000	[369]
	water-tetrahydrofuran	5/95	70	4.06	>45,000	[369]
T-type zeolite/alumina ^b^	water-ethanol-acetic acid	9.3/83.8/6.9	75	2.25	1348	[395]
GO-PVA-TEOS/alumina ^b^	water-isopropanol-epichlorohydrin	20/30/50	30	0.09	4844	[396]

^a^ APTES: 3-aminopropyltriethoxysilane; CHA: chabazite; CS: chitosan; CTA: cellulose triacetate; DD3R: Decadodecasil 3R; EDA: ethylenediamine; GA: glutaraldehyde; GO: graphene oxide; MoS_2_: molybdenum disulfide; PA: polyamide; PAN: polyacrylonitrile; PBI: polybenzimidazole; PEI: polyethyleneimine; PEI’: polyetherimide; PDMS: polydimethylsiloxane; PES: polyethersulfone; PSS: poly(4-styrenesulfonic acid); PVA: poly(vinyl alcohol); PVDF: polyvinylidene fluoride; SA: sodium alginate; TDI: 2,4-toluene diisocyanate; TEOS: tetraethyl orthosilicate; TEPA: tetraethylenepentamine; TiO_2_: titanium dioxide; TMC: trimesoyl chloride; UiO: Universitetet i Oslo; YSZ: yttria-stabilized zirconia. ^b^ Dual-layer hollow fiber membrane made of selective layer/substrate. ^c^ Dual-layer mixed matrix hollow fiber membrane containing polymer/filler in the selective layer. ^d^ TFC hollow fiber membrane composed of selective layer/substrate/sealing layer or selective layer/gutter layer/substrate.

### 4.6. Gas and Vapor Separation

The yearning for clean air has urged the development of promising air purification and separation technology to address this global challenge. The emergence of the application of hollow fiber membranes in industrial practice can be witnessed since the first patented polymeric hollow fiber membranes five decades ago by Mahon [14]. To date, hollow fiber membrane has been vastly implemented in gas separation application, including CO_2_ capture to constrain the amount of greenhouse gases released into the atmosphere, air purification to obtain high-purity oxygen (O_2_) and nitrogen (N_2_), H_2_ separation for hydrogen energy systems, hydrocarbon separation via a cost- and energy-efficient approach, sulfur dioxide removal in flue gas stream prior post-combustion CO_2_ capture process, sour gas sweetening for natural gas purification, flue gas dehydration via vapor permeation as well as air filtration from ultrafine pollutants such as particulate matter and air dehumidification [12,397].

Organic polymers are extensively reported in fabricating hollow fiber membranes for gas and/or vapor separation in either single layer, dual-layer, thin film composite, thermally rearranged, or mixed matrix configuration. They are cheap, commercially available, scalable, possess defect-free nature, and have promising separation performance. Polymers such as PES [29,158,171,172,183,398,399,400,401,402,403,404,405,406], PSF [28,30,36,407,408,409,410,411,412,413,414,415,416,417,418,419], polyimide [110,420,421,422,423,424,425,426,427], PAN [34,70,162,170,428,429,430,431], and PBI [180,432,433] are the common polymers used to fabricate hollow fiber membranes in various configurations. Notably, PBI has been widely reported for effective H_2_ purification at elevated temperatures benefiting from its rigid polymer chains, high thermal properties, and diffusivity-based selectivity at a temperature above 150 °C [433]. In addition to the common application such as CO_2_ capture [30,34,66,70,110,158,162,170,171,172,399,400,407,408,409,420,422,423,424,425,429,431,434,435,436,437], air purification [29,398,401,402,403,410,411,421,426,438], and hydrogen separation [28,36,183,404,427,432,433,439,440], polymeric-based hollow fiber membranes also received attention to be used for industrial applications, e.g., hydrocarbon [425,441,442,443,444], sulfur dioxide [33,445,446], and sour gas [447] separation, water vapor permeation [405,406,412,413,414,415,416,417,418,419,428,430], as well as particulate matter removal [67,448]. Nevertheless, polymeric hollow fiber in integrally skinned configuration frequently suffered from physical aging and plasticization upon prolonged application due to their molecular orientation, thus restricting their diverse application [449,450,451]. To circumvent these drawbacks, proper tailoring on spinning parameters, the phase inversion process, and the chemical structure of polymer could simultaneously bring about significant enhancement to its separation performance as well as produce a hollow fiber with desirable separation performance with an ultrathin dense selective layer [66,89,452,453,454]. Alternatively, CO_2_ conditioning [449,455,456] can be used to control the physical aging rate, while strategies such as chemical cross-linking [400,457,458], thermal cross-linking [45,447,459,460,461], thermal annealing [442,462,463], and polymer blending [28,36,110] would be the promising approaches to suppress CO_2_-induced plasticization in polymeric hollow fiber membranes.

Electroconductive polymeric membranes show their potential to overcome the permeability-selectivity trade-off for membrane gas separation. The huge π-systems in conductive polymers provide stiff polymer chains with low packing density. Thus, they form a rigid polymer structure that offers more restriction toward the gas diffusion across the membrane [117], demonstrating the molecular size sieving properties of conductive polymer. Meanwhile, the organic group in a conductive polymer (e.g., -NH_2_) acts as the carrier to facilitate the transport of desired gas via a reversible chemical reaction with gas molecules [464], demonstrating its carrier-mediated transport properties. These polymers offered better selectivity toward polar gases owing to the combined properties of molecular size sieving and facilitated transport. Moreover, conductive polymers possess the ability to in situ tuning the gas transport properties of membranes via doping. Through proper tailoring of the doping, dedoping, and redoping process with oxidizing agents (e.g., hydrofluoric acid, hydrobromic acid, hydrochloric acid), precise control of the polymeric structure, membrane morphology, pore size, free volume, and the resultant gas permeation properties can be achieved [111,465,466]. Conductive polymer membranes were reported for H_2_ separation (e.g., H_2_/CO_2_, H_2_/N_2_), air purification, and CO_2_ capture (e.g., CO_2_/N_2_, CO_2_/CH_4_) in the form of self-supported membranes, composite membranes, and polymer blend membranes. Examples of conductive polymers that have been extensively reported for gas separation include PANI [111,467,468,469,470,471,472,473,474,475,476,477,478,479,480,481], polypyrrole [482,483,484,485,486,487,488,489], and polythiophene [490,491,492]. A comprehensive review of conductive polymer-based membranes for gas separation application was reported recently [117]. Noteworthy, Mattes’ group had conducted a few preliminary studies on conductive polymer fibers [493,494,495,496,497,498,499,500,501]. Nevertheless, research reported on conductive polymer-based hollow fiber membranes for gas separation is relatively limited, ascribed to the challenges such as low permeability, poor mechanical strength, poor solubility in common solvents, and easy agglomeration of fillers within the polymer matrix. 

**Figure 14:**
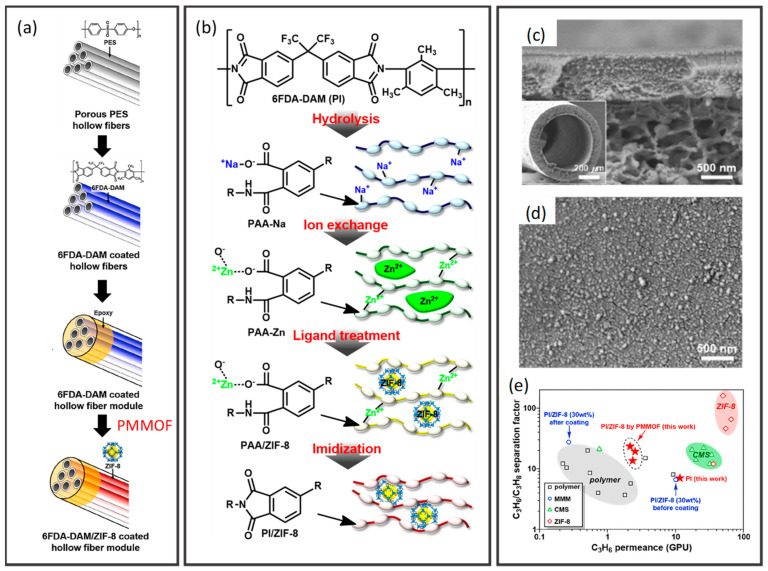
Schematics on (**a**) preparation of 6FDA-DAM/ZIF-8 coated hollow fiber module and (**b**) PMMOF process. SEM images of 6FDA-DAM/ZIF-8 hollow fiber membrane: (**c**) magnified cross-section and (**d**) top view. Inset image in (**c**) represent the overall cross-section of the membrane. (**e**) Comparison on C_3_H_6_ permeance and C_3_ separation factor of 6FDA-DAM/ZIF-8 hollow fiber modules with other works. Reprinted/adapted with permission from Ref. [503]. Copyright 2020, Elsevier.

Porous nanofillers such as ZIF-8 [197,502,503,504], ZIF-71 [430], Materials of Institute Lavoisier (MIL)-53 [505,506], MIL-125-NH_2_ [415], UiO-66-NH_2_ [507,508,509], GO [171,172], graphene nanoplatelets [91], MWCNTs [416], zeolite-templated carbon [510], Engelhard titanosilicate-4 (ETS-4) [183,417], carboxylated TiO_2_ (c-TiO_2_) [412,413], amino-functionalized acid activated bentonite clay (Abn-NH) [414], montmorillonite [181], and β-cyclodextrin (β-CD) [418,419] are recently reported to fabricate composite hollow fiber membrane for gas and vapor separation. They are incorporated into polymeric hollow fiber membranes either via direct spinning or during fabrication of thin film composite membranes. Exceptionally, ZIF-8, which possesses high thermal stability with a small pore aperture of 3.4 Å and large surface area of >1800 m^2^/g, have contributed to their wide application not only in CO_2_ capture [197] but also in air purification [502] and propylene (C_3_H_6_)/propane (C_3_H_8_) separation [503,504]. For instance, Etxeberria-Benavides et al. reported a PBI/ZIF-8 hollow fiber membrane for pre-combustion CO_2_ capture, where the fiber had a thin (e.g., ~300 nm) and defect-free selective layer that showed H_2_ permeance of 107 GPU with H_2_/CO_2_ selectivity of 16.1 [197]. Hadi et al. fabricated a PES/ZIF-8 hollow fiber membrane for air separation with a high O_2_/N_2_ selectivity of 5.25 [502]. Park et al. employed polymer-modification-enabled in situ metal-organic framework (PMMOF) strategy for in situ growth of ZIF-8 inside the 6FDA-(2,4,6-trimethyl-1,3-phenylene diamine) (6FDA-DAM) selective layer supported on PES substrate [503]. The PMMOF process involved hydrolysis, ion exchange, ligand treatment, and imidization (Figure 14). The membrane revealed promising C_3_H_6_/C_3_H_8_ separation with C_3_H_6_ permeance of 2.15 GPU and C_3_H_6_ selectivity of 23.4 with no additional defect sealing steps. Meanwhile, composite hollow fiber membranes with nanofillers of ZIF-71 [430], MIL-125-NH_2_ [415], MWCNTs [416], ETS-4 [417], c-TiO_2_ [412,413], Abn-NH [414], and β-CD [418,419] have showed promising results for water vapor permeation. Interestingly, Abn-NH incorporated hollow fiber membrane demonstrated superior water vapor permeance of 2809 GPU with water vapor/N_2_ selectivity of 913 [414].

Since the pioneering work on TR-polymers [511], TR-hollow fiber membranes have been vastly implemented for natural gas upgrading and carbon capture [512,513,514,515,516,517]. The thermal treatment temperature has a notable effect on the dense selective layer thickness and CO_2_ permeance of the resultant membrane, where the elevated temperature will increase the thickness of the dense selective layer due to the densification effect, thus reducing the CO_2_ permeance [514]. Instead of omni-directional shrinkage, Lee et al. found that the longitudinal-suppressed densification process produced a TR-hollow fiber membrane with a thinner selective layer and higher CO_2_ permeance [517]. Interestingly, by applying the 2D densification process, the cross-linkable hydroxyl polyimide hollow fiber membranes demonstrated a defect-free ultrathin selective layer of 52 nm and an exceptionally high CO_2_ permeance of 4600 GPU with CO_2_/N_2_ selectivity of 18 [517], while the fiber prepared by usual densification had a thicker selective layer of 100 nm with lower CO_2_ permeance of 2291 GPU [516]. Notwithstanding, they are yet to be explored in other gas separation such as H_2_ or hydrocarbon separation. Likewise, TR-derived mixed matrix membranes were only reported on flat sheet configuration for CO_2_ [518,519,520,521] and H_2_ [522,523,524,525] separation. Given the splendid properties, further exploitation of the newly developed TR-derived hollow fiber membranes is essential to realize its implementation in real-time application.

Meanwhile, inorganic hollow fiber membranes such as carbon-based and ceramic-based are extensively reported and focus on H_2_, O_2_, and CO_2_ separation. Recent works also include zeolites [526,527], alumina [528], and MXene [201] hollow fiber membranes for gas and vapor separation. CMS hollow fiber membranes are temperature- and pressure-resistant materials with a rigid pore structure that are suitable for natural gas sweetening and H_2_ purification processes in steam methane reforming plants. The bimodal pore structure (e.g., ultramicropores and micropores) in CMS membranes favor the CO_2_- [142,145,148,529,530,531,532] and H_2_-related [137,138,141,533] separation at high gas selectivity. CMS hollow fiber membranes have been employed for air purification [144,148], H_2_/alkane separation [138,141,533], and olefin/paraffin separation [140,148,534]. Table 4 summarizes the recently reported CMS hollow fiber membranes for these applications. Qiu et al. introduced a “hyperaging treatment” on the CMS hollow fiber membrane at a temperature range of 90–250 °C, which accelerated the aging, reduced ultramicropores structure, and enhanced selectivity [141]. The results revealed that the H_2_/C_2_H_4_ selectivity increased more than 10-fold compared to the pristine membrane. However, the CMS membranes still possessed relatively large ultramicropores to allow precise sieving of H_2_ and CO_2_. Based on the “aging treatment” strategy, they proposed a “super-hyperaging” process to recover the separation performance of an aged CMS fiber in C_3_H_6_/C_3_H_8_ separation [140]. The fibers treated at super-hyperaging temperature near 280 °C under air achieved C_3_H_6_ permeance of 25 GPU with C_3_H_6_/C_3_H_8_ selectivity of 10. Indeed, the porous layer of asymmetric precursors is prone to curl and collapse during thermal formation of CMS membrane, forming a denser layer that deteriorates the gas permeance [148,535]. Pre-treatment process such as chemical cross-linking or coating of a new layer is often required to “lock in” the asymmetric structure [304,536]. However, these modifications increased the fabrication complexity and the overall membrane production cost by up to 40% [148]. To circumvent this hurdle, Lei et al. immersed the water-wetted cellulose hollow fiber precursor in a glycerol solution before drying [529]. Compared to direct drying from water, this step effectively prevents the curling and pore collapse of cellulose hollow fiber. Nonetheless, the glycerol-containing cellulose precursor may contain large micropores as glycerol is a membrane pore radius-maintaining agent. They further proposed a drying pre-treatment method to remove glycerol and water inside the cellulose precursors [530]. Upon increasing the drying temperature, the crystallinity of cellulose hollow fiber precursors was enhanced, where substantial shrinkage resulted in narrow pore size (e.g., 4.9 Å) and improved selectivity. The resultant membrane demonstrated remarkable CO_2_/CH_4_ mixed gas selectivity of 131 when tested at 60 °C, 50 bar. Recently, the same research group employed non-solvent (e.g., water-isopropanol-*n*-hexane) exchange to dry the water-wetted cellulose membrane to prevent pore collapse during carbonization [137]. The resultant membrane, after optimized final carbonization temperature, exhibited superior H_2_ permeance of 148.2 GPU and an ideal H_2_/CO_2_ selectivity of 83.9, being the highest performance self-supported carbon membrane reported for H_2_ purification. The outstanding performance of CMS hollow fiber membrane demonstrated its potential for application in H_2_ and CO_2_ separation for real industrial application.

**Figure 15:**
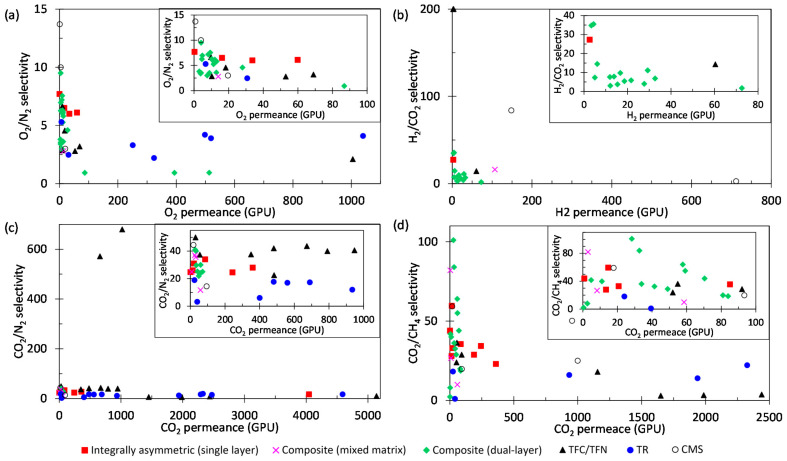
Performance plot on recent state-of-the-art hollow fiber membranes for (**a**) O_2_/N_2_, (**b**) H_2_/CO_2_, (**c**) CO_2_/N_2_, and (**d**) CO_2_/CH_4_. Inset is the magnified plot on the packed data point.

Figure 15 shows the performance plot on recent state-of-the-art integrally asymmetric, thermally rearranged, composite (e.g., dual-layer, TFC, TFN), and CMS hollow fiber membranes for O_2_/N_2_, H_2_/CO_2_, CO_2_/N_2_, and CO_2_/CH_4_ separation. For air purification, TR hollow fiber membranes showed the highest O_2_ permeance with moderate O_2_/N_2_ selectivity, while CMS hollow fiber membranes demonstrated a great permeability-selectivity trade-off with the highest O_2_/N_2_ selectivity but the lowest O_2_ permeability (Figure 15a). Despite the low O_2_ permeability, CMS hollow fiber membranes had the highest H_2_ permeance with comparable H_2_/CO_2_ selectivity, while TFC hollow fiber membranes displayed the highest H_2_/CO_2_ selectivity with the lowest H_2_ permeance (Figure 15b). Interestingly, TFC hollow fiber membranes dominated for CO_2_ permeance and CO_2_/N_2_ selectivity, approaching 5200 GPU and selectivity of 680 (Figure 15c), signifying the great potential of this membrane for flue gas separation. In addition, they also demonstrated the highest CO_2_ permeance for CO_2_/CH_4_ separation, while dual-layer hollow fiber membranes had the highest CO_2_/CH_4_ selectivity with low CO_2_ permeance (Figure 15d). Remarkably, each of these membranes has portrayed distinctive separation performance depending on the targeted applications.

The production of O_2_ from cryogenic distillation is limited by high capital and operating cost and is energy-intensive despite producing high-quality O_2_ (i.e., >99.9 mol%). Alternatively, high O_2_ purity up to 99.9 mol% at a lower cost in a compact module could be separated using ceramic membranes [537]. They are constructed from metal oxides with perovskite structures that contain a large number of O_2_ vacancies, which favor O_2_ transportation after activation at elevated temperatures [538]. An in-depth analysis from the chemistry and material side on perovskite oxides for O_2_ permeable membrane was reported recently [539]. However, the large-scale application of perovskite hollow fiber membranes is restricted by the inherent brittleness of the ceramic materials. In order to overcome the physical weakness of these membranes, perovskite hollow fiber membrane bundles were introduced [537,540]. By applying this strategy, the bending force of the resultant La_0.6_Sr_0.4_Co_0.2_Fe_0.8_O_3−δ_ (LSCF) bundle with five fibers increased 540% (e.g., from 2.81 N to 11.77 N), showing O_2_ flux of 0.46 mL cm^−2^ min^−1^ at 950 °C [540], while the BaCo_0.85_Bi_0.05_Zr_0.1_O_3−δ_ (BCBZ) hollow fiber bundle with seven fibers had a bending force of 25.32 N and achieved even higher O_2_ flux of 7.06 mL cm^−1^ min^−1^ at 1000 °C [537]. Wang et al. fabricated a 19-channel Ba_0.5_Sr_0.5_Co_0.8_Fe_0.2_O_3−δ_ (BSCF) hollow fiber membrane in which this configuration brought superior mechanical strength of 50.4 ± 1.3 N in 50 mm with enhanced O_2_ permeation of 7.05 mL cm^−2^ min^−1^ at 900 °C [541]. Other types of perovskite such as La_0.6_Sr_0.4_CoO_3−δ_ (LSC) [542] and (La_0.6_Ca_0.4_)(Co_0.8_Fe_0.2_)O_3−δ_ (LCCF) [543,544] hollow fiber membranes were also reported for O_2_ permeation. In addition, the formation of a hetero-interface between perovskite and Ruddlesden-Popper oxide in the membrane system was proved to enhance the surface O_2_ exchange kinetics due to the presence of fast and anisotropic O_2_ transport pathways along the hetero-interface [545,546]. This hetero-interface can be formed via surface coating on pristine hollow fiber [546,547,548] or by forming a composite membrane via a combined phase-inversion-sintering process [549]. Compared to surface decoration [548], the O_2_ permeation flux on LSCF-LSC composite hollow fiber membrane showed a superior enhancement of 550% (e.g., 0.6 to 3.3 mL cm^−2^ min^−1^) at 900 °C [549], indicating that the O_2_ bulk diffusion was significantly promoted by the hetero-interface. Apart from that, incorporation of Ag on perovskite membrane can also improve the O_2_ fluxes [127,550]. Ag has good O_2_ solubility and compatible catalytic activity that promotes O_2_ permeation [550]. For instance, Han et al. [127] deposited Ag on LSCF hollow fiber membrane via dip coating method, while Ma et al. [550] incorporated Ag into La_0.8_Ca_0.2_Fe_0.94_O_3−a_ (LCF) hollow fiber membrane via one-pot wet complexation method. The O_2_ flux in the former work was enhanced by 240%, and the latter was 160%. Notwithstanding, particle aggregation happened easily during high-temperature operation, which reduced the catalytic efficiency.

Flue gas contains nitric oxide (NO) that imposes a negative effect on the O_2_ permeation of perovskite membranes in the temperature range of 700–900 °C, in which the reaction between perovskites and NO will damage the porous structure of the membrane. To address the said issue, Gao et al. elucidated the impact of NO on the O_2_ permeation behavior of perovskite hollow fiber membrane and found out that operation above 900 °C was able to omit the negative effect as NO was decomposed at such elevated temperature [551]. Xu et al. fabricated a molten nitrate dual-phase four-channel potassium nitrate/potassium nitrite-BSCF (KNO_3_/KNO_2_-BSCF) hollow fiber membrane for nitrogen oxides separation where the nitrogen oxides permeance reached 9.2 × 10^−9^ mol m^−2^ s^−1^ Pa^−1^ at 500 °C [129]. Moreover, the sealing of perovskite membrane at a high temperature of 800 °C remains a huge challenge for industry feasibility. The choice of sealants varies between membrane materials, operating conditions, and duration. A comprehensive discussion on sealing perovskite hollow fiber membranes for long-term O_2_ permeation has been reported elsewhere [552]. Modeling and simulation of O_2_ permeation have also been performed to evaluate the performance of the perovskite hollow fiber membrane module at different modes and flow configurations [553,554]. Table 5 summarizes the recent publication on perovskite hollow fiber membrane for O_2_ separation.

### 4.7. Membrane Distillation (MD)

Membrane distillation (MD) is a vapor-driven thermal separation process with a porous hydrophobic membrane as the separation medium [556]. It has been often used in removing various pollutants such as dyes, salts, and heavy metals from textile wastewater [557,558,559], seawater [560,561,562], mining effluent [563,564], and water production [565,566]. Additionally, membrane distillation has been used for alcohol/water separation [567,568]. As compared to conventional distillation, a differential distillation utilizes hollow fibers as structural packing for olefin/paraffin separation [37]. In this case, the hollow fibers represent the tubes in a shell-and-tube heat exchanger for mass transfer in membrane technology. Among these separations, MD with hollow fiber membrane configuration is attractive for dye wastewater treatment [95] and desalination process [71,101,104,195,569,570,571,572,573,574].

As compared to inorganic membranes, polymeric membranes are often selected in various studies due to their lower cost and greater mechanical flexibility [575]. For instance, macrovoids-inhibited PVDF membrane synthesized via spinning process delay for direct contact membrane distillation (DCMD) enhanced the porosity, pore size, hydrophilicity, and mechanical strength as compared to the conventional NIPS method [101]. This was due to the elimination of macrovoids, enhancing the water flux and energy efficiency without requiring any additional chemicals. In another study, Chang et al. synthesized PVDF hollow fiber membranes using triethyl phosphate (TEP) as a green solvent for DCMD [569]. The resultant membranes prepared using TEP show their potential in replacing the commercial PVDF membrane with NMP owing to less toxicity of solvent used toward the environment, robust mechanical strength, simple fabrication method, anti-wetting property, high NaCl rejection of 99% and flux of 20 kg/m^2^·h.

In addition, Chen et al. synthesized a robust Janus tri-bore hollow fiber membrane consisting of three layers, which included a porous and hydrophobic tri-bore PVDF hollow fiber substrate, an oxidant-induced hydrophilic polydopamine (PDA)/PEI layer, and a highly hydrophilic sodium-functionalized carbon quantum dots (Na^+^-CQD) grafting layer [104]. The hydrophilic Na^+^-CQD grafted layer provided excellent wetting and fouling resistance, resulting in robust desalination performance in wastewater with multiple components such as inorganic salts, organic compounds, surfactants, and crude oil. Unlike the previous study, Li et al. synthesized a hydrophobic ZIF-71/PVDF hollow fiber composite membrane via a dilute solution coating method [570]. The hydrophobic membrane inhibited the formation of liquid film and reduced the thickness of temperature polarization, resulting in an enhancement of permeation and wetting resistance. 

Other than polymeric membranes, inorganic ceramic hollow fiber membranes are prepared. For instance, Hubadillah et al. synthesized a green silica-based ceramic hollow fiber membrane for desalination derived from rice husk ash waste [560]. The developed membrane is able to withstand harsh conditions in the real industrial process with feed temperatures as high as 80 °C while maintaining the permeate temperature at 10 °C and liquid entry pressure above 0.5 bar. The result showed a higher permeate flux (e.g., 27.3–64.3 kg/m^2^·h) compared to those of polymeric membranes (e.g., 5–25 kg/m^2^·h) [576] and β-Sialon ceramic membrane (e.g., 12.2 kg/m^2^·h) [577].

### 4.8. Membrane Contactor

**Figure 16:**
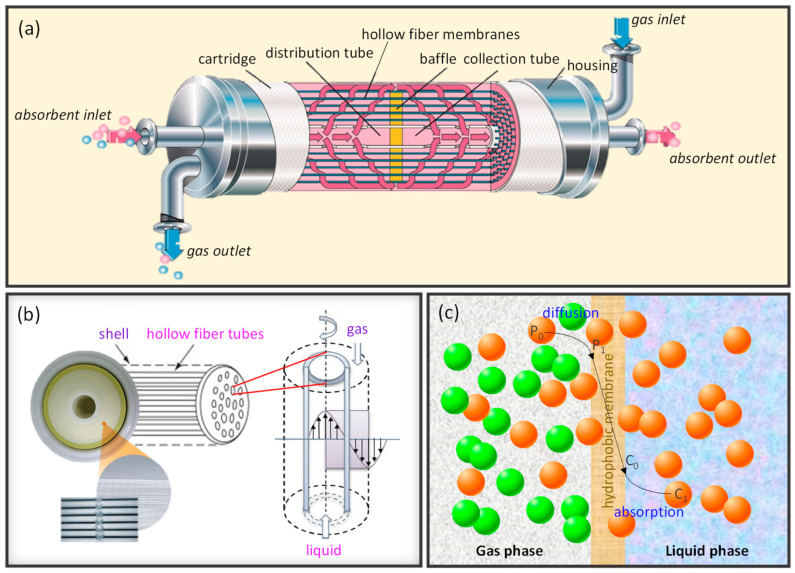
(**a**) Shell-and-tube module of a membrane contactor. (**b**) Hollow fiber configuration where the tube side is absorbent flow. (**c**) The diffusion and absorption mechanisms in the membrane contactor.

Membrane contactor technology combines the principles of membrane separation and absorption. Generally, the membrane acts as a non-selective barrier between the gas-liquid interface for efficient contact without phase dispersion. This feature is notably beneficial since the phases are readily separated in output without droplet carry-out or foaming in the liquid phase; therefore, gas and liquid flow parameters can be controlled independently [578]. The development of hollow fiber membrane contactors was inspired by the conventional shell-and-tube modules (Figure 16a), with hollow fibers acting as tubes to seize their benefits of high packing density and exceptional specific surface area (Figure 16b). In this regard, the design of hollow fiber membrane contactors had been well established using mass transfer correlations for aqueous deaeration (i.e., removal of oxygen from liquid phase) and CO_2_ absorption [579]. The contacting phases can be introduced in a co-current or counter-current flow regime [580]. Membrane contactors are mainly employed to absorb the desired component in a liquid absorbent, as illustrated in Figure 16c. On the shell side, the gas molecules diffuse through the pores of the membrane to the gas-liquid interface through the partial pressure gradient and concentration gradient. They react with the absorbent in the tube side, whereby the desired component is absorbed and removed.

Microporous polymeric membranes prepared from PES [581], poly(4-methyl-1-pentene) (PMP) [582], PVDF [583], polytetrafluoroethylene (PTFE) [584], and polydimethylsiloxane (PDMS) [585] have been used in membrane contactors. Despite being non-selective, the membrane morphology and transport properties affect the overall separation performance of a membrane contactor. For instance, increasing the membrane thickness prolongs the gas residence time and its resistance across the path, therefore reducing its separation efficiency. Previous analysis also showed that the absorption performance was improved by increasing the number of hollow fibers, fiber effective length, and porosity, as well as reducing the inner diameter and tortuosity factor of the hollow fiber membranes [586]. Another significant parameter is the anti-wetting property of the membrane, whereby an increase of 5–10% wetting reduced the separation efficiency by at least 40% [587,588,589]. In addition, the membrane contactor can be innovated through different operation modes such as serial [590] and counter-current flow operation [591].

Membrane contactors have major applications in acid gas (e.g., CO_2_, SO_2_, and H_2_S) removal [585,586,592,593,594,595,596,597,598,599,600,601,602]. The separation performance of membrane contactors mainly relies on the selective absorption of absorbents. Correspondingly, amine solutions, specifically ethanolamine derivatives, are one of the most competitive absorbents in acid gas removal [587,589,594,597]. However, they are hindered by high corrosion, expensive regeneration, and loss in absorbents due to volatilization and poor thermal degradation. These limitations lead to the progress of alternative absorbents like amino acid salt solutions [603], nanofluids [604,605], and ionic liquids [588,599,600,601], which exhibit ameliorated absorption capacity, stability, and regeneration efficiency. Apart from that, recent engineering approaches to structural steric effect [606] and blending [594,607] of amine absorbents have been reported. Other applications of membrane contactors include oxygenation/deoxygenation [608,609], ozonation [610,611,612], gas dehumidification [613,614,615,616,617], and olefin/paraffin separation [618,619].

### 4.9. Other

Improper treatment of effluent wastes from nuclear processing causes environmental pollution, including air and water pollution as well as affects human health [620]. Krypton and xenon are examples of radioactive wastes from nuclear fuel production that require effective management. In this respect, krypton/xenon separation is an important treatment as krypton and xenon hold great promise in a wide range of applications such as anesthesia, imaging, and lighting product [621]. However, the separation of krypton from xenon remains challenging due to the similar physical properties of these two chemical inert isotopes. In 1980, silicon rubber capillaries (hollow fiber) were introduced to investigate the permeation of krypton [622,623]. Several advantages of this module had been concluded in this study, including large effective area, high mechanical strength, and small module size, resulting in low module cost. Recently, Kwon et al. successfully demonstrated silicoaluminophosphate chabazite (CHA) (SAPO-34) membranes on α-alumina hollow fibers for krypton/xenon separation [624]. This membrane showed excellent krypton/xenon permeation selectivity. Meanwhile, Wang et al. fabricated a high-silica CHA zeolite membrane to capture krypton over xenon selectively [625]. As a result, krypton/xenon selectivity was up to 146 [625], which outperformed the hollow fiber SAPO-34 membranes, which was only 31.7 [624]. Moreover, the fabricated membrane also showed an outstanding radioactive resistance to more than 100 kGy of radiation.

**Figure 17:**
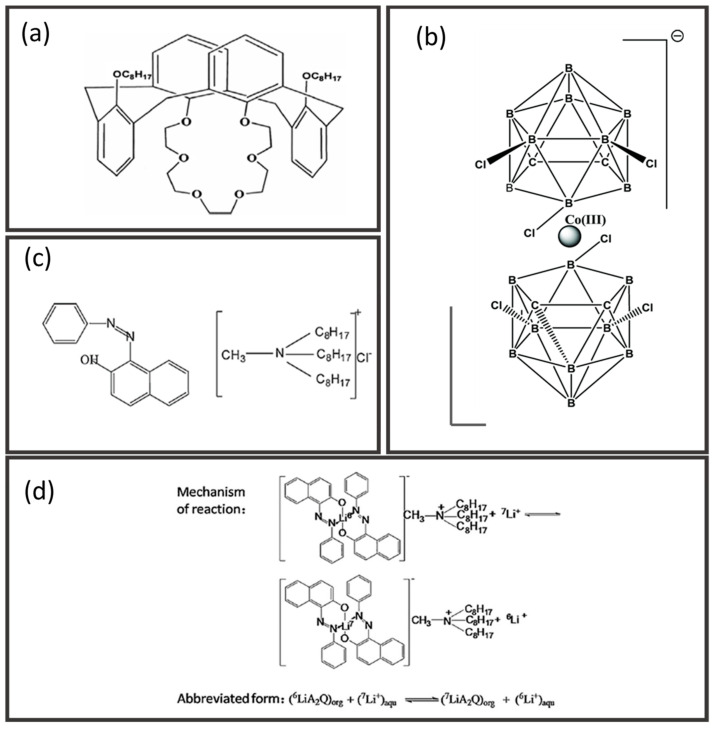
Chemical structure of synergetic ligands: (**a**) bis−octyloxy−calix [4]arene−mono−crown−6, (**b**) acidic chlorinated cobalt dicarbollide, (**c**) 1−phenylazo−2−naphthol and methyl trioctyl ammonium chloride, and (**d**) lithium isotope and ligands exchange mechanism. Reprinted/adapted with permission from Refs. [627,628,629]. Copyright 2014, 2016, and 2020, Elsevier.

In addition, hollow fiber-supported liquid membranes have been employed for nuclear waste treatment [626]. Various synergistic ligands (Figure 17a–c) have been used as carriers for effective ion transport in nuclear waste. Chaudhury et al. demonstrated electrodriven transport of radio-cesium through acidified chlorinated cobalt dicarbollide-loaded hollow fiber membrane for recovery of radio-cesium from nuclear waste [627]. Likewise, Jagasia et al. incorporated calix-crown-6 ligands (bis-octyloxy-calix [4]arene-mono-crown-6) into polypropylene hollow fiber, where the calix-crown-6 ligands carrier effectively enhanced the permeation of cesium through the membrane [628]. In another work, Song et al. synthesized poly(ethylene vinyl) alcohol (EVAL) hollow fiber membrane with extractant from a mixture of 1-phenylazo-2-naphthol and methyl trioctyl ammonium chloride for lithium isotopes separation [629]. Excellent lithium ions transport was attributed to the strong oxygen-lithium and nitrogen-lithium conjugations (Figure 17d).

**Figure 18:**
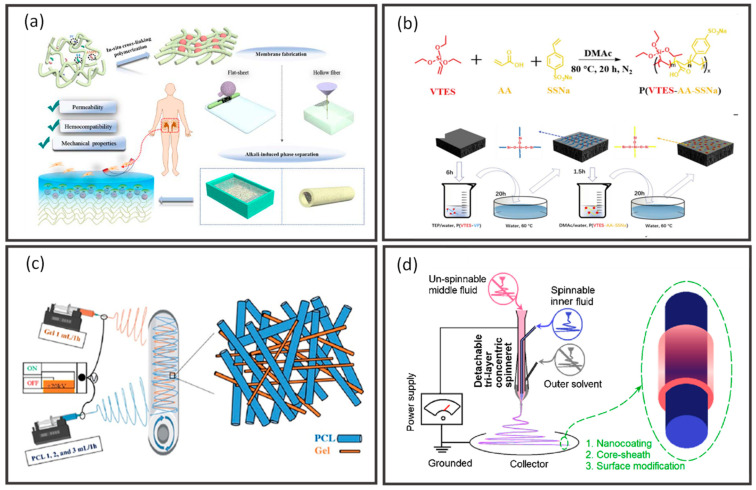
(**a**) Demonstration of NaOH-induced phase separation for the heparin-mimicking modified hollow fiber membrane. Reprinted/adapted with permission from Ref. [632]. Copyright 2021, Elsevier. (**b**) Synthesis procedure of anionic P(VTES-AA-SSNa) for free cross-linking process. Reprinted/adapted with permission from Ref. [633]. Copyright 2018, Elsevier. (**c**) Co-electrospinning technique for PCL/gelatine membrane preparation. Reprinted/adapted with permission from Ref. [636]. Copyright 2021, Elsevier. (**d**) Trixial electrospinning process for nanofiber with release-retarding coating. Reprinted/adapted with permission from Ref. [637]. Copyright 2019, Elsevier.

Hollow fiber membranes are widely applied in the biomedical field, such as hemodialysis, drug delivery and blood separation. An increasing number of publications on hollow fiber dialyzers has been reported for maintaining homeostasis of patients who suffer from chronic kidney failure by removing the uremic toxin and excess water from their body [630,631]. Wang et al. successfully fabricated PES hollow fiber membrane with a heparin-mimicking surface through in situ cross-linking polymerization mediated alkali-induced phase separation (Figure 18a) [632]. In another study, Wang et al. demonstrated heparin-free cross-linking on hollow fiber to enhance the hemocompatibility of dialysis membranes. The heparin-mimicking pre-copolymer, poly (triethoxyvinylsilane-acrylic acid-sodium 4-vinylbenzenssulfonate) (P(VTES-AA-SSNa)) was synthesized (Figure 18b) and subjected to cross-linking of PSF commercial hollow fiber [633]. Both studies revealed that heparin cross-linking on hollow fiber membranes could suppress platelet adhesion and prevent protein fouling from achieving a prolonged clotting time [632,633]. Moreover, functionalized GO-PEI hollow fiber membrane fabricated by Kaleekkal et al. possessed excellent toxins rejection (e.g., 77% urea, 68% creatinine, and 44% lysozyme) and good protein retention, about 95% after 4 h dialysis [630].

Nanofiber has been used as a drug delivery system to sustain drug release and avoid the uncontrollable initial burst of release [634]. Electrospinning, a common method for nanofiber fabrication, has been modified to improve biocompatibility and working efficacy [635]. Al-Baadani et al. demonstrated a co-electrospinning technique to synthesize polycaprolactone (PCL)/gelatine blended nanofiber membrane [636]. Through tuning the PCL and gelatine ratio, the drug release profile was adjusted (Figure 18c). In another study, a novel triaxial electrospinning method was employed to synthesize highly efficient cellulose acetate coated nanofiber by using cellulose acetate as a middle solution and ferulic acid/gliadin as a core solution (Figure 18d) [637]. Results revealed that cellulose acetate coating inhibited the initial burst of drugs, while proper tuning of the thickness of cellulose acetate coating achieved a constant rate of drug release profile for a sustained period.

Conductive polymers have been used in hollow fiber membrane modules for blood separation and sensing applications. Wu et al. are the frontiers of integrating the conductive nanomaterials on hollow fiber for blood separation. Firstly, a novel 3D electrochemical biosensor was designed via in situ synthesizing PANI and platinum nanoparticles on PSF hollow fiber membrane [638]. The unique structures of hollow fiber facilitated the liquid transportation and uniform diffusion in lumen and provided selective blood separation for metabolite detection. The resultant membrane exhibited a fast fluid flow (e.g., >1 μL/ms), good selectivity, high sensitivity (e.g., R^2^ = 0.999), and high accuracy (testing bias < 7.2%) for the separation of glucose and cholesterol from whole blood. In another work, Wu et al. incorporated enzyme-Fe-UiO-66 into conductive microporous hollow fiber membrane to develop a biosensor for various kinds of metabolite detection in blood [639]. The nanostructured conducive network developed from the conductive material and MOF-enzyme improved the electrochemical property, displaying a fast and selective metabolite detection. This membrane holds great potential in replacing conventional blood detection, which requires long sample preparation time and complicated procedures.

## 5. Emerging R&D on Hollow Fiber Membranes

### 5.1. Green Fabrication Technique

#### 5.1.1. Green Solvent

Membrane fabrication produces nearly 50 billion liters of solvent-contaminated wastewater annually [640]. These solvents are often toxic that cause severe human health threats and environmental burdens. For instance, toxic solvents such as DMF, NMP, and dimethylacetamide (DMAc) have been restricted to industrial use by European Chemical Agency (ECHA) for human health reasons [641]. Thereafter, less hazardous solvents emerged as a promising alternative to replace the toxic solvents for membrane fabrication. Recently, green solvents such as PolarClean [83,642], TEP [569,643], DMSO, γ-butyrolactone (GBL) [644,645], and natural deep eutectic solvent (NADES) [646] have been reported to fabricate green hollow fiber membranes. Rhodiasolv^®^ PolarClean [647] is a new frontier of green solvent that is water-soluble and provides good solvent/non-solvent exchange during phase inversion. For the first time, PolarClean was used as a green solvent to fabricate PVDF hollow fiber membranes by a combination of TIPS and NIPS techniques [648]. Hassankiadeh et al. reported the use of PolarClean as solvent and glycerol as additives; it induced porous structure and created B-polymorphs (unique polymorphism of PVDF, which can improve the filtration) in the polymers matrix, resulting in highly pure water permeability. In another work, Ursino et al. used PolarClean as a solvent to fabricate PES hollow fiber membrane [649]. The resultant membrane had a smooth and homogeneous surface with a sponge-like structure. In addition, tributyl O-acetyl citrate was used as a green diluent to fabricate hollow fiber membranes [25]. Results revealed that increasing the air gap and bore fluid temperature improved the porosity, resulting in higher water permeability. DMSO was also used to synthesize ultrafiltration membranes for water separation [650]. It was reported that the replacement of DMF with DMSO showed significant improvement in the water flux from 158 to 180 LMH/bar and salt rejection from 82 to 89%. A recent work used deep eutectic solvents (DESs) and NADES as solvents to prepare PVDF and PAN membranes for water application [646]. Co-solvents such as PolarClean, TEP, and DMSO were added to the dope solution with the aim of reducing the solution viscosity. It would be a great evolving step to fabricate a hollow fiber membrane based on this strategy. These works have demonstrated the great potential of using green solvents in preparing more sustainable membranes for vast application.

#### 5.1.2. Solvent-Free Method

**Figure 19:**
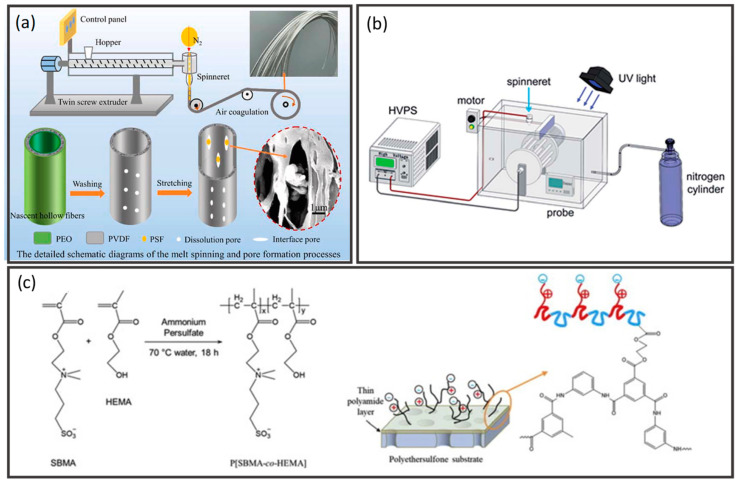
Schematic diagram of (**a**) melt-spinning technique with proposed stretching mechanism [656] and (**b**) UV light-assisted electrospinning method. Reprinted/adapted with permission from Refs. [656,657]. Copyright 2016 and 2021, Elsevier and Royal Society of Chemistry. (**c**) Copolymer synthesis via free radical polymerization with the proposed grafting mechanism where copolymer reacts with residual acyl chloride groups on the polyamide surface. Reprinted/adapted with permission from Ref. [664]. Copyright 2019, Elsevier.

Green fabrication can be achieved by involving less solvent and energy usage and safe and simple procedures. The techniques that satisfied said characteristics are MS-S [651,652] and ultraviolet (UV)-assisted melt electrospinning [653]. In the melt spinning process, a mixture of polymers is melted and spun by stretching force in a twin-screw extruder (Figure 19a). Poor compatibility between the two blended polymers will result in a thin interface layer that favors pore formation. The low adherent strength between matrix and dispersed phase eases the formation of interface pores during stretching, thus improving the porosity and permeability of membranes. Pore-forming agent can be introduced to enhance further the permeability of membranes [654,655]. Ji et al. used the MS-S technique to fabricate hollow fiber membranes for wastewater treatment [655]. PVDF, PTFE, and polyethylene oxide (PEO) were selected as matrix phase, dispersed phase, and pore-forming agent, respectively. The fabricated membrane showed a high dye rejection at about 93%. This study also concluded that PEO can be recycled and regenerated as a pore-forming agent, achieving a sustainable membrane fabrication. In the following work, they replaced PTFE with PSF as a dispersed phase due to its poorer compatibility with PVDF, which increased the porosity of the membrane [656]. The resultant membrane showed that 150% stretching increased the water flux from 128.0 to 1463.7 LMH/bar with about 100% of salt rejection.

Electrospinning is a promising candidate for hollow fiber membrane fabrication as it obtains nanofiber with a large surface area and high porosity. He et al. proposed a novel UV light radiation-assisted solvent-free electrospinning technique to prepare nanofibrous for air filtration (Figure 19b) [657]. DR-U301 polyurethane acrylate, which can be easily cured by UV light, was used as the monomer for electrospinning. The experiment proposed that oxygen atoms in the air will inactivate the free radicals generated from photoinitiators, which suppressed the initiation of polymerization. In addition, a needleless melt electrospinning method was employed to fabricate oriented fiber polypropylene membranes for microfiltration [658]. The resultant membrane showed a high rejection for 0.5 μm particles without compromising the water permeate flux. Markova et al. prepared a PMP-based hollow fiber membrane via melt-electrospinning for gas separation [659]. The resultant membrane in the triaxial braided hose showed CO_2_ permeability in a range of 40–97.9 Barrer and CO_2_/CH_4_ selectivity of 4.7–10. The solvent-free method avoids the challenges related to the removal of solvent and solvent recovery, ensuring a greener and safer fabrication method compared to the traditional method.

### 5.2. Modification of Hollow Fiber Membranes via Greener Approach

Green solvents are used as additives during the fabrication of hollow fiber membranes to enhance separation performance. There is a wide range of functional groups in the green solvent, which can influence the final morphologies, structures, and gas transport properties of the resultant hollow fiber membranes [660]. Fam et al. deposited ionic liquids (ILs) onto the surface of hollow fiber by dip coating [661]. The dense selective ILs layer provided adsorption sites for CO_2_, enhancing the CO_2_ affinity and solubility of the membrane. Yihdego et al. utilized ILs to modify nanocomposite PEI hollow fibers for dehydration [662]. ILs were incorporated into carbon capsules and suspended in polydimethylsiloxane, followed by dip coated on the hollow fiber support. The resultant membrane demonstrated water vapor permeance of 10,000 GPU and water vapor/N_2_ selectivity of 4500. Zhao et al. utilized the green cross-linking method to fabricate cross-linked PBI hollow fiber by immersing the support in a K_2_S_2_O_8_/water solution for OSN [108]. The cross-linked membrane showed a high rejection of rose bengel from acetone at about 99%. In another work, Li et al. demonstrated green plasma-flow-induced graft polymerization for hollow fiber cross-linking [663]. The H_2_O plasma-induced membrane resulting from surface hydroxylation showed an enhancement in the membrane hydrophilicity with promising water flux. In addition, Zhang et al. synthesized zwitterionic copolymers via free radical polymerization in water, followed by in situ grafting of copolymers on the inner surface of hollow fiber for RO application (Figure 19c) [664]. The resultant antifouling membrane obtained a water permeability of up to 10 LMH/bar and NaCl rejection of 98%. These green-modified hollow fiber membranes demonstrated promising separation performance similar to the conventional method. 

### 5.3. Green Materials for Hollow Fiber Membranes Fabrication

**Figure 20:**
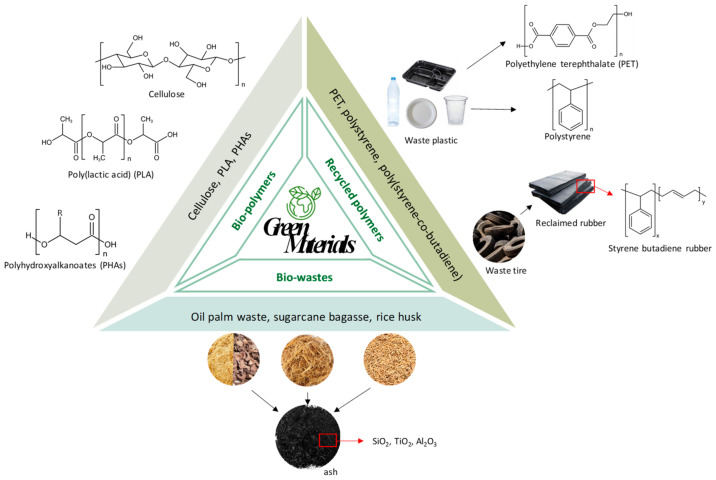
Sustainable materials used to fabricate green hollow fiber membranes.

While solvent selection plays an important role in membrane fabrication, the choice of materials could be another indispensable part of achieving a green and sustainable goal. Figure 20 illustrates the green materials that can be used to fabricate hollow fiber membranes. Conventional synthetic engineering polymers such as PES and PSF possess good stability and processability that show excellent performance in membrane separation. However, the growing awareness of the impact of plastic discharge has initiated the scientific communities to exploit renewable alternatives for membrane fabrication. To date, there are many renewable polymers available, for instance, biopolymers, bacteria-produced polymers, and chemically synthesized polymers produced from renewable monomers [665,666,667].

Cellulose is one of the most abundant biopolymers found in nature. The application of cellulose as membrane materials started in the 19th century when Sidney Loeb and Sirivasan Sourirajan first used cellulose acetate to develop a RO membrane [668]. Since then, diverse applications of cellulose, cellulose derivatives, and chitosan as membrane materials have been witnessed in the past few decades. Notably, cellulose-based hollow fiber membranes have been vastly reported in applications such as seawater desalination [669], arsenic removal from water [670,671,672], OSN [673], CO_2_ capture [508,509,529,531,674], and natural gas sweetening [675]. Polylactic acid (PLA) and polyhydroxyalkanoates (PHAs) are other types of renewable biopolymers prepared from bacterial fermentation using bio-sourced monomers (e.g., starch, whey, etc.) [667]. These biopolymers are biodegradable in that they degrade at a faster rate compared to those polymers produced from oil [676]. For the past ten years (2012–2022), there have been over 5000 papers reported on using PLA for various applications based on the statistics obtained from SCOPUS. Despite the greener approach, there is only a handful of work that adopted PLA in fabricating or modifying hollow fiber membranes for water application [676,677,678], owing to the poor mechanical properties that restricted their application in the pressure-driven process [679]. Further studies are crucial to push the feasibility of these materials in membrane development for environmental benign.

Over the years, polymer recycling has been a hot topic to remediate the impact of plastic on the environment. Millions of single-use polyethylene terephthalate (PET) packaging have been disposed of daily, creating serious environmental problems due to their non-biodegradability. Efforts to recycle used PET bottles can be found in making automotive parts, furniture, carpets, soft furnishings, etc. The outstanding chemical and mechanical properties of PET materials have created the opportunity to turn these wastes into high-value products. This idea was brought out by Rajesh and Murthy, who turned the waste PET bottles into ultrafiltration membranes prepared via the phase inversion technique [680]. Additives such as PEG were added to enhance the membrane characteristic such as hydrophilicity, flexibility, and porosity. Kusumocahyo et al. further developed a lower-MWCO ultrafiltration membrane with improved permeate flux and rejection [681,682]. In moving forward, recycled PET was used to prepare membranes for oil-water separation [683,684], NF [685], and air NF [686]. To maximize their industrial implementation, scaling up into hollow fiber configuration is crucial for long-term sustainable development. In addition, polystyrene [687] and poly(styrene-co-butadiene) [688,689] waste obtained from post-consumer containers and tires, respectively, can also be recycled as membrane materials for gas separation. Notably, Lai et al. utilized tire waste as a hollow fiber substrate to prepare supported ionic liquid membranes (SILMs) via dip coating method [690]. With the optimum spinning condition, the smooth surface on the substrate enhanced the wetting stability, thus promoting the formation of a defect-free hollow fiber membrane for promising CO_2_ capture. These works demonstrated the great potential of material transformation from waste to high-value products using green engineering and substantially contributed to the circular economy future. 

Moreover, the high fabrication cost of ceramic membranes has actuated researchers to explore alternative low-cost raw materials to produce these membranes. Bio-waste such as rice husk, sugarcane bagasse, and oil palm waste were produced from bunches, husk, or straw and disposed into landfills or burned openly. Valorization of bio-waste into useful materials will be an effective way to reduce the environmental burden. As promising bio-waste materials that consist of precious oxides such as silicon dioxide (SiO_2_), aluminum oxide (Al_2_O_3_), and TiO_2_, they are recently reported to be used for the derivation of green ceramic hollow fiber membrane via phase inversion and sintering technique. For instance, Hubadillah et al. successfully prepared hollow fiber membranes from crystalline rice husk burned at 1000 °C and sintered at 1200 °C for seawater desalination via direct contact membrane distillation [560], Jamalludin et al. used waste sugarcane bagasse ash calcinated at 800 °C to fabricate ceramic hollow fiber membranes for water filtration [691], while Tai et al. developed palm oil fuel ash-derived ceramic hollow fiber membrane for oil-water separation [692]. It is interesting to note that a low sintering temperature is required to produce these green ceramic hollow fiber membranes, thereby further reducing the production cost. These studies opened a new avenue of green materials to fabricate hollow fiber membranes using renewable or bio-based materials. Progressive scientific and technical studies are pending to be investigated to apply these materials in diverse applications.

## 6. Conclusions and Perspectives

This comprehensive review synopsizes the fundamental principles of hollow fiber membranes, including the structural analyses, gas transport theory, and phase inversion mechanism of these membranes. Additionally, the state-of-the-art hollow fiber membranes in various fields such as liquid, gas and vapor separation, pervaporation, membrane distillation, and membrane contactor have been thoroughly discussed. Emphasis has been given to the scientific advancements in hollow fiber membranes over the last 5 years, providing up-to-date insights into this technology. Though witnessing the splendid implementation of hollow fiber membranes in the separation industries for the past few decades, continuation on striking more feasible and reliable membranes to address the real-time industrial application is indeed essential. Therefore, the following perspectives were outlined for future research and studies.

(1) The introduction of nanoparticles during the fabrication of hollow fiber membranes is a promising approach to circumvent the trade-off of water permeability-rejection or gas permeability-selectivity. Proper tailoring of the microporous properties during membrane fabrication or post-modification on the nanoparticles is essential to take full advantage of these materials in contributing to the structural properties as well as separation performance of resultant membranes in targeted industries. Comprehensive studies are also needed to tackle the frequently-faced challenges when nanoparticles are introduced in a membrane system, for instance, particle dispersibility, optimum loading, and interfacial morphology between phases.

(2) While demonstrating impressive implementation in many applications, only a handful of studies were found using hollow fiber membranes for OSFO despite being driven by natural osmotic pressure that requires less amount of energy than those pressure-driven separation processes. A recent study highlighted the viability of hollow fiber membrane for OSFO application in the pharmaceutical industry; hence, continuous efforts are needed to exploit its potential in this sector.

(3) Cross-linking and thermal annealing are effective in suppressing CO_2_-induced plasticization, especially in polymeric hollow fiber membranes. Nevertheless, the underlying mechanism of suppressing plasticization still requires intensive investigation focusing on the fundamental aspects. Likewise, understanding the occurrence of structural relaxation is vital to predicting aging behavior, thus developing an ideal membrane for long-term applications.

(4) Novel strategies to improve the chemical, mechanical, and thermal properties of hollow fiber membranes are needed to realize their feasibility in the real industrial environment. The next-decades hollow fiber membranes shall be robust and practical to separate flue gas streams containing hazardous and humid gas operated at high pressure, substituting the energy-intensive cryogenic distillation and pressure swing adsorption.

(5) Green substitutes provide an evolutionary step toward achieving sustainable goals. Despite the greener approach, these non-toxic solvents are expensive for membrane manufacturing due to the absence of large-scale production. In order to realize green remediation, it is crucial to investigate the membrane fabrication process from raw materials until the final stage of post-modification. Bio-waste derived materials, polymer and solvent recycling, cost analysis, and life cycle assessment should be considered for environmental benignity while achieving a circular economy.

In a nutshell, this work highlights the diverse application of hollow fiber membranes to achieve outstanding separation performance. Undeniably, novel two-dimensional and three-dimensional membrane materials with excellent pore structure, for instance, graphene, carbon nanotubes, MOFs, and COFs, should be studied to accelerate the development of high-performance hollow fiber membranes for various separation applications. Lastly, initiatives should be taken to design facile and scalable fabrication procedures, along with the use of low-cost and sustainable materials to broaden the applications of these membranes.

## Figures and Tables

**Table 1:** Separation performances of hollow fiber membranes in NF.

Membrane ^a^	Pressure	Permeance	Rejection (%)		Reference
	(bar)	(LMH/bar)	Salts	Pollutants ^b^	
ZrO_2_-P84^®^ PI	10	22.7	Na_2_SO_4_: 93.4	Rifampicin, puerarin, tetracycline, and tea polyphenols: >90.0	[242]
CL/PES	4	2.6	ZnCl_2_: 95.7 MgCl_2_: 95.1	-	[243]
PA/(PES/PVDF) TFC	5	13.8	Na_2_SO_4_: 98.2	-	[86]
PPA/PMIA TFC	6	17.3	Na_2_SO_4_: 98.5 MgSO_4_: 98.0 CaCl_2_: 96.0 MgCl_2_: 95.7	CFB, RY3, and B2RL: >97.0	[248]
GO/PSF TFC	5	8.39	Na_2_SO_4_: 96.1	RB: >99.0 ST: 92.9	[249]
MXene/PAN	1	~5.9	Na_2_SO_4_: ~70.0	-	[251]
Polyester-reinforced PES	6	8.7	NaCl: <7.0	CR: 99.9	[254]
PEI-70K/PVDF/SMA	1	10.4	-	MO: 97.1	[255]
PEI-70K & PEI-10K/PVDF/SMA	1	6.4	-	MO: 99.9	[255]
PPTA/PSf-PA	7	5.46	MgSO_4_: 98.1	MO, AR88, EBT, CR, RB5, and RR195: >99.0 MB, GTL, GRL, and RhB: >98.0	[256]

^a^ PI: polyimide; CL: chitosan lactate; PES: polyethersulfone; PA: polyamide; PVDF: polyvinylidene fluoride; PPA: poly(piperazine-amide); PMIA: poly(m-phenylene isophthalamide); GO: graphene oxide; PSF: polysulfone; PAN: polyacrylonitrile; PEI: polyethyleneimine; SMA: styrene maleic anhydride; PPTA: poly(p-phenylene terephthalamide). ^b^ CFB: Chromatrope FB; RY3: Reactive Yellow 3; B2RL: Direct Fast Blue B2RL; RB: Rose Bengal; ST: Safranine T; CR: Congo Red; MO: Methyl Orange; AR88: Acid Red 88; EBT: Eriochrome Black T; RB5: Reactive Black 5; RR195: Reactive Red 195; MB: Methylene Blue; GTL: Cationic red GTL; GRL: Cationic Red X-GRL; RhB: Rhodamine B.

**Table 2:** OSN performances for hollow fiber membranes.

Membrane ^a^	Pressure (bar)	Solvent	Permeance (LMH/bar)	Solute ^b^	Solute MW (Da)	Rejection (%)	Reference
PMDA-ODA PI	10	DMF	2.5	RB	1017	96.7	[261]
PMDA-MDA PI	10	THF	7.2	RB	1017	95.7	[262]
PEI/PIP-based TFC/PI	2	Acetone	11.6	MO AF	327 585	46.5 91.8	[263]
MPD-based TFC/PI	2	Ethanol Acetone Acetonitrile	2.33 24.2 10.58	MO AF MR Levofloxacin	327 585 269 361	99.4 98.6 90.1 98.2	[264]
K_2_S_2_O_8_-PBI	5	Acetone Ethanol Isopropanol	~3 ~2 ~1	MB RBB	374 626	~99 ~96 ~99	[108]
GO TFN	5	Ethanol Methanol	2.0 5.8	RDB RB	479 1017	100 99	[283]
TiO_2_@rGO TFN	8	Ethanol	4.1	BTB	624	95	[284]
NH_2_-MWCNT/PI	5	Acetone Ethanol Isopropanol	4.31 1.17 0.53	BBR Tetracycline MB	826 444 320	99.9 97.4 99.8	[285]

^a^ PI: polyimide; PMDA-ODA: poly(4,4′-oxydiphenylene pyromellitimide); PMDA-MDA: poly(pyromellitic dianhydride-co-4,4′-oxydianiline); PEI: polyethylenimine; PIP: piperazine; MPD: m-phenylenediamine; PBI: polybenzimidazole; K_2_S_2_O_8_: potassium persulphate; GO: graphene oxide; rGO: reduced GO; TiO_2_: titanium oxide; NH_2_-MWCNT: amine-functionalized multi-walled carbon nanotubes. ^b^ RB: Rose Bengal, AF: Acid Fuchsin, MO: Methyl Orange, MR: Methyl Red, MB: Methylene blue; RBB: Remazol Brilliant Blue; RDB: Rhodamine B; BTB: Bromothymol blue; BBR: Brilliant Blue R.

**Table 4:** Recent state-of-the-art CMS hollow fiber membranes for CO_2_ capture, air purification, H_2_/alkane separation, and olefin/paraffin separation.

Polymer Precursor ^a^	Pyrolysis Treatment	Pyrolysis Temperature (°C)	T/P (°C/atm)	Permeance (GPU) ^b^									Selectivity	Reference
				H_2_	O_2_	N_2_	CH_4_	CO_2_	C_2_H_4_	C_2_H_6_	C_3_H_6_	C_3_H_8_		
6FDA/DETDA-DABA	VTMS ^c^	550	35/2	-	-	-	-	1000.0	-	-	-	-	CO_2_/CH_4_: >25	[142]
PIM-1	-	575	35/6.8	-	4.0	0.4	0.3	17.8	0.9	0.2	-	-	O_2_/N_2_: 10.0 CO_2_/N_2_: 44.5 CO_2_/CH_4_: 59.3 C_2_H_4_/C_2_H_6_: 4.5	[148]
PI/LPSQ	-	675	35/1	-	-	-	19.0	956.0	-	-	-	-	CO_2_/CH_4_: 50.2	[145]
Cellulose	-	600	25/2	-	-	-	0.02 ^g^	3.6 ^g^	-	-	-	-	CO_2_/CH_4_: 186.0	[529]
			25/28	-	-	-	0.03 ^f,g^	2.3 ^f,g^	-	-	-	-	CO_2_/CH_4_: 75.0 ^f^	[529]
			60/8	-	-	-	0.09 ^f,g^	4.5 ^f,g^	-	-	-	-	CO_2_/CH_4_: 50.0 ^f^	[529]
Cellulose	-	600	25/2	-	-	-	0.001 ^g^	1.1 ^g^	-	-	-	-	CO_2_/CH_4_: 917.0	[530]
			60/50	-	-	-	-	1.5 ^f,g^	-	-	-	-	CO_2_/CH_4_: 131.0 ^f^	[530]
Cellulose	-	600	25/8	-	-	-	0.03 ^f,g^	1.3 ^f,g^	-	-	-	-	CO_2_/N_2_: 42.0 ^f^	[531]
				-	-	-	0.03 ^f,g^	0.5 ^f,g^	-	-	-	-	CO_2_/CH_4_: 15.0 ^f^	[531]
PMDA-ODA	Cross-linking with PEI ^c^	600	25/7	-	19.6	6.5	4.7	93.4	-	-	-	-	O_2_/N_2_: 3.0 CO_2_/N_2_: 14.4 CO_2_/CH_4_: 19.8	[532]
6FDA/BPDA-DAM	-	675	NR ^e^/3.5	117.0	-	-	-	-	0.4	-	-	-	H_2_/C_2_H_4_: 297.0	[141]
PEI	-	600	NR ^e^/5	711.6	-	-	-	254.1	-	151.4	-	134.3	H_2_/CO_2_: 2.8 H_2_/C_2_H_6_: 4.7 H_2_/C_3_H_8_: 5.3	[533]
Matrimid	VTMS ^c^	675	600/1	430.0 ^f^	-	-	-	-	-	-	-	-	H_2_/C_3_H_8_: 511.0 ^f^	[138]
Cellulose	-	850	130/2	148.2	-	0.2	0.03	1.8	-	-	-	-	H_2_/N_2_: >800.0 H_2_/CH_4_: >5700.0 H_2_/CO_2_: 83.9	[137]
PBI	-	750	25/1	-	0.8	-		-	-	-	-	-	O_2_/N_2_: 13.7	[144]
6FDA/BPDA-DAM	Super-hyperaging thermal treatment ^d^	675	35/3.5	-	-	-	-	-	-	-	25.0	2.5	C_3_H_6_/C_3_H_8_: 10.0	[140]
Matrimid coated alumina	-	650	25/2	-	-	-	-	-	-	-	45.0	2.7	C_3_H_6_/C_3_H_8_: 16.5	[534]

^a^ PI/LPSQ: polyimide/ladder-structured polysilsesquioxane; PIM-1: polymers of intrinsic microporosity; 6FDA: 4,4′-(hexafluoroisopropylidene) diphthalic anhydride (6FDA); DETDA: diethyltoluenediamine; DABA: 3,5-diaminobenzoic acid; PMDA-ODA: poly(4,4′-oxydiphenylene pyromellitimide); PEI: polyethylenimine; PBI: polybenzimidazole; BPDA: 3,3′-4,4′-biphenyl tetracarboxylic acid dianhydride (BPDA); DAM: 2,4,6-trimethyl-1,3-phenylene diamine; VTMS: vinyltrimethoxysilane. ^b^ 1 GPU = 1 × 10^−6^ cm^3^(STP)/cm^2^∙cmHg∙s). ^c^ pre-pyrolysis treatment. ^d^ post-pyrolysis treatment. ^e^ NR: not reported. ^f^ Data were obtained from mixed-component systems. ^g^ Value calculated based on the permeability data reported in Barrer with respective thickness.

**Table 5:** Recent reported perovskite hollow fiber membranes for O_2_ separation.

Ceramic ^a^	Modification	Temperature (°C)	O_2_ Flux (mL cm^−2^ min^−1^)	Reference
BSCF	-	900	7.05	[541]
LSC	-	1000	1.20	[542]
LCCF	-	1000	6.20 ^b^	[543]
LCCF	-	1000	5.10 ^b^	[544]
BCBZ	-	1000	5.75	[555]
LSCF	LSCF bundle of 5 single LSCF hollow fiber	950	0.46	[540]
BCBZ	BCBZ bundle of 7 single BCBZ hollow fiber	1000	7.06	[537]
LSC	LSC_214_ Ruddlesden–Popper decoration	850	0.40	[548]
LSC	LSC_214_ Ruddlesden–Popper decoration	900	0.60	[548]
LSCF-LSC	LSC Ruddlesden-Popper phase	950	4.52	[549]
Wrinkled LSCF	Ag deposition via dip coating on outer surface	950	1.48	[127]
LCF	Ag deposition via wet complexation	950	1.46	[550]

^a^ LSCF: La_0.6_Sr_0.4_Co_0.2_Fe_0.8_O_3−δ_; BCBZ: BaCo_0.85_Bi_0.05_Zr_0.1_O_3−δ_; BSCF: Ba_0.5_Sr_0.5_Co_0.8_Fe_0.2_O_3−δ_; LSC: La_0.6_Sr_0.4_CoO_3−δ_; LSC_214_: (La,Sr)_2_CoO_4_; LCCF: (La_0.6_Ca_0.4_)(Co_0.8_Fe_0.2_)O_3−δ_; LCF: La_0.8_Ca_0.2_Fe_0.94_O_3−a_. ^b^ 50% CO_2_ atmosphere.

## Data Availability

Not applicable.

## Abbreviations

2D	Two-dimensional
6FDA	4,4′-(hexafluoroisopropylidene) diphthalic anhydride
Abn-NH	Amino functionalized acid activated bentonite clay
AF	Acid Fuchsin
Ag	Silver
Al_2_O_3_	Aluminium oxide
APTES	3-aminopropyltriethoxysilane
AR88	Acid Red 88
B2RL	Direct Fast Blue B2RL
BBR	Brilliant Blue R
BCBZ	BaCo_0.85_Bi_0.05_Zr_0.1_O_3−δ_
BPDA	3,3′-4,4′-biphenyl tetracarboxylic acid dianhydride
BSA	Bovine serum albumin
BSCF	Ba_0.5_Sr_0.5_Co_0.8_Fe_0.2_O_3−δ_
BTB	Bromothymol Blue
C_2_H_4_	Ethane or ethylene
C_3_H_6_	Propylene or propene or paraffin
C_3_H_8_	Propane or olefin
CECs	Contaminants of emerging concern
CFB	Chromatrope FB
CHA	Chabazite
CL	Chitosan lactate
Cl^−^	Chloride ions
CMPs	Conjugated microporous polymers
CMS	Carbon molecular sieve
CO_2_	Carbon dioxide
COF(s)	Covalent organic framework(s)
CQDs	Carbon quantum dots
CR	Congo Red
CTA	Cellulose triacetate
c-TiO_2_	Carboxylated TiO_2_
DABA	Diaminobenzoic acid
DAM	2,4,6-trimethyl-1,3-phenylene diamine
DAPE	1,3-diaminopropane
DCMD	Direct contact membrane distillation
DD3R	Decadodecasil 3R
DES(s)	Deep eutectic solvent(s)
DETDA	Diethyltoluenediamine
DIPS	Diffusion-induced phase separation
DMAc	Dimethylacetamide
DMF	Dimethylformamide
DMSO	Dimethyl sulfoxide
DR23	Direct Red 23
EBT	Eriochrome Black T
ECHA	European Chemical Agency
EDA	Ethylenediamine
ETS-4	Engelhard titanosilicate-4
EVAL	Poly(ethylene vinyl) alcohol
Fe	Iron
FESEM	Field emission scanning electron microscope
FO	Forward osmosis
FRR	flux recovery ratio
GA	Glutaraldehyde
GBL	γ-butyrolactone
GO	Graphene oxide
GRL	Cationic Red X-GRL
GTL	Cationic red GTL
H_2_	Hydrogen
H_2_S	Hydrogen sulfide
IL(s)	Ionic liquid(s)
K_2_S_2_O_8_	Potassium persulphate
KNO_3_/KNO_2_-BSCF	potassium nitrate/potassium nitrite-BSCF
LCCF	(La_0.6_Ca_0.4_)(Co_0.8_Fe_0.2_)O_3−δ_
LCF	La_0.8_Ca_0.2_Fe_0.94_O_3−a_
LSC	La_0.6_Sr_0.4_CoO_3−δ_
LSCF	La_0.6_Sr_0.4_Co_0.2_Fe_0.8_O_3−δ_
MB	Methylene Blue
MD	Membrane distillation
MF	Microfiltration
MgCl_2_	Magnesium chloride
MIL	Materials of Institute Lavoisier
MO	Methyl Orange
MOF(s)	Metal organic framework(s)
MoS_2_	Molybdenum disulfide
MPD	M-phenylenediamine
MR	Methyl Red
MS-S	Melt-spinning and stretching
MWCNT	Multi-walled carbon nanotubes
MWCO	Molecular weight cut off
N_2_	Nitrogen
Na^+^	Sodium ions
Na^+^-CQDs	Sodium-functionalized CQDs
Na_2_SO_4_	Sodium sulfate
NaCl	Sodium chloride
NADES	Natural deep eutectic solvent
NaOH	Sodium hydroxide
NF	Nanofiltration
NH_2_-MWCNT	Amine-functionalized MWCNT
NIPS	Non-solvent induced phase separation
NMP	*N*-methyl-2-pyrrolidone
NO	Nitric oxide
O_2_	Oxygen
OARO	Osmotically assisted reverse osmosis
OSFO	Organic solvent forward osmosis
OSN	Organic solvent nanofiltration
OSRO	Organic solvent reverse osmosis
P(VTES-AA-SSNa)	Poly(triethoxyvinylsilane-acrylic acid-sodium 4-vinylbenzenssulfonate)
PA	Polyamide
PAA	Polyamic acid
PAN	Polyacrylonitrile
PANI	Polyaniline
PBI	Polybenzimidazole
PC	Polycarbonate
PCL	Polycaprolactone
Pd	Palladium
PDA	Polydopamine
PDMS	Polydimethylsiloxane
PEG	Polyethylene glycol
PEI	Polyethyleneimine
PEO	Polyethylene oxide
PES	Polyethersulfone
PET	Polyethylene terephthalate
PHAs	Polyhydroxyalkanoates
PI/LPSQ	Polyimide/ladder-structured polysilsesquioxane
PIMs	Polymers of intrinsic microporosity
PIP	Piperazine
PLA	Polylactic acid
PMDA-MDA	Pyromellitic dianhydride-4,4′-diaminodiphenylmethane
PMDA-ODA	Poly(4,4′-oxydiphenylene pyromellitimide)
PMIA	Poly(m-phenylene isophthalamide)
PMMOF	Polymer-modification-enabled in-site metal-organic framework
PMP	Poly(4-methyl-1-pentene)
PPA	Poly(piperazine-amide)
PPTA	Poly(p-phenylene terephthalamide)
PPTA	Poly(p-phenylene terephthalamide)
PSF	Polysulfone
PSS	Poly(4-styrenesulfonic acid)
PTFE	Polytetrafluoroethylene
PVA	Poly(vinyl alcohol)
PVDF	Polyvinylidene fluoride
PWP	Pure water permeability
RB	Rose Bengal
RB5	Reactive Black 5
RBB	Remazol Brilliant Blue
RDB	Rhodamine B
rGO	Reduced GO
RhB	Rhodamine B
RO	Reverse osmosis
RR195	Reactive Red 195
RY3	Reactive Yellow 3
SAPO-34	Silicoaluminophosphate chabazite
SEM	Scanning electron microscope
SILMs	Supported ionic liquid membrane(s)
SiO_2_	Silicon dioxide
SMA	Styrene maleic anhydride
SO_2_	Sulfur dioxide
ST	Safranine T
TDI	2,4-toluene diisocyanate
TEOS	Tetraethyl orthosilicate
TEP	Triethyl phosphate
TEPA	Tetraethylenepentamine
TFC	Thin film composite
TFN	Thin film nanocomposite
THF	Tetrahydrofuran
TiO_2_	Titanium dioxide
TIPS	Thermally induced phase separation
TMC	Trimesoyl chloride
TR	Thermally rearraged
UF	Ultrafiltration
UiO	Universitetet i Oslo
VTMS	Vinyltrimethoxysilane
XDLVO	Extended Derjaguin-Landau-Verwey-Overbeek
YSZ	Yttria-stabilized zirconia
ZIF(s)	Zeolitic imidazolate framework(s)
ZnCl_2_	Zinc chloride
ZrO_2_	Zirconium dioxide
β-CD	β-cyclodextrin

## References

[1] Guo Y., Tong L., Mei L. (2021). Evaluation and influencing factors of industrial pollution in Jilin restricted development zone: A spatial econometric analysis. Sustainability.

[2] Kim S. (2021). Membranes for water, gas and ion separation. Membranes.

[3] Brunetti A., Macedonio F., Barbieri G., Drioli E. (2015). Membrane engineering for environmental protection and sustainable industrial growth: Options for water and gas treatment. Environ. Eng. Res..

[4] Font-Palma C., Cann D., Udemu C. (2021). Review of cryogenic carbon capture innovations and their potential applications. C.

[5] Sircar S., Golden T. (2000). Purification of hydrogen by pressure swing adsorption. Sep. Sci. Technol..

[6] Sholl D.S., Lively R.P. (2016). Seven chemical separations to change the world. Nature.

[7] Liang C.Z., Chung T.-S., Lai J.-Y. (2019). A review of polymeric composite membranes for gas separation and energy production. Prog. Polym. Sci..

[8] Pagliero M., Khayet M., Garcia-Payo C., García-Fernández L. (2021). Hollow fibre polymeric membranes for desalination by membrane distillation technology: A review of different morphological structures and key strategic improvements. Desalination.

[9] Chung T.-S., Feng Y. (2021). Hollow Fiber Membranes: Fabrication and Applications.

[10] Pagliero M., Comite A., Soda O., Costa C. (2021). Effect of support on PVDF membranes for distillation process. J. Membr. Sci..

[11] Peng N., Widjojo N., Sukitpaneenit P., Teoh M.M., Lipscomb G.G., Chung T.-S., Lai J.-Y. (2012). Evolution of polymeric hollow fibers as sustainable technologies: Past, present, and future. Prog. Polym. Sci..

[12] Lau H.S., Yong W.F. (2021). Recent progress and prospects of polymeric hollow fiber membranes for gas application, water vapor separation and particulate matter removal. J. Mater. Chem. A.

[13] Moch I. (2000). Membranes, Hollow-Fiber.

[14] Mahon H.I. (1966). Permeability Separatory Apparatus, Permeability Separatory Membrane Element, Method of Making the Same and Process Utilizing the Same. U.S. Patent.

[15] Lonsdale H. (1982). The growth of membrane technology. J. Membr. Sci..

[16] Mclain E.A. (1969). Wound Hollow Fiber Permeability Apparatus and Process of Making the Same. U.S. Patent.

[17] Li N.N., Fane A.G., Ho W.W., Matsuura T. (2011). Advanced Membrane Technology and Applications.

[18] Henis J.M., Tripodi M.K. (1980). A novel approach to gas separations using composite hollow fiber membranes. Sep. Sci. Technol..

[19] Henis J.M., Tripodi M.K. (1981). Composite hollow fiber membranes for gas separation: The resistance model approach. J. Membr. Sci..

[20] Henis J.M., Tripodi M.K. (1980). Multicomponent Membranes for Gas Separations. U.S. Patent.

[21] Waterland L.R., Robertson C.R., Michaels A.S. (1975). Enzymatic catalysis using asymmetric hollow fiber membranes. Chem. Eng. Commun..

[22] Bollinger W., Chen G., Metzer T. (1981). Commerical Hydrogen Purification and Recovery Using PRISM Separators.

[23] Moattari R.M., Mohammadi T., Rajabzadeh S., Dabiryan H., Matsuyama H. (2021). Reinforced hollow fiber membranes: A comprehensive review. J. Taiwan Inst. Chem. Eng..

[24] Jung J.T., Wang H.H., Kim J.F., Lee J., Kim J.S., Drioli E., Lee Y.M. (2018). Tailoring nonsolvent-thermally induced phase separation (N-TIPS) effect using triple spinneret to fabricate high performance PVDF hollow fiber membranes. J. Membr. Sci..

[25] Hassankiadeh N.T., Cui Z., Kim J.H., Shin D.W., Sanguineti A., Arcella V., Lee Y.M., Drioli E. (2014). PVDF hollow fiber membranes prepared from green diluent via thermally induced phase separation: Effect of PVDF molecular weight. J. Membr. Sci..

[26] Li X., Wang Y., Lu X., Xiao C. (2008). Morphology changes of polyvinylidene fluoride membrane under different phase separation mechanisms. J. Membr. Sci..

[27] Li G., Kujawski W., Válek R., Koter S. (2021). A review-The development of hollow fibre membranes for gas separation processes. Int. J. Greenh. Gas Control.

[28] Hosseini S.S., Peng N., Chung T.-S. (2010). Gas separation membranes developed through integration of polymer blending and dual-layer hollow fiber spinning process for hydrogen and natural gas enrichments. J. Membr. Sci..

[29] Li D.F., Chung T.-S., Wang R., Liu Y. (2002). Fabrication of fluoropolyimide/polyethersulfone (PES) dual-layer asymmetric hollow fiber membranes for gas separation. J. Membr. Sci..

[30] Jo E.-S., An X., Ingole P.G., Choi W.-K., Park Y.-S., Lee H.-K. (2017). CO_2_/CH_4_ separation using inside coated thin film composite hollow fiber membranes prepared by interfacial polymerization. Chin. J. Chem. Eng..

[31] Wenten I., Khoiruddin K., Wardani A., Aryanti P., Astuti D., Komaladewi A. (2020). Preparation of antifouling polypropylene/ZnO composite hollow fiber membrane by dip-coating method for peat water treatment. J. Water Process. Eng..

[32] Sharma A.K., Juelfs A., Colling C., Sharma S., Conover S.P., Puranik A.A., Chau J., Rodrigues L., Sirkar K.K. (2021). Porous hydrophobic–hydrophilic composite hollow fiber and flat membranes prepared by plasma polymerization for direct contact membrane distillation. Membranes.

[33] Park H.J., Bhatti U.H., Nam S.C., Park S.Y., Lee K.B., Baek I.H. (2019). Nafion/TiO_2_ nanoparticle decorated thin film composite hollow fiber membrane for efficient removal of SO_2_ gas. Sep. Purif. Technol..

[34] Liang C.Z., Yong W.F., Chung T.-S. (2017). High-performance composite hollow fiber membrane for flue gas and air separations. J. Membr. Sci..

[35] Li M., Zhu Z., Zhou M., Jie X., Wang L., Kang G., Cao Y. (2021). Removal of CO_2_ from biogas by membrane contactor using PTFE hollow fibers with smaller diameter. J. Membr. Sci..

[36] Naderi A., Chung T.-S., Weber M., Maletzko C. (2019). High performance dual-layer hollow fiber membrane of sulfonated polyphenylsulfone/polybenzimidazole for hydrogen purification. J. Membr. Sci..

[37] Yang D., Barbero R.S., Devlin D.J., Cussler E., Colling C.W., Carrera M.E. (2006). Hollow fibers as structured packing for olefin/paraffin separations. J. Membr. Sci..

[38] Qi Z., Cussler E. (1985). Microporous hollow fibers for gas absorption: II. Mass transfer across the membrane. J. Membr. Sci..

[39] Huang Y., Xiao C., Huang Q., Liu H., Zhao J. (2021). Progress on polymeric hollow fiber membrane preparation technique from the perspective of green and sustainable development. Chem. Eng. J..

[40] Zhang X., Xiao C., Hu X., Bai Q. (2013). Preparation and properties of homogeneous-reinforced polyvinylidene fluoride hollow fiber membrane. Appl. Surf. Sci..

[41] Hu X., Chen Y., Liang H., Xiao C. (2011). Preparation of polyurethane/poly (vinylidene fluoride) blend hollow fibre membrane using melt spinning and stretching. Mater. Sci. Technol..

[42] Aslan T., Arslan S., Eyvaz M., Güçlü S., Yüksel E., Koyuncu I. (2016). A novel nanofiber microfiltration membrane: Fabrication and characterization of tubular electrospun nanofiber (TuEN) membrane. J. Membr. Sci..

[43] Park H.B., Kamcev J., Robeson L.M., Elimelech M., Freeman B.D. (2017). Maximizing the right stuff: The trade-off between membrane permeability and selectivity. Science.

[44] Hays S.S., Sanyal O., León N.E., Arab P., Koros W.J. (2020). Envisioned role of slit bypass pores in physical aging of carbon molecular sieve membranes. Carbon.

[45] Chen C.-C., Qiu W., Miller S.J., Koros W.J. (2011). Plasticization-resistant hollow fiber membranes for CO_2_/CH_4_ separation based on a thermally crosslinkable polyimide. J. Membr. Sci..

[46] Huang Y., Wang X., Paul D.R. (2006). Physical aging of thin glassy polymer films: Free volume interpretation. J. Membr. Sci..

[47] Dong G., Li H., Chen V. (2011). Plasticization mechanisms and effects of thermal annealing of Matrimid hollow fiber membranes for CO_2_ removal. J. Membr. Sci..

[48] Alklaibi A.M., Lior N. (2005). Membrane-distillation desalination: Status and potential. Desalination.

[49] Lau S.K., Yong W.F. (2021). Recent progress of zwitterionic materials as antifouling membranes for ultrafiltration, nanofiltration, and reverse osmosis. ACS Appl. Polym. Mater..

[50] Zhang R., Liu Y., He M., Su Y., Zhao X., Elimelech M., Jiang Z. (2016). Antifouling membranes for sustainable water purification: Strategies and mechanisms. Chem. Soc. Rev..

[51] Khan I.U., Othman M.H.D., Ismail A., Matsuura T., Hashim H., Nordin N.A.H.M., Rahman M.A., Jaafar J., Jilani A. (2018). Status and improvement of dual-layer hollow fiber membranes via co-extrusion process for gas separation: A review. J. Nat. Gas Sci. Eng..

[52] Castro-Muñoz R., Galiano F., Figoli A. (2020). Recent advances in pervaporation hollow fiber membranes for dehydration of organics. Chem. Eng. Res. Des..

[53] Sirkar K.K. (2008). Membranes, phase interfaces, and separations: Novel techniques and membranes—An overview. Ind. Eng. Chem. Res..

[54] Sewerin T., Elshof M.G., Matencio S., Boerrigter M., Yu J., de Grooth J. (2021). Advances and applications of hollow fiber nanofiltration membranes: A review. Membranes.

[55] Yang Z., Zhou Y., Feng Z., Rui X., Zhang T., Zhang Z. (2019). A review on reverse osmosis and nanofiltration membranes for water purification. Polymers.

[56] Turken T., Sengur-Tasdemir R., Ates-Genceli E., Tarabara V.V., Koyuncu I. (2019). Progress on reinforced braided hollow fiber membranes in separation technologies: A review. J. Water Process. Eng..

[57] Ahmad A.L., Otitoju T.A., Ooi B.S. (2019). Hollow fiber (HF) membrane fabrication: A review on the effects of solution spinning conditions on morphology and performance. J. Ind. Eng. Chem..

[58] Dashti A., Asghari M. (2015). Recent progresses in ceramic hollow-fiber membranes. ChemBioEng Rev..

[59] Feng C., Khulbe K., Matsuura T., Ismail A. (2013). Recent progresses in polymeric hollow fiber membrane preparation, characterization and applications. Sep. Purif. Technol..

[60] Feng Y., Chung T.-S., Chung T.-S., Feng Y. (2021). Chapter 1—Introduction. Hollow Fiber Membranes.

[61] Baker R.W., Low B.T. (2014). Gas separation membrane materials: A perspective. Macromolecules.

[62] Dai Z., Ansaloni L., Deng L. (2016). Recent advances in multi-layer composite polymeric membranes for CO_2_ separation: A review. Green Energy Environ..

[63] Baker R.W. (2012). Membrane Technology and Applications.

[64] Matsuyama H., Karkhanechi H., Rajabzadeh S., Chung T.-S., Feng Y. (2021). Chapter 3—Polymeric membrane fabrication via thermally induced phase separation (TIPS) method. Hollow Fiber Membranes.

[65] Mulder M., Mulder J. (1996). Basic Principles of Membrane Technology.

[66] Yong W.F., Chung T.-S., Weber M., Maletzko C. (2018). New polyethersulfone (PESU) hollow fiber membranes for CO_2_ capture. J. Membr. Sci..

[67] Li M., Feng Y., Wang K., Yong W.F., Yu L., Chung T.-S. (2017). Novel hollow fiber air filters for the removal of ultrafine particles in PM_2.5_ with repetitive usage capability. Environ. Sci. Technol..

[68] Li Y., Cao B., Li P. (2017). Fabrication of PMDA-ODA hollow fibers with regular cross-section morphologies and study on the formation mechanism. J. Membr. Sci..

[69] Tham H.M., Wang K.Y., Hua D., Japip S., Chung T.-S. (2017). From ultrafiltration to nanofiltration: Hydrazine cross-linked polyacrylonitrile hollow fiber membranes for organic solvent nanofiltration. J. Membr. Sci..

[70] Liang C.Z., Liu J.T., Lai J.-Y., Chung T.-S. (2018). High-performance multiple-layer PIM composite hollow fiber membranes for gas separation. J. Membr. Sci..

[71] Cheng D., Zhao L., Li N., Smith S.J., Wu D., Zhang J., Ng D., Wu C., Martinez M.R., Batten M.P. (2019). Aluminum fumarate MOF/PVDF hollow fiber membrane for enhancement of water flux and thermal efficiency in direct contact membrane distillation. J. Membr. Sci..

[72] Ren J., Wang R., Wang L.K., Chen J.P., Hung Y.-T., Shammas N.K. (2011). Preparation of polymeric membranes. Membrane and Desalination Technologies.

[73] Tompa H. (1956). Polymer Solutions.

[74] Strathmann H., Scheible P., Baker R. (1971). A rationale for the preparation of Loeb-Sourirajan-type cellulose acetate membranes. J. Appl. Polym. Sci..

[75] Michaels A.S. (1971). High Flow Membrane. U.S. Patent.

[76] Figoli A., Cassano A., Basile A. (2016). Membrane Technologies for Biorefining.

[77] Purkait M.K., Sinha M.K., Mondal P., Singh R. (2018). Introduction to membranes. Interface Science and Technology.

[78] Kapantaidakis G., Koops G., Wessling M. (2002). Effect of spinning conditions on the structure and the gas permeation properties of high flux polyethersulfone-polyimide blend hollow fibers. Desalination.

[79] Tasselli F., Jansen J., Sidari F., Drioli E. (2005). Morphology and transport property control of modified poly(ether ether ketone)(PEEKWC) hollow fiber membranes prepared from PEEKWC/PVP blends: Influence of the relative humidity in the air gap. J. Membr. Sci..

[80] Da Silva Barbosa Ferreira R., Dias R.A., Araújo E.M., Oliveira S.S.L., da Nóbrega Medeiros V., de Lucena Lira H. (2022). Hollow fiber membranes of polysulfone/attapulgite for oil removal in wastewater. Polym. Bull..

[81] Mondal R., De S. (2022). Removal of copper (II) from aqueous solution using zinc oxide nanoparticle impregnated mixed matrix hollow fiber membrane. Environ. Technol. Innov..

[82] Lim Y.-G., Bak C.-u., Kim Y.-D. (2022). Comprehensive experimental and theoretical insights into the performance of polysulfone hollow-fiber membrane modules in biogas purification process. Chem. Eng. J..

[83] Zou D., Kim H.W., Jeon S.M., Lee Y.M. (2022). Fabrication and modification of PVDF/PSF hollow-fiber membranes for ginseng extract and saline water separations via direct contact membrane distillation. J. Membr. Sci..

[84] Dibrov G., Kagramanov G., Sudin V., Molchanov S., Grushevenko E., Yushkin A., Volkov V. (2022). Influence of draw ratio and take-up velocity on properties of ultrafiltration hollow fiber membranes from polyethersulfone. Fibers.

[85] Liang C.Z., Yong W.F., Wu J., Weber M., Maletzko C., Lai J.-Y., Chung T.-S. (2022). Plasticization-enhanced trimethylbenzene functionalized polyethersulfone hollow fiber membranes for propylene and propane separation. J. Membr. Sci..

[86] Wang C., Chen Y., Hu X., Guo P. (2022). Engineering novel high-flux thin-film composite (TFC) hollow fiber nanofiltration membranes via a facile and scalable coating procedure. Desalination.

[87] Czieborowski M., Kemperman A.J., Rolevink E., Blom J., Visser T., Philipp B. (2022). A two-step bioluminescence assay for optimizing antibacterial coating of hollow-fiber membranes with polydopamine in an integrative approach. J. Microbiol. Methods.

[88] Vatanpour V., Pasaoglu M.E., Barzegar H., Teber O.O., Kaya R., Bastug M., Khataee A., Koyuncu I. (2022). Cellulose acetate in fabrication of polymeric membranes: A review. Chemosphere.

[89] Sunder N., Fong Y.Y., Bustam M.A. (2022). Investigation on the effects of air gap distance on the formation of cellulose triacetate hollow fiber membrane for CO_2_ and CH_4_ gases permeation. Mater. Today Proc..

[90] Nakao T., Goda S., Miura Y., Yasukawa M., Ishibashi M., Nakagawa K., Shintani T., Matsuyama H., Yoshioka T. (2022). Development of cellulose triacetate asymmetric hollow fiber membranes with highly enhanced compaction resistance for osmotically assisted reverse osmosis operation applicable to brine concentration. J. Membr. Sci..

[91] Theodorakopoulos G.V., Karousos D.S., Mansouris K.G., Sapalidis A.A., Kouvelos E.P., Favvas E.P. (2022). Graphene nanoplatelets based polyimide/Pebax dual-layer mixed matrix hollow fiber membranes for CO_2_/CH_4_ and He/N_2_ separations. Int. J. Greenh. Gas Control.

[92] Wang Z.-Y., Li S., Xu S., Tian L., Su B., Han L., Mandal B. (2021). Fundamental understanding on the preparation conditions of high-performance polyimide-based hollow fiber membranes for organic solvent nanofiltration (OSN). Sep. Purif. Technol..

[93] Wang Q., Wei X., Wang G.-R., Lu T.-D., Shi Q., Sun S.-P. (2021). Inner-selective coordination nanofiltration hollow fiber membranes from assist-pressure modified substrate. J. Membr. Sci..

[94] Chen M., Yang R., Li P. (2022). Preparation of defect-free hollow fiber membranes derived from PMDA-ODA polyimide for gas separation. Chem. Eng. Res. Des..

[95] Mousavi S.A., Aboosadi Z.A., Mansourizadeh A., Honarvar B. (2021). Surface modified porous polyetherimide hollow fiber membrane for sweeping gas membrane distillation of dyeing wastewater. Colloids. Surf. A Physicochem. Eng. Asp..

[96] Mousavi S., Arab Aboosadi Z., Mansourizadeh A., Honarvar B. (2021). Modification of porous polyetherimide hollow fiber membrane by dip-coating of Zonyl^®^ BA for membrane distillation of dyeing wastewater. Water Sci. Technol..

[97] Li G., Knozowska K., Kujawa J., Tonkonogovas A., Stankevičius A., Kujawski W. (2021). Fabrication of polydimethysiloxane (PDMS) dense layer on polyetherimide (PEI) hollow fiber support for the efficient CO_2_/N_2_ separation membranes. Polymers.

[98] Li G., Kujawski W., Knozowska K., Kujawa J. (2021). The effects of PEI hollow fiber substrate characteristics on PDMS/PEI hollow fiber membranes for CO_2_/N_2_ separation. Membranes.

[99] Alihemati Z., Hashemifard S., Matsuura T., Ismail A. (2022). On performance and fouling of thin film composite hollow Fiber membranes using polycarbonate/polyvinylchloride as porous substrates for forward osmosis applications. J. Environ. Chem. Eng..

[100] Zheng M., Yang Y., Qiao S., Zhou J., Quan X. (2021). A porous carbon-based electro-Fenton hollow fiber membrane with good antifouling property for microalgae harvesting. J. Membr. Sci..

[101] Zou L., Gusnawan P., Jiang Y.-B., Zhang G., Yu J. (2020). Macrovoid-inhibited PVDF hollow fiber membranes via spinning process delay for direct contact membrane distillation. ACS Appl. Mater. Interfaces.

[102] El-badawy T., Othman M.H.D., Adam M.R., Kamaludin R., Ismail A., Rahman M.A., Jaafar J., Rajabzadeh S., Matsuyama H., Usman J. (2022). Braid-reinforced PVDF hollow fiber membranes for high-efficiency separation of oily wastewater. J. Environ. Chem. Eng..

[103] Madupathi M., Nandala S., Sundergopal S. (2022). Experimental and modeling investigation of ultrafine polyvinylidene fluoride hollow fiber membrane module for recovery of lactic acid from aqueous solutions. Polym. Eng. Sci..

[104] Chen Y., Lu K.-J., Liang C.Z., Chung T.-S. (2022). Mechanically strong Janus tri-bore hollow fiber membranes with asymmetric pores for anti-wetting and anti-fouling membrane distillation. Chem. Eng. J..

[105] Wang C., Chen Y., Yang K., Hu X., Zhang Y. (2022). Fabrication of tight GO/PVDF hollow fiber membranes with improved permeability for efficient fractionation of dyes and salts in textile wastewater. Polym. Bull..

[106] Zakaria N., Zaliman S., Leo C., Ahmad A., Ooi B., Poh P.E. (2022). 3D imprinted superhydrophobic polyvinylidene fluoride/carbon black membrane for membrane distillation with electrochemical cleaning evaluation. J. Environ. Chem. Eng..

[107] Zou L., Zhang G., Yu J. (2022). Desirable PVDF hollow fiber membrane engineered with synergism between small molecular weight additives for DCMD treating of a hypersaline brine. J. Water Process. Eng..

[108] Zhao B., Shi G.M., Wang K.Y., Lai J.-Y., Chung T.-S. (2021). Employing a green cross-linking method to fabricate polybenzimidazole (PBI) hollow fiber membranes for organic solvent nanofiltration (OSN). Sep. Purif. Technol..

[109] Dahe G.J., Singh R.P., Dudeck K.W., Yang D., Berchtold K.A. (2019). Influence of non-solvent chemistry on polybenzimidazole hollow fiber membrane preparation. J. Membr. Sci..

[110] Yong W.F., Li F.Y., Xiao Y.C., Chung T.-S., Tong Y.W. (2013). High performance PIM-1/Matrimid hollow fiber membranes for CO_2_/CH_4_, O_2_/N_2_ and CO_2_/N_2_ separation. J. Membr. Sci..

[111] Anderson M.R., Mattes B.R., Reiss H., Kaner R.B. (1991). Conjugated polymer films for gas separations. Science.

[112] Pellegrino J. (2003). The use of conducting polymers in membrane-based separations. Ann. N. Y. Acad. Sci..

[113] Chen D., Miao Y.-E., Liu T. (2013). Electrically conductive polyaniline/polyimide nanofiber membranes prepared via a combination of electrospinning and subsequent in situ polymerization growth. ACS Appl. Mater. Interfaces.

[114] Hardison L.M., Zhao X., Jiang H., Schanze K.S., Kleiman V.D. (2008). Energy transfer dynamics in a series of conjugated polyelectrolytes with varying chain length. J. Phys. Chem. C.

[115] Zhao X., Pinto M.R., Hardison L.M., Mwaura J., Müller J., Jiang H., Witker D., Kleiman V.D., Reynolds J.R., Schanze K.S. (2006). Variable band gap poly(arylene ethynylene) conjugated polyelectrolytes. Macromolecules.

[116] Masuda T. (2007). Substituted polyacetylenes. J. Polym. Sci. Part A Polym. Chem..

[117] Saini N., Pandey K., Awasthi K. (2021). Conjugate polymer-based membranes for gas separation applications: Current status and future prospects. Mater. Today Chem..

[118] Bini K., Xu X., Andersson M.R., Wang E. (2018). Alcohol-soluble conjugated polymers as cathode interlayers for all-polymer solar cells. ACS Appl. Energy Mater..

[119] Zhang M., Liu L., Ju X., He T., Chen P. (2018). Molten salt assisted synthesis of microporous polyaniline nanosheets with superior gas sorption properties. Microporous Mesoporous Mater..

[120] Majumdar S., Sarmah K., Mahanta D. (2020). A simple route to prepare polypyrrole-coated filter papers via vapor phase polymerization and their gas sensing application. ACS Appl. Polym. Mater..

[121] Huang Y., Zang Y., Xu L., Lei T., Cui J., Xie Y., Wang J., Jia H., Miao F. (2021). Synthesis of chiral conjugated microporous polymer composite membrane and improvements in permeability and selectivity during enantioselective permeation. Sep. Purif. Technol..

[122] Monga Mulunda M. (2018). Development of Conjugated Polymer Membranes for Gas Separation.

[123] García-Fernández L., Wang B., García-Payo M., Li K., Khayet M. (2017). Morphological design of alumina hollow fiber membranes for desalination by air gap membrane distillation. Desalination.

[124] Ko C.C., Chen C.H., Chen Y.R., Wu Y.H., Lu S.C., Hu F.C., Li C.L., Tung K.L. (2017). Increasing the performance of vacuum membrane distillation using micro-structured hydrophobic aluminum hollow fiber membranes. Appl. Sci..

[125] Magnone E., Lee H.J., Che J.W., Park J.H. (2016). High-performance of modified Al_2_O_3_ hollow fiber membranes for CO_2_ absorption at room temperature. J. Ind. Eng. Chem..

[126] Han N., Zhang C., Tan X., Wang Z., Kawi S., Liu S. (2019). Re-evaluation of La_0.6_Sr_0.4_Co_0.2_Fe_0.8_O_3−δ_ hollow fiber membranes for oxygen separation after long-term storage of five and ten years. J. Membr. Sci..

[127] Han N., Zhang W., Guo W., Xie S., Zhang C., Zhang X., Fransaer J., Liu S. (2021). Novel oxygen permeable hollow fiber perovskite membrane with surface wrinkles. Sep. Purif. Technol..

[128] Zhu J., Zhang G., Liu G., Liu Z., Jin W., Xu N. (2017). Perovskite hollow fibers with precisely controlled cation stoichiometry via one-step thermal processing. Adv. Mater..

[129] Xu Z., Zheng Q., Wang S., Zhang Z., Liu Z., Zhang G., Jin W. (2021). Fabrication of molten nitrate/nitrite dual-phase four-channel hollow fiber membranes for nitrogen oxides separation. J. Membr. Sci..

[130] Araki S., Li T., Li K., Yamamoto H. (2019). Preparation of zeolite hollow fibers for high-efficiency cadmium removal from waste water. Sep. Purif. Technol..

[131] Makhtar S.N.N.M., Rahman M.A., Ismail A.F., Othman M.H.D., Jaafar J. (2017). Preparation and characterization of glass hollow fiber membrane for water purification applications. Environ. Sci. Pollut. Res..

[132] Hubadillah S.K., Othman M.H.D., Ismail A., Rahman M.A., Jaafar J. (2019). A low cost hydrophobic kaolin hollow fiber membrane (h-KHFM) for arsenic removal from aqueous solution via direct contact membrane distillation. Sep. Purif. Technol..

[133] Lohaus T., De Wit P., Kather M., Menne D., Benes N., Pich A., Wessling M. (2017). Tunable permeability and selectivity: Heatable inorganic porous hollow fiber membrane with a thermo-responsive microgel coating. J. Membr. Sci..

[134] Wang M., Huang M.L., Cao Y., Ma X.H., Xu Z.L. (2016). Fabrication, characterization and separation properties of three-channel stainless steel hollow fiber membrane. J. Membr. Sci..

[135] Wang M., Song J., Wu X., Tan X., Meng B., Liu S. (2016). Metallic nickel hollow fiber membranes for hydrogen separation at high temperatures. J. Membr. Sci..

[136] Allioux F.-M., David O., Merenda A., Maina J.W., Benavides M.E., Tanaka A.P., Dumée L.F. (2018). Catalytic nickel and nickel–copper alloy hollow-fiber membranes for the remediation of organic pollutants by electrocatalysis. J. Mater. Chem. A.

[137] Lei L., Pan F., Lindbråthen A., Zhang X., Hillestad M., Nie Y., Bai L., He X., Guiver M.D. (2021). Carbon hollow fiber membranes for a molecular sieve with precise-cutoff ultramicropores for superior hydrogen separation. Nat. Commun..

[138] Liu L., Liu D., Zhang C. (2022). High-temperature hydrogen/propane separations in asymmetric carbon molecular sieve hollow fiber membranes. J. Membr. Sci..

[139] Cao Y., Zhang K., Sanyal O., Koros W.J. (2019). Carbon molecular sieve membrane preparation by economical coating and pyrolysis of porous polymer hollow fibers. Angew. Chem. Int. Ed..

[140] Qiu W., Xu L., Liu Z., Liu Y., Arab P., Brayden M., Martinez M., Liu J., Roy A., Koros W.J. (2021). Surprising olefin/paraffin separation performance recovery of highly aged carbon molecular sieve hollow fiber membranes by a super-hyperaging treatment. J. Membr. Sci..

[141] Qiu W., Vaughn J., Liu G., Xu L., Brayden M., Martinez M., Fitzgibbons T., Wenz G., Koros W.J. (2019). Hyperaging tuning of a carbon molecular-sieve hollow fiber membrane with extraordinary gas-separation performance and stability. Angew. Chem. Int. Ed..

[142] Kamath M.G., Fu S., Itta A.K., Qiu W., Liu G., Swaidan R., Koros W.J. (2018). 6FDA-DETDA: DABE polyimide-derived carbon molecular sieve hollow fiber membranes: Circumventing unusual aging phenomena. J. Membr. Sci..

[143] Zhang C., Wenz G.B., Williams P.J., Mayne J.M., Liu G., Koros W.J. (2017). Purification of aggressive supercritical natural gas using carbon molecular sieve hollow fiber membranes. Ind. Eng. Chem. Res..

[144] Seong J.G., Lewis J.C., Matteson J.A., Craddock E., Martinez U., Thakkar H., Benavidez A.D., Berchtold K.A., Singh R.P. (2022). Polybenzimidazole-derived carbon molecular sieve hollow fiber membranes with tailored oxygen selective transport. Carbon.

[145] Shin J.H., Yu H.J., An H., Lee A.S., Hwang S.S., Lee S.Y., Lee J.S. (2019). Rigid double-stranded siloxane-induced high-flux carbon molecular sieve hollow fiber membranes for CO_2_/CH_4_ separation. J. Membr. Sci..

[146] Ismail A.F., Li K. (2008). From polymeric precursors to hollow fiber carbon and ceramic membranes. Membr. Sci. Technol..

[147] Reed J.S. (1995). Principles of Ceramics Processing.

[148] Jue M.L., Ma Y., Lively R.P. (2019). Streamlined fabrication of asymmetric carbon molecular sieve hollow fiber membranes. ACS Appl. Polym. Mater..

[149] Ma Y., Jue M.L., Zhang F., Mathias R., Jang H.Y., Lively R.P. (2019). Creation of well-defined “mid-sized” micropores in carbon molecular sieve membranes. Angew. Chem. Int. Ed..

[150] Salinas O., Ma X., Litwiller E., Pinnau I. (2016). Ethylene/ethane permeation, diffusion and gas sorption properties of carbon molecular sieve membranes derived from the prototype ladder polymer of intrinsic microporosity (PIM-1). J. Membr. Sci..

[151] Seong K.H., Song J.S., Koh H.C., Ha S.Y., Han M.H., Cho C.H. (2013). Effect of carbonization conditions on gas permeation of methyl imide based carbon molecular sieve hollow fiber membranes. Membr. J..

[152] Haider S., Lindbråthen A., Lie J.A., Andersen I.C.T., Hägg M.-B. (2018). CO_2_ separation with carbon membranes in high pressure and elevated temperature applications. Sep. Purif. Technol..

[153] Richter H., Voss H., Kaltenborn N., Kämnitz S., Wollbrink A., Feldhoff A., Caro J., Roitsch S., Voigt I. (2017). High-flux carbon molecular sieve membranes for gas separation. Angew. Chem. Int. Ed..

[154] Wijmans J., Hao P. (2015). Influence of the porous support on diffusion in composite membranes. J. Membr. Sci..

[155] Xia Q.-C., Liu M.-L., Cao X.-L., Wang Y., Xing W., Sun S.-P. (2018). Structure design and applications of dual-layer polymeric membranes. J. Membr. Sci..

[156] Xia Q.-C., Wang J., Wang X., Chen B.-Z., Guo J.-L., Jia T.-Z., Sun S.-P. (2017). A hydrophilicity gradient control mechanism for fabricating delamination-free dual-layer membranes. J. Membr. Sci..

[157] Hermans S., Bernstein R., Volodin A., Vankelecom I.F. (2015). Study of synthesis parameters and active layer morphology of interfacially polymerized polyamide-polysulfone membranes. React. Funct. Polym..

[158] Chen H.Z., Xiao Y.C., Chung T.-S. (2011). Multi-layer composite hollow fiber membranes derived from poly(ethylene glycol) (PEG) containing hybrid materials for CO_2_/N_2_ separation. J. Membr. Sci..

[159] Li P., Chen H.Z., Chung T.-S. (2013). The effects of substrate characteristics and pre-wetting agents on PAN–PDMS composite hollow fiber membranes for CO_2_/N_2_ and O_2_/N_2_ separation. J. Membr. Sci..

[160] Liang C.Z., Chung T.-S. (2018). Robust thin film composite PDMS/PAN hollow fiber membranes for water vapor removal from humid air and gases. Sep. Purif. Technol..

[161] Liang C.Z., Chung T.S. (2018). Ultrahigh flux composite hollow fiber membrane via highly crosslinked PDMS for recovery of hydrocarbons: Propane and propene. Macromol. Rapid Commun..

[162] Chen H.Z., Thong Z., Li P., Chung T.-S. (2014). High performance composite hollow fiber membranes for CO_2_/H_2_ and CO_2_/N_2_ separation. Int. J. Hydrog. Energy.

[163] Veríssimo S., Peinemann K.V., Bordado J. (2005). Thin-film composite hollow fiber membranes: An optimized manufacturing method. J. Membr. Sci..

[164] Park C.H., Kwak S.J., Choi J., Lee K., Lee J.H. (2018). Fabrication of a pilot scale module of thin film composite hollow fiber membrane for osmotic pressure-driven processes. J. Appl. Polym. Sci..

[165] Ren J., McCutcheon J.R. (2017). Making thin film composite hollow fiber forward osmosis membranes at the module scale using commercial ultrafiltration membranes. Ind. Eng. Chem. Res..

[166] Sun S.-P., Chung T.-S. (2013). Outer-selective pressure-retarded osmosis hollow fiber membranes from vacuum-assisted interfacial polymerization for osmotic power generation. Environ. Sci. Technol..

[167] Choi O., Peck D.-H., Park C.H. (2021). High-performance nanofiltration of outer-selective thin-film composite hollow-fiber membranes via continuous interfacial polymerization. J. Ind. Eng. Chem..

[168] Khulbe K.C., Matsuura T. (2018). Thin film composite and/or thin film nanocomposite hollow fiber membrane for water treatment, pervaporation, and gas/vapor separation. Polymers.

[169] Wang M., Wang Z., Li S., Zhang C., Wang J., Wang S. (2013). A high performance antioxidative and acid resistant membrane prepared by interfacial polymerization for CO_2_ separation from flue gas. Energy Environ. Sci..

[170] Hu L., Cheng J., Li Y., Liu J., Zhou J., Cen K. (2017). Amino-functionalized surface modification of polyacrylonitrile hollow fiber-supported polydimethylsiloxane membranes. Appl. Surf. Sci..

[171] Zhou F., Tien H.N., Xu W.L., Chen J.T., Liu Q., Hicks E., Fathizadeh M., Li S., Yu M. (2017). Ultrathin graphene oxide-based hollow fiber membranes with brush-like CO_2_-philic agent for highly efficient CO_2_ capture. Nat. Commun..

[172] Zhou F., Tien H.N., Dong Q., Xu W.L., Li H., Li S., Yu M. (2019). Ultrathin, ethylenediamine-functionalized graphene oxide membranes on hollow fibers for CO_2_ capture. J. Membr. Sci..

[173] Yong W.F., Zhang H. (2021). Recent advances in polymer blend membranes for gas separation and pervaporation. Prog. Mater. Sci..

[174] Govardhan B., Chandrasekhar S., Sridhar S. (2017). Purification of surface water using novel hollow fiber membranes prepared from polyetherimide/polyethersulfone blends. J. Environ. Chem. Eng..

[175] Ter Beek O., Pavlenko D., Stamatialis D. (2020). Hollow fiber membranes for long-term hemodialysis based on polyethersulfone-SlipSkin™ polymer blends. J. Membr. Sci..

[176] Wu P., Jiang L.Y., Hu B. (2018). Fabrication of novel PVDF/(PVDF-co-HFP) blend hollow fiber membranes for DCMD. J. Membr. Sci..

[177] Noor N., Koll J., Scharnagl N., Abetz C., Abetz V. (2018). Hollow fiber membranes of blends of polyethersulfone and sulfonated polymers. Membranes.

[178] Sikorska W., Wasyłeczko M., Przytulska M., Wojciechowski C., Rokicki G., Chwojnowski A. (2021). Chemical degradation of PSF-PUR blend hollow fiber membranes-assessment of changes in properties and morphology after hydrolysis. Membranes.

[179] Fosi-Kofal M., Mustafa A., Ismail A., Rezaei-DashtArzhandi M., Matsuura T. (2016). PVDF/CaCO_3_ composite hollow fiber membrane for CO_2_ absorption in gas-liquid membrane contactor. J. Nat. Gas Sci. Eng..

[180] Villalobos L.F., Hilke R., Akhtar F.H., Peinemann K.V. (2018). Fabrication of polybenzimidazole/palladium nanoparticles hollow fiber membranes for hydrogen purification. Adv. Energy Mater..

[181] Jamil A., Oh P.C., Shariff A.M. (2018). Polyetherimide-montmorillonite mixed matrix hollow fibre membranes: Effect of inorganic/organic montmorillonite on CO_2_/CH_4_ separation. Sep. Purif. Technol..

[182] Cao Y., Zhang K., Zhang C., Koros W.J. (2022). Carbon molecular sieve hollow fiber membranes derived from dip-coated precursor hollow fibers comprising nanoparticles. J. Membr. Sci..

[183] Choi O., Kim Y., Jeon J.-D., Kim T.-H. (2021). Preparation of thin film nanocomposite hollow fiber membranes with polydopamine-encapsulated Engelhard titanosilicate-4 for gas separation applications. J. Membr. Sci..

[184] Dong Z., Zhu H., Hang Y., Liu G., Jin W. (2020). Polydimethylsiloxane (PDMS) composite membrane fabricated on the inner surface of a ceramic hollow fiber: From single-channel to multi-channel. Engineering.

[185] Wan C.F., Yang T., Gai W., De Lee Y., Chung T.-S. (2018). Thin-film composite hollow fiber membrane with inorganic salt additives for high mechanical strength and high power density for pressure-retarded osmosis. J. Membr. Sci..

[186] Abdullah N., Rahman M.A., Othman M.H.D., Ismail A., Jaafar J., Abd Aziz A. (2016). Preparation and characterization of self-cleaning alumina hollow fiber membrane using the phase inversion and sintering technique. Ceram. Int..

[187] Lin Y., Xu Y., Loh C.H., Wang R. (2018). Development of robust fluorinated TiO_2_/PVDF composite hollow fiber membrane for CO_2_ capture in gas-liquid membrane contactor. Appl. Surf. Sci..

[188] Liu X.-W., Cao Y., Li Y.-X., Xu Z.-L., Li Z., Wang M., Ma X.-H. (2019). High-performance polyamide/ceramic hollow fiber TFC membranes with TiO_2_ interlayer for pervaporation dehydration of isopropanol solution. J. Membr. Sci..

[189] Zhang H., Guo Y., Zhang X., Hu X., Wang C., Yang Y. (2020). Preparation and characterization of PSF-TiO_2_ hybrid hollow fiber UF membrane by sol-gel method. J. Polym. Res..

[190] Wang Z., Xu J., Pati S., Chen T., Deng Y., Dewangan N., Meng L., Lin J.Y., Kawi S. (2020). High H_2_ permeable SAPO-34 hollow fiber membrane for high temperature propane dehydrogenation application. AIChE J..

[191] Zhang Y., Chen S., Shi R., Du P., Qiu X., Gu X. (2018). Pervaporation dehydration of acetic acid through hollow fiber supported DD3R zeolite membrane. Sep. Purif. Technol..

[192] Muhamad N., Makhtar S.N.N.M., Abdullah N., Pauzi M.Z.M., Mahpoz N.M.A., Othman M.H.D., Jaafar J., Abas K.H., Fadil N.A., Rahman M.A. (2021). Composite zeolite hollow fiber membrane for the removal of nickel using forward osmosis. J. Water Process. Eng..

[193] Amirabedi P., Akbari A., Yegani R. (2019). Fabrication of hydrophobic PP/CH3SiO_2_ composite hollow fiber membrane for membrane contactor application. Sep. Purif. Technol..

[194] Gong H., Pang H., Du M., Chen Z. (2021). Fabrication of a superhydrophobic mixed matrix PVDF-SiO_2_-HDTMS hollow fiber membrane for membrane contact carbon dioxide absorption. Clean. Eng. Technol..

[195] Zhang H., Li B., Sun D., Miao X., Gu Y. (2018). SiO_2_-PDMS-PVDF hollow fiber membrane with high flux for vacuum membrane distillation. Desalination.

[196] Marti A.M., Wickramanayake W., Dahe G., Sekizkardes A., Bank T.L., Hopkinson D.P., Venna S.R. (2017). Continuous flow processing of ZIF-8 membranes on polymeric porous hollow fiber supports for CO_2_ capture. ACS Appl. Mater. Interfaces.

[197] Etxeberria-Benavides M., Johnson T., Cao S., Zornoza B., Coronas J., Sanchez-Lainez J., Sabetghadam A., Liu X., Andres-Garcia E., Kapteijn F. (2020). PBI mixed matrix hollow fiber membrane: Influence of ZIF-8 filler over H_2_/CO_2_ separation performance at high temperature and pressure. Sep. Purif. Technol..

[198] Lin Y., Chen Y., Wang R. (2019). Thin film nanocomposite hollow fiber membranes incorporated with surface functionalized HKUST-1 for highly-efficient reverses osmosis desalination process. J. Membr. Sci..

[199] Liu Q., Li Y., Li Q., Liu G., Liu G., Jin W. (2019). Mixed-matrix hollow fiber composite membranes comprising of PEBA and MOF for pervaporation separation of ethanol/water mixtures. Sep. Purif. Technol..

[200] Zhu J., Meng X., Zhao J., Jin Y., Yang N., Zhang S., Sunarso J., Liu S. (2017). Facile hydrogen/nitrogen separation through graphene oxide membranes supported on YSZ ceramic hollow fibers. J. Membr. Sci..

[201] Qu K., Dai L., Xia Y., Wang Y., Zhang D., Wu Y., Yao Z., Huang K., Guo X., Xu Z. (2021). Self-crosslinked MXene hollow fiber membranes for H_2_/CO_2_ separation. J. Membr. Sci..

[202] Janakiram S., Espejo J.L.M., Høisæter K.K., Lindbråthen A., Ansaloni L., Deng L. (2020). Three-phase hybrid facilitated transport hollow fiber membranes for enhanced CO_2_ separation. Appl. Mater. Today.

[203] Syed Ibrahim G., Isloor A.M., Ismail A., Farnood R. (2020). One-step synthesis of zwitterionic graphene oxide nanohybrid: Application to polysulfone tight ultrafiltration hollow fiber membrane. Sci. Rep..

[204] Wu W., Zhang X., Qin L., Li X., Meng Q., Shen C., Zhang G. (2020). Enhanced MPBR with polyvinylpyrrolidone-graphene oxide/PVDF hollow fiber membrane for efficient ammonia nitrogen wastewater treatment and high-density Chlorella cultivation. Chem. Eng. J..

[205] Xu Y., Lin Y., Lee M., Malde C., Wang R. (2018). Development of low mass-transfer-resistance fluorinated TiO_2_-SiO_2_/PVDF composite hollow fiber membrane used for biogas upgrading in gas-liquid membrane contactor. J. Membr. Sci..

[206] Zhu L., Ji J., Wang S., Xu C., Yang K., Xu M. (2018). Removal of Pb (II) from wastewater using Al_2_O_3_-NaA zeolite composite hollow fiber membranes synthesized from solid waste coal fly ash. Chemosphere.

[207] Wang S., Tian J., Wang Q., Zhao Z., Cui F., Li G. (2019). Low-temperature sintered high-strength CuO doped ceramic hollow fiber membrane: Preparation, characterization and catalytic activity. J. Membr. Sci..

[208] Charcosset C. (2012). Membrane Processes in Biotechnology and Pharmaceutics.

[209] Manni A., Achiou B., Karim A., Harrati A., Sadik C., Ouammou M., Alami Younssi S., El Bouari A. (2020). New low-cost ceramic microfiltration membrane made from natural magnesite for industrial wastewater treatment. J. Environ. Chem. Eng..

[210] Goswami L., Kumar R.V., Pakshirajan K., Pugazhenthi G. (2019). A novel integrated biodegradation-microfiltration system for sustainable wastewater treatment and energy recovery. J. Hazard. Mater..

[211] Carter B., DiMarzo L., Pranata J., Barbano D.M., Drake M. (2021). Efficiency of removal of whey protein from sweet whey using polymeric microfiltration membranes. J. Dairy Sci..

[212] Doulia D.S., Anagnos E.K., Liapis K.S., Klimentzos D.A. (2016). Removal of pesticides from white and red wines by microfiltration. J. Hazard. Mater..

[213] Rouquié C., Dahdouh L., Delalonde M., Wisniewski C. (2019). New prospects for immersed hollow-fiber membranes in fruit juices microfiltration: Case of grapefruit juice. J. Food Eng..

[214] Liu D., Zhu J., Qiu M., He C. (2016). Antifouling performance of poly (lysine methacrylamide)-grafted PVDF microfiltration membrane for solute separation. Sep. Purif. Technol..

[215] Hernández S., Lei S., Rong W., Ormsbee L., Bhattacharyya D. (2016). Functionalization of flat sheet and hollow fiber microfiltration membranes for water applications. ACS Sustain. Chem. Eng..

[216] Zhu L., Chen M., Dong Y., Tang C.Y., Huang A., Li L. (2016). A low-cost mullite-titania composite ceramic hollow fiber microfiltration membrane for highly efficient separation of oil-in-water emulsion. Water Res..

[217] Schopf R., Schmidt F., Linner J., Kulozik U. (2021). Comparative assessment of tubular ceramic, spiral wound, and hollow fiber membrane microfiltration module systems for milk protein fractionation. Foods.

[218] Schopf R., Schmidt F., Kulozik U. (2021). Impact of hollow fiber membrane length on the milk protein fractionation. J. Membr. Sci..

[219] Giacobbo A., Meneguzzi A., Bernardes A.M., de Pinho M.N. (2017). Pressure-driven membrane processes for the recovery of antioxidant compounds from winery effluents. J. Clean. Prod..

[220] Mondal M., De S. (2018). Enrichment of (−) epigallocatechin gallate (EGCG) from aqueous extract of green tea leaves by hollow fiber microfiltration: Modeling of flux decline and identification of optimum operating conditions. Sep. Purif. Technol..

[221] Behroozi A.H., Ataabadi M.R. (2021). Improvement in microfiltration process of oily wastewater: A comprehensive review over two decades. J. Environ. Chem. Eng..

[222] Abd Aziz M.H., Othman M.H.D., Hashim N.A., Adam M.R., Mustafa A. (2019). Fabrication and characterization of mullite ceramic hollow fiber membrane from natural occurring ball clay. Appl. Clay Sci..

[223] Bai Z., Zhang R., Wang S., Gao S., Tian J. (2021). Membrane fouling behaviors of ceramic hollow fiber microfiltration (MF) membranes by typical organic matters. Sep. Purif. Technol..

[224] He Z., Lyu Z., Gu Q., Zhang L., Wang J. (2019). Ceramic-based membranes for water and wastewater treatment. Colloids. Surf. A Physicochem. Eng. Asp..

[225] Abadikhah H., Wang J.-W., Xu X., Agathopoulos S. (2019). SiO_2_ nanoparticles modified Si_3_N_4_ hollow fiber membrane for efficient oily wastewater microfiltration. J. Water Process. Eng..

[226] Hubadillah S.K., Othman M.H.D., Rahman M.A., Ismail A., Jaafar J. (2020). Preparation and characterization of inexpensive kaolin hollow fibre membrane (KHFM) prepared using phase inversion/sintering technique for the efficient separation of real oily wastewater. Arab. J. Chem..

[227] Paul M., Jons S.D. (2016). Chemistry and fabrication of polymeric nanofiltration membranes: A review. Polymer.

[228] Labban O., Liu C., Chong T.H. (2017). Fundamentals of low-pressure nanofiltration: Membrane characterization, modeling, and understanding the multi-ionic interactions in water softening. J. Membr. Sci..

[229] Setiawan L., Shi L., Wang R. (2014). Dual layer composite nanofiltration hollow fiber membranes for low-pressure water softening. Polymer.

[230] Lidén A., Lavonen E., Persson K.M., Larson M. (2016). Integrity breaches in a hollow fiber nanofilter–Effects on natural organic matter and virus-like particle removal. Water Res..

[231] Choi J., Dorji P., Shon H.K., Hong S. (2019). Applications of capacitive deionization: Desalination, softening, selective removal, and energy efficiency. Desalination.

[232] Sengur-Tasdemir R., Urper-Bayram G.M., Turken T., Ates-Genceli E., Tarabara V.V., Koyuncu I. (2021). Hollow fiber nanofiltration membranes for surface water treatment: Performance evaluation at the pilot scale. J. Water Process. Eng..

[233] Conidi C., Castro-Muñoz R., Cassano A. (2020). Membrane-based operations in the fruit juice processing industry: A review. Beverages.

[234] Li H., Zeng X., Shi W., Zhang H., Huang S., Zhou R., Qin X. (2020). Recovery and purification of potato proteins from potato starch wastewater by hollow fiber separation membrane integrated process. Innov. Food Sci. Emerg. Technol..

[235] Paltrinieri L., Remmen K., Müller B., Chu L., Köser J., Wintgens T., Wessling M., de Smet L.C., Sudhölter E.J. (2019). Improved phosphoric acid recovery from sewage sludge ash using layer-by-layer modified membranes. J. Membr. Sci..

[236] Remmen K., Müller B., Köser J., Wessling M., Wintgens T. (2020). Assessment of layer-by-layer modified nanofiltration membrane stability in phosphoric acid. Membranes.

[237] Remmen K., Schäfer R., Hedwig S., Wintgens T., Wessling M., Lenz M. (2019). Layer-by-layer membrane modification allows scandium recovery by nanofiltration. Environ. Sci. Water Res. Technol..

[238] Kazemabad M., Verliefde A., Cornelissen E.R., D’Haese A. (2020). Crown ether containing polyelectrolyte multilayer membranes for lithium recovery. J. Membr. Sci..

[239] Bóna Á., Bakonyi P., Galambos I., Bélafi-Bakó K., Nemestóthy N. (2020). Separation of volatile fatty acids from model anaerobic effluents using various membrane technologies. Membranes.

[240] Monte J., Sá M., Galinha C.F., Costa L., Hoekstra H., Brazinha C., Crespo J.G. (2018). Harvesting of Dunaliella salina by membrane filtration at pilot scale. Sep. Purif. Technol..

[241] Avram A.M., Ahmadiannamini P., Vu A., Qian X., Sengupta A., Wickramasinghe S.R. (2017). Polyelectrolyte multilayer modified nanofiltration membranes for the recovery of ionic liquid from dilute aqueous solutions. J. Appl. Polym. Sci..

[242] Wang Q., Lu T.-D., Yan X.-Y., Zhao L.-L., Yin H., Xiong X.-X., Zhou R., Sun S.-P. (2020). Designing nanofiltration hollow fiber membranes based on dynamic deposition technology. J. Membr. Sci..

[243] He Y., Miao J., Chen S., Zhang R., Zhang L., Tang H., Yang H. (2019). Preparation and characterization of a novel positively charged composite hollow fiber nanofiltration membrane based on chitosan lactate. RSC Adv..

[244] Haghighat N., Vatanpour V. (2020). Fouling decline and retention increase of polyvinyl chloride nanofiltration membranes blended by polypyrrole functionalized multiwalled carbon nanotubes. Mater. Today Commun..

[245] Rohani R., Yusoff I.I. (2019). Towards electrically tunable nanofiltration membranes: Polyaniline-coated polyvinylidene fluoride membranes with tunable permeation properties. Iran. Polym. J..

[246] Ma G., Zhao S., Wang Y., Wang Z., Wang J. (2022). Conjugated polyaniline derivative membranes enable ultrafast nanofiltration and organic-solvent nanofiltration. J. Membr. Sci..

[247] Xia L., Ren J., McCutcheon J.R. (2019). Braid-reinforced thin film composite hollow fiber nanofiltration membranes. J. Membr. Sci..

[248] Wang T., He X., Li Y., Li J. (2018). Novel poly (piperazine-amide)(PA) nanofiltration membrane based poly (m-phenylene isophthalamide)(PMIA) hollow fiber substrate for treatment of dye solutions. Chem. Eng. J..

[249] Tian L., Jiang Y., Li S., Han L., Su B. (2020). Graphene oxide interlayered thin-film nanocomposite hollow fiber nanofiltration membranes with enhanced aqueous electrolyte separation performance. Sep. Purif. Technol..

[250] Liu G., Jin W., Xu N. (2015). Graphene-based membranes. Chem. Soc. Rev..

[251] Liu G., Guo Y., Meng B., Wang Z., Liu G., Jin W. (2022). Two-dimensional MXene hollow fiber membrane for divalent ions exclusion from water. Chin. J. Chem. Eng..

[252] Van der Bruggen B., Daems B., Wilms D., Vandecasteele C. (2001). Mechanisms of retention and flux decline for the nanofiltration of dye baths from the textile industry. Sep. Purif. Technol..

[253] Koyuncu I., Topacik D., Yuksel E. (2004). Reuse of reactive dyehouse wastewater by nanofiltration: Process water quality and economical implications. Sep. Purif. Technol..

[254] Chu Z., Chen K., Xiao C., Ji D., Ling H., Li M., Liu H. (2020). Improving pressure durability and fractionation property via reinforced PES loose nanofiltration hollow fiber membranes for textile wastewater treatment. J. Taiwan Inst. Chem. Eng..

[255] Song C., Tang S., Yue S., Cui Z., Du X., Jiang T., He B., Li J. (2022). Design of microstructure for hollow fiber loose nanofiltration separation layer and its compactness-tailoring mechanism. J. Hazard. Mater..

[256] Shi W., Li T., Li H., Du Q., Zhang H., Qin X. (2022). An attempt to enhance water flux of hollow fiber polyamide composite nanofiltration membrane by the incorporation of hydrophilic and compatible PPTA/PSF microparticles. Sep. Purif. Technol..

[257] Tan Z., Chen S., Peng X., Zhang L., Gao C. (2018). Polyamide membranes with nanoscale Turing structures for water purification. Science.

[258] Marchetti P., Jimenez Solomon M.F., Szekely G., Livingston A.G. (2014). Molecular separation with organic solvent nanofiltration: A critical review. Chem. Rev..

[259] Lim S.K., Goh K., Bae T.-H., Wang R. (2017). Polymer-based membranes for solvent-resistant nanofiltration: A review. Chin. J. Chem. Eng..

[260] Shi G.M., Feng Y., Li B., Tham H.M., Lai J.-Y., Chung T.-S. (2021). Recent progress of organic solvent nanofiltration membranes. Prog. Polym. Sci..

[261] Li Y., Cao B., Li P. (2019). Effects of dope compositions on morphologies and separation performances of PMDA-ODA polyimide hollow fiber membranes in aqueous and organic solvent systems. Appl. Surf. Sci..

[262] Li Y., Xue J., Zhang X., Cao B., Li P. (2019). Formation of macrovoid-free PMDA-MDA polyimide membranes using a gelation/non-solvent-induced phase separation method for organic solvent nanofiltration. Ind. Eng. Chem. Res..

[263] Goh K.S., Chong J.Y., Chen Y., Fang W., Bae T.-H., Wang R. (2020). Thin-film composite hollow fibre membrane for low pressure organic solvent nanofiltration. J. Membr. Sci..

[264] Goh K.S., Chen Y., Chong J.Y., Bae T.H., Wang R. (2021). Thin film composite hollow fibre membrane for pharmaceutical concentration and solvent recovery. J. Membr. Sci..

[265] Tashvigh A.A., Chung T.-S. (2019). Robust polybenzimidazole (PBI) hollow fiber membranes for organic solvent nanofiltration. J. Membr. Sci..

[266] Farahani M.H.D.A., Chung T.-S. (2019). A novel crosslinking technique towards the fabrication of high-flux polybenzimidazole (PBI) membranes for organic solvent nanofiltration (OSN). Sep. Purif. Technol..

[267] Chen D., Yan C., Li X., Liu L., Wu D., Li X. (2019). A highly stable PBI solvent resistant nanofiltration membrane prepared via versatile and simple crosslinking process. Sep. Purif. Technol..

[268] Jiang J.X., Su F., Trewin A., Wood C.D., Campbell N.L., Niu H., Dickinson C., Ganin A.Y., Rosseinsky M.J., Khimyak Y.Z. (2007). Conjugated microporous poly (aryleneethynylene) networks. Angew. Chem. Int. Ed..

[269] Liang B., Wang H., Shi X., Shen B., He X., Ghazi Z.A., Khan N.A., Sin H., Khattak A.M., Li L. (2018). Microporous membranes comprising conjugated polymers with rigid backbones enable ultrafast organic-solvent nanofiltration. Nat. Chem..

[270] He X., Sin H., Liang B., Ghazi Z.A., Khattak A.M., Khan N.A., Alanagh H.R., Li L., Lu X., Tang Z. (2019). Controlling the selectivity of conjugated microporous polymer membrane for efficient organic solvent nanofiltration. Adv. Funct. Mater..

[271] Gong J., Lin R.-B., Chen B. (2018). Conjugated microporous polymers with rigid backbones for organic solvent nanofiltration. Chem.

[272] Farahani M.H.D.A., Hua D., Chung T.-S. (2018). Cross-linked mixed matrix membranes (MMMs) consisting of amine-functionalized multi-walled carbon nanotubes and P84 polyimide for organic solvent nanofiltration (OSN) with enhanced flux. J. Membr. Sci..

[273] Ran J., Zhang P., Chu C., Cui P., Ai X., Pan T., Wu Y., Xu T. (2020). Ultrathin lamellar MoS_2_ membranes for organic solvent nanofiltration. J. Membr. Sci..

[274] Guo B.-Y., Jiang S.-D., Tang M.-J., Li K., Sun S., Chen P.-Y., Zhang S. (2019). MoS_2_ membranes for organic solvent nanofiltration: Stability and structural control. J. Phys. Chem. Lett..

[275] Li S., Li C., Song X., Su B., Mandal B., Prasad B., Gao X., Gao C. (2019). Graphene quantum dots-doped thin film nanocomposite polyimide membranes with enhanced solvent resistance for solvent-resistant nanofiltration. ACS Appl. Mater. Interfaces.

[276] Li S., Li C., Su B., Hu M.Z., Gao X., Gao C. (2019). Amino-functionalized graphene quantum dots (aGQDs)-embedded thin film nanocomposites for solvent resistant nanofiltration (SRNF) membranes based on covalence interactions. J. Membr. Sci..

[277] Li Y., Li J., Soria R.B., Volodine A., Van der Bruggen B. (2020). Aramid nanofiber and modified ZIF-8 constructed porous nanocomposite membrane for organic solvent nanofiltration. J. Membr. Sci..

[278] Karimi A., Khataee A., Safarpour M., Vatanpour V. (2020). Development of mixed matrix ZIF-8/polyvinylidene fluoride membrane with improved performance in solvent resistant nanofiltration. Sep. Purif. Technol..

[279] Dai J., Li S., Liu J., He J., Li J., Wang L., Lei J. (2019). Fabrication and characterization of a defect-free mixed matrix membrane by facile mixing PPSU with ZIF-8 core–shell microspheres for solvent-resistant nanofiltration. J. Membr. Sci..

[280] Wang S., Mahalingam D., Sutisna B., Nunes S.P. (2019). 2D-dual-spacing channel membranes for high performance organic solvent nanofiltration. J. Mater. Chem. A.

[281] Fei F., Cseri L., Szekely G., Blanford C.F. (2018). Robust covalently cross-linked polybenzimidazole/graphene oxide membranes for high-flux organic solvent nanofiltration. ACS Appl. Mater. Interfaces.

[282] Liu M.-L., Wang J., Guo J.-L., Lu T.-D., Cao X.-L., Sun S.-P. (2019). Graphene oxide/cross-linked polyimide (GO/CLPI) composite membranes for organic solvent nanofiltration. Chem. Eng. Res. Des..

[283] Su J., Lv X., Li S., Jiang Y., Liu S., Zhang X., Li H., Su B. (2021). High separation performance thin film composite and thin film nanocomposite hollow fiber membranes via interfacial polymerization for organic solvent nanofiltration. Sep. Purif. Technol..

[284] Abadikhah H., Kalali E.N., Behzadi S., Khan S.A., Xu X., Shabestari M.E., Agathopoulos S. (2019). High flux thin film nanocomposite membrane incorporated with functionalized TiO_2_@ reduced graphene oxide nanohybrids for organic solvent nanofiltration. Chem. Eng. Sci..

[285] Farahani M.H.D.A., Chung T.-S. (2018). Solvent resistant hollow fiber membranes comprising P84 polyimide and amine-functionalized carbon nanotubes with potential applications in pharmaceutical, food, and petrochemical industries. Chem. Eng. J..

[286] Elimelech M., Phillip W.A. (2011). The future of seawater desalination: Energy, technology, and the environment. Science.

[287] Xu J., Feng X., Gao C. (2011). Surface modification of thin-film-composite polyamide membranes for improved reverse osmosis performance. J. Membr. Sci..

[288] Yang T., Wan C.F., Zhang J., Gudipati C., Chung T.-S. (2021). Optimization of interfacial polymerization to fabricate thin-film composite hollow fiber membranes in modules for brackish water reverse osmosis. J. Membr. Sci..

[289] Zhao D.L., Japip S., Zhang Y., Weber M., Maletzko C., Chung T.-S. (2020). Emerging thin-film nanocomposite (TFN) membranes for reverse osmosis: A review. Water Res..

[290] Teow Y.H., Mohammad A.W. (2019). New generation nanomaterials for water desalination: A review. Desalination.

[291] Gai W., Zhang Y., Zhao Q., Chung T.-S. (2021). Highly permeable thin film composite hollow fiber membranes for brackish water desalination by incorporating amino functionalized carbon quantum dots and hypochlorite treatment. J. Membr. Sci..

[292] Teramura K., Iguchi S., Mizuno Y., Shishido T., Tanaka T. (2012). Photocatalytic conversion of CO_2_ in water over layered double hydroxides. Angew. Chem. Int. Ed..

[293] Zhao Q., Zhao D.L., Feng F., Chung T.-S., Chen S.B. (2022). Thin-film nanocomposite reverse osmosis membranes incorporated with citrate-modified layered double hydroxides (LDHs) for brackish water desalination and boron removal. Desalination.

[294] Liu C., Guo Y., Zhou Y., Yang B., Xiao K., Zhao H.-Z. (2022). High-hydrophilic and antifouling reverse osmosis membrane prepared based an unconventional radiation method for pharmaceutical plant effluent treatment. Sep. Purif. Technol..

[295] Chen X., Yip N.Y. (2018). Unlocking high-salinity desalination with cascading osmotically mediated reverse osmosis: Energy and operating pressure analysis. Environ. Sci. Technol..

[296] Peters C.D., Hankins N.P. (2019). Osmotically assisted reverse osmosis (OARO): Five approaches to dewatering saline brines using pressure-driven membrane processes. Desalination.

[297] Al-Najar B., Peters C.D., Albuflasa H., Hankins N.P. (2020). Pressure and osmotically driven membrane processes: A review of the benefits and production of nano-enhanced membranes for desalination. Desalination.

[298] Askari M., Liang C.Z., Choong L.T.S., Chung T.-S. (2021). Optimization of TFC-PES hollow fiber membranes for reverse osmosis (RO) and osmotically assisted reverse osmosis (OARO) applications. J. Membr. Sci..

[299] Liu C., Takagi R., Shintani T., Cheng L., Tung K.L., Matsuyama H. (2020). Organic liquid mixture separation using an aliphatic polyketone-supported polyamide organic solvent reverse osmosis (OSRO) membrane. ACS Appl. Mater. Interfaces.

[300] Liu C., Takagi R., Saeki D., Cheng L., Shintani T., Yasui T., Matsuyama H. (2021). Highly improved organic solvent reverse osmosis (OSRO) membrane for organic liquid mixture separation by simple heat treatment. J. Membr. Sci..

[301] Chau J., Basak P., Sirkar K.K. (2018). Reverse osmosis separation of particular organic solvent mixtures by a perfluorodioxole copolymer membrane. J. Membr. Sci..

[302] Jang H.Y., Johnson J., Ma Y., Mathias R., Bhandari D.A., Lively R.P. (2019). Torlon^®^ hollow fiber membranes for organic solvent reverse osmosis separation of complex aromatic hydrocarbon mixtures. AIChE J..

[303] Ma Y., Zhang F., Yang S., Lively R.P. (2018). Evidence for entropic diffusion selection of xylene isomers in carbon molecular sieve membranes. J. Membr. Sci..

[304] Koh D.-Y., McCool B.A., Deckman H.W., Lively R.P. (2016). Reverse osmosis molecular differentiation of organic liquids using carbon molecular sieve membranes. Science.

[305] Liu C., Dong G., Tsuru T., Matsuyama H. (2021). Organic solvent reverse osmosis membranes for organic liquid mixture separation: A review. J. Membr. Sci..

[306] Liu C., Cheng L., Shintani T., Matsuyama H. (2021). AF2400/polyketone composite organic solvent reverse osmosis membrane for organic liquid separation. J. Membr. Sci..

[307] Gonzales R.R., Kato N., Awaji H., Matsuyama H. (2022). Development of polydimethylsiloxane composite membrane for organic solvent separation. Sep. Purif. Technol..

[308] Rivera M.P., Bruno N.C., Finn M.G., Lively R.P. (2021). Organic solvent reverse osmosis using CuAAC-crosslinked molecularly-mixed composite membranes. J. Membr. Sci..

[309] Koros W.J., Zhang C. (2017). Materials for next-generation molecularly selective synthetic membranes. Nat. Mater..

[310] Jafarinejad S. (2021). Forward osmosis membrane technology for nutrient removal/recovery from wastewater: Recent advances, proposed designs, and future directions. Chemosphere.

[311] Xu W., Chen Q., Ge Q. (2017). Recent advances in forward osmosis (FO) membrane: Chemical modifications on membranes for FO processes. Desalination.

[312] Zheng L., Price W.E., McDonald J., Khan S.J., Fujioka T., Nghiem L.D. (2019). New insights into the relationship between draw solution chemistry and trace organic rejection by forward osmosis. J. Membr. Sci..

[313] Lim S., Park K.H., Akther N., Phuntsho S., Choi J.Y., Shon H.K. (2020). Size-controlled graphene oxide for highly permeable and fouling-resistant outer-selective hollow fiber thin-film composite membranes for forward osmosis. J. Membr. Sci..

[314] Fan X., Liu Y., Quan X. (2019). A novel reduced graphene oxide/carbon nanotube hollow fiber membrane with high forward osmosis performance. Desalination.

[315] Choi P.J., Lim S., Shon H., An A.K. (2022). Incorporation of negatively charged silver nanoparticles in outer-selective hollow fiber forward osmosis (OSHF-FO) membrane for wastewater dewatering. Desalination.

[316] Makhtar S.N.N.M., Muhammad N., Rahman M.A., Abas K.H., Abd Aziz A., Sokri M.N.M., Othman M.H.D., Jaafar J. (2020). Zeolite-A deposited on glass hollow fiber for forward osmosis applications. J. Water Process. Eng..

[317] Fan X., Liu Y., Quan X., Chen S. (2018). Highly permeable thin-film composite forward osmosis membrane based on carbon nanotube hollow fiber scaffold with electrically enhanced fouling resistance. Environ. Sci. Technol..

[318] Ren J., McCutcheon J.R. (2018). A new commercial biomimetic hollow fiber membrane for forward osmosis. Desalination.

[319] Engelhardt S., Sadek A., Duirk S. (2018). Rejection of trace organic water contaminants by an Aquaporin-based biomimetic hollow fiber membrane. Sep. Purif. Technol..

[320] Mahpoz N.M., Makhtar S.N.N.M., Pauzi M.Z.M., Abdullah N., Rahman M.A., Abas K.H., Ismail A.F., Othman M.H.D., Jaafar J. (2020). ZIF-8 membrane supported on alumina hollow fiber with enhanced salt removal by forward osmosis. Desalination.

[321] Lim S., Akther N., Bae T.H., Phuntsho S., Merenda A., Dumée L.F., Shon H.K. (2020). Covalent organic framework incorporated outer-selective hollow fiber thin-film nanocomposite membranes for osmotically driven desalination. Desalination.

[322] Engelhardt S., Vogel J., Duirk S.E., Moore F.B., Barton H.A. (2019). Urea and ammonium rejection by an aquaporin-based hollow fiber membrane. J. Water Process. Eng..

[323] Sanahuja-Embuena V., Lim S., Górecki R., Trzaskus K., Hélix-Nielsen C., Shon H.K. (2022). Enhancing selectivity of novel outer-selective hollow fiber forward osmosis membrane by polymer nanostructures. Chem. Eng. J..

[324] Salamanca M., López-Serna R., Palacio L., Hernández A., Prádanos P., Peña M. (2021). Study of the rejection of contaminants of emerging concern by a biomimetic aquaporin hollow fiber forward osmosis membrane. J. Water Process. Eng..

[325] Firouzjaei M.D., Seyedpour S.F., Aktij S.A., Giagnorio M., Bazrafshan N., Mollahosseini A., Samadi F., Ahmadalipour S., Firouzjaei F.D., Esfahani M.R. (2020). Recent advances in functionalized polymer membranes for biofouling control and mitigation in forward osmosis. J. Membr. Sci..

[326] Rastgar M., Bozorg A., Shakeri A., Sadrzadeh M. (2019). Substantially improved antifouling properties in electro-oxidative graphene laminate forward osmosis membrane. Chem. Eng. Res. Des..

[327] Xu M., Zhao P., Tang C.Y., Yi X., Wang X. (2021). Preparation of electrically enhanced forward osmosis (FO) membrane by two-dimensional MXenes for organic fouling mitigation. Chin. Chem. Lett..

[328] Cruz-Tato P., Rivera-Fuentes N., Flynn M., Nicolau E. (2019). Anti-fouling electroconductive forward osmosis membranes: Electrochemical and chemical properties. ACS Appl. Polym. Mater..

[329] Shakeri A., Salehi H., Rastgar M. (2019). Antifouling electrically conductive membrane for forward osmosis prepared by polyaniline/graphene nanocomposite. J. Water Process. Eng..

[330] Abu-Thabit N.Y. (2016). Chemical oxidative polymerization of polyaniline: A practical approach for preparation of smart conductive textiles. J. Chem. Educ..

[331] Xu X., Zhang H., Yu M., Wang Y., Gao T., Yang F. (2019). Conductive thin film nanocomposite forward osmosis membrane (TFN-FO) blended with carbon nanoparticles for membrane fouling control. Sci. Total Environ..

[332] Law J.Y., Mohammad A.W. (2017). Multiple-solute salts as draw solution for osmotic concentration of succinate feed by forward osmosis. J. Ind. Eng. Chem..

[333] Johnson D.J., Suwaileh W.A., Mohammed A.W., Hilal N. (2018). Osmotic’s potential: An overview of draw solutes for forward osmosis. Desalination.

[334] Li D., Zhang X., Yao J., Simon G.P., Wang H. (2011). Stimuli-responsive polymer hydrogels as a new class of draw agent for forward osmosis desalination. Chem. Commun..

[335] Mohammadifakhr M., De Grooth J., Trzaskus K., Roesink H., Kemperman A. (2021). Single-step synthesis of a polyelectrolyte complex hollow-fiber membrane for forward osmosis. Sep. Purif. Technol..

[336] Huang J.J., Chen S., Liao Y., Chen Y., You X., Wang R. (2021). Performance, fouling and cleaning of a thin film composite hollow fiber membrane during fertiliser-drawn forward osmosis process for micro-polluted water. Environ. Sci. Water Res. Technol..

[337] Almoalimi K., Liu Y.Q. (2022). Enhancing ammonium rejection in forward osmosis for wastewater treatment by minimizing cation exchange. J. Membr. Sci..

[338] Akhtar A., Singh M., Subbiah S., Mohanty K. (2021). Sugarcane juice concentration using a novel aquaporin hollow fiber forward osmosis membrane. Food Bioprod. Process..

[339] Teklu H., Gautam D.K., Subbiah S. (2020). Axial flow hollow fiber forward osmosis module analysis for optimum design and operating conditions in desalination applications. Chem. Eng. Sci..

[340] Altaee A., Braytee A., Millar G.J., Naji O. (2019). Energy efficiency of hollow fibre membrane module in the forward osmosis seawater desalination process. J. Membr. Sci..

[341] Im S.J., Jeong S., Jang A. (2021). Forward osmosis (FO)-reverse osmosis (RO) hybrid process incorporated with hollow fiber FO. npj Clean Water.

[342] Low K.S., Wang Y., Ng D.Y.F., Goh K., Li Y., Wang R. (2020). Understanding the effect of transverse vibration on hollow fiber membranes for submerged forward osmosis processes. J. Membr. Sci..

[343] Li T., Law A.W.-K., Jiang Y., Harijanto A.K., Fane A.G. (2016). Fouling control of submerged hollow fibre membrane bioreactor with transverse vibration. J. Membr. Sci..

[344] Cui Y., Chung T.-S. (2018). Pharmaceutical concentration using organic solvent forward osmosis for solvent recovery. Nat. Commun..

[345] Cui Y., Chung T.-S. (2019). Solvent recovery via organic solvent pressure assisted osmosis. Ind. Eng. Chem. Res..

[346] Liang B., He X., Hou J., Li L., Tang Z. (2019). Membrane separation in organic liquid: Technologies, achievements, and opportunities. Adv. Mater..

[347] Goh K.S., Chen Y., Ng D.Y.F., Chew J.W., Wang R. (2022). Organic solvent forward osmosis membranes for pharmaceutical concentration. J. Membr. Sci..

[348] Seo H., Yoon S., Oh B., Chung Y.G., Koh D.Y. (2021). Shape-selective ultramicroporous carbon membranes for sub-0.1 nm organic liquid separation. Adv. Sci..

[349] Wu X., Ding M., Xu H., Yang W., Zhang K., Tian H., Wang H., Xie Z. (2020). Scalable Ti_3_C_2_T_x_ Mxene interlayered forward osmosis membranes for enhanced water purification and organic solvent recovery. ACS Nano.

[350] Tong Z., Guo H., Liu X., Zhang B. (2020). Organic solvent forward osmosis of graphene oxide-based membranes for enrichment of target products. Ind. Eng. Chem. Res..

[351] Feng X., Huang R.Y. (1997). Liquid separation by membrane pervaporation: A review. Ind. Eng. Chem. Res..

[352] Liu G., Jin W. (2021). Pervaporation membrane materials: Recent trends and perspectives. J. Membr. Sci..

[353] Ong Y.K., Shi G.M., Le N.L., Tang Y.P., Zuo J., Nunes S.P., Chung T.-S. (2016). Recent membrane development for pervaporation processes. Prog. Polym. Sci..

[354] Castro-Muñoz R., Galiano F., Fíla V., Drioli E., Figoli A. (2019). Mixed matrix membranes (MMMs) for ethanol purification through pervaporation: Current state of the art. Rev. Chem. Eng..

[355] Zhang X., Wang M., Ji C.-H., Xu X.-R., Ma X.-H., Xu Z.-L. (2018). Multilayer assembled CS-PSS/ceramic hollow fiber membranes for pervaporation dehydration. Sep. Purif. Technol..

[356] Liu D., Zhang Y., Jiang J., Wang X., Zhang C., Gu X. (2015). High-performance NaA zeolite membranes supported on four-channel ceramic hollow fibers for ethanol dehydration. RSC Adv..

[357] Cao Y., Li Y.-X., Wang M., Xu Z.-L., Wei Y.-M., Shen B.-J., Zhu K.-K. (2019). High-flux NaA zeolite pervaporation membranes dynamically synthesized on the alumina hollow fiber inner-surface in a continuous flow system. J. Membr. Sci..

[358] Wang X., Jiang J., Liu D., Xue Y., Zhang C., Gu X. (2017). Evaluation of hollow fiber T-type zeolite membrane modules for ethanol dehydration. Chin. J. Chem. Eng..

[359] Xu Y.M., Japip S., Chung T.-S. (2020). UiO-66-NH_2_ incorporated dual-layer hollow fibers made by immiscibility induced phase separation (I^2^PS) process for ethanol dehydration via pervaporation. J. Membr. Sci..

[360] Sukitpaneenit P., Chung T.-S. (2014). Fabrication and use of hollow fiber thin film composite membranes for ethanol dehydration. J. Membr. Sci..

[361] Tsai H.-A., Wang T.-Y., Huang S.-H., Hu C.-C., Hung W.-S., Lee K.-R., Lai J.-Y. (2017). The preparation of polyamide/polyacrylonitrile thin film composite hollow fiber membranes for dehydration of ethanol mixtures. Sep. Purif. Technol..

[362] Wang J., Zhang W., Li W., Xing W. (2015). Preparation and characterization of chitosan-poly (vinyl alcohol)/polyvinylidene fluoride hollow fiber composite membranes for pervaporation dehydration of isopropanol. Korean J. Chem. Eng..

[363] Zhang X., Li M.-P., Huang Z.-H., Zhang H., Liu W.-L., Xu X.-R., Ma X.-H., Xu Z.-L. (2020). Fast surface crosslinking ceramic hollow fiber pervaporation composite membrane with outstanding separation performance for isopropanol dehydration. Sep. Purif. Technol..

[364] Zuo J., Hua D., Maricar V., Ong Y.K., Chung T.S. (2018). Dehydration of industrial isopropanol (IPA) waste by pervaporation and vapor permeation membranes. J. Appl. Polym. Sci..

[365] Taymazov D., Zhang H., Li W.-X., Li P.-P., Xie F., Gong X.-Y., Zhang S.-N., Ma X.-H., Xu Z.-L. (2021). Construction of MoS_2_ hybrid membranes on ceramic hollow fibers for efficient dehydration of isopropanol solution via pervaporation. Sep. Purif. Technol..

[366] Hua D., Ong Y.K., Wang P., Chung T.-S. (2014). Thin-film composite tri-bore hollow fiber (TFC TbHF) membranes for isopropanol dehydration by pervaporation. J. Membr. Sci..

[367] Hua D., Chung T.S., Shi G.M., Fang C. (2016). Teflon AF2400/Ultem composite hollow fiber membranes for alcohol dehydration by high-temperature vapor permeation. AIChE J..

[368] Wang X., Chen Y., Zhang C., Gu X., Xu N. (2014). Preparation and characterization of high-flux T-type zeolite membranes supported on YSZ hollow fibers. J. Membr. Sci..

[369] Liu X., Wang C., Wang B., Li K. (2017). Novel organic-dehydration membranes prepared from zirconium metal-organic frameworks. Adv. Funct. Mater..

[370] Badiger H., Shukla S., Kalyani S., Sridhar S. (2014). Thin film composite sodium alginate membranes for dehydration of acetic acid and isobutanol. J. Appl. Polym. Sci..

[371] Raza W., Wang J., Yang J., Tsuru T. (2021). Progress in pervaporation membranes for dehydration of acetic acid. Sep. Purif. Technol..

[372] Jiang J., Peng L., Wang X., Qiu H., Ji M., Gu X. (2019). Effect of Si/Al ratio in the framework on the pervaporation properties of hollow fiber CHA zeolite membranes. Microporous Mesoporous Mater..

[373] Zhang Y., Qiu X., Hong Z., Du P., Song Q., Gu X. (2019). All-silica DD3R zeolite membrane with hydrophilic-functionalized surface for efficient and highly-stable pervaporation dehydration of acetic acid. J. Membr. Sci..

[374] Mangindaan D.W., Shi G.M., Chung T.-S. (2014). Pervaporation dehydration of acetone using P84 co-polyimide flat sheet membranes modified by vapor phase crosslinking. J. Membr. Sci..

[375] Wang Y. (2015). Pervaporation dehydration of ethyl acetate via PBI/PEI hollow fiber membranes. Ind. Eng. Chem. Res..

[376] Prasad N.S., Moulik S., Bohra S., Rani K.Y., Sridhar S. (2016). Solvent resistant chitosan/poly (ether-block-amide) composite membranes for pervaporation of n-methyl-2-pyrrolidone/water mixtures. Carbohydr. Polym..

[377] Han Y.-J., Su W.-C., Lai J.-Y., Liu Y.-L. (2015). Hydrophilically surface-modified and crosslinked polybenzimidazole membranes for pervaporation dehydration on tetrahydrofuran aqueous solutions. J. Membr. Sci..

[378] Zhang W., Ying Y., Ma J., Guo X., Huang H., Liu D., Zhong C. (2017). Mixed matrix membranes incorporated with polydopamine-coated metal-organic framework for dehydration of ethylene glycol by pervaporation. J. Membr. Sci..

[379] Castro-Muñoz R., Ahmad M.Z., Fíla V. (2020). Tuning of nano-based materials for embedding into low-permeability polyimides for a featured gas separation. Front. Chem..

[380] Liu H.-X., Wang N., Zhao C., Ji S., Li J.-R. (2018). Membrane materials in the pervaporation separation of aromatic/aliphatic hydrocarbon mixtures—A review. Chin. J. Chem. Eng..

[381] Wu T., Wang N., Li J., Wang L., Zhang W., Zhang G., Ji S. (2015). Tubular thermal crosslinked-PEBA/ceramic membrane for aromatic/aliphatic pervaporation. J. Membr. Sci..

[382] Arregoitia-Sarabia C., González-Revuelta D., Fallanza M., Ortiz A., Gorri D. (2021). Polyether-block-amide thin-film composite hollow fiber membranes for the recovery of butanol from ABE process by pervaporation. Sep. Purif. Technol..

[383] Mirfendereski S.M., Lin J.Y. (2021). High-performance MFI zeolite hollow fiber membranes synthesized by double-layer seeding with variable temperature secondary growth. J. Membr. Sci..

[384] Xia S., Peng Y., Wang Z. (2016). Microstructure manipulation of MFI-type zeolite membranes on hollow fibers for ethanol-water separation. J. Membr. Sci..

[385] Huang K., Li Q., Liu G., Shen J., Guan K., Jin W. (2015). A ZIF-71 hollow fiber membrane fabricated by contra-diffusion. ACS Appl. Mater. Interfaces.

[386] Li D., Yao J., Sun H., Liu B., van Agtmaal S., Feng C. (2018). Recycling of phenol from aqueous solutions by pervaporation with ZSM-5/PDMS/PVDF hollow fiber composite membrane. Appl. Surf. Sci..

[387] Li J., Labreche Y., Wang N., Ji S., An Q. (2019). PDMS/ZIF-8 coating polymeric hollow fiber substrate for alcohol permselective pervaporation membranes. Chin. J. Chem. Eng..

[388] Li Y., Shen J., Guan K., Liu G., Zhou H., Jin W. (2016). PEBA/ceramic hollow fiber composite membrane for high-efficiency recovery of bio-butanol via pervaporation. J. Membr. Sci..

[389] Dong Z., Liu G., Liu S., Liu Z., Jin W. (2014). High performance ceramic hollow fiber supported PDMS composite pervaporation membrane for bio-butanol recovery. J. Membr. Sci..

[390] Saw E.T., Ang K.L., He W., Dong X., Ramakrishna S. (2019). Molecular sieve ceramic pervaporation membranes in solvent recovery: A comprehensive review. J. Environ. Chem. Eng..

[391] Yang D., Majumdar S., Kovenklioglu S., Sirkar K.K. (1995). Hollow fiber contained liquid membrane pervaporation system for the removal of toxic volatile organics from wastewater. J. Membr. Sci..

[392] Uragami T., Matsuoka Y., Miyata T. (2016). Permeation and separation characteristics in removal of dilute volatile organic compounds from aqueous solutions through copolymer membranes consisted of poly(styrene) and poly(dimethylsiloxane) containing a hydrophobic ionic liquid by pervaporation. J. Membr. Sci..

[393] Kujawa J., Cerneaux S., Kujawski W. (2015). Removal of hazardous volatile organic compounds from water by vacuum pervaporation with hydrophobic ceramic membranes. J. Membr. Sci..

[394] Kujawa J., Cerneaux S., Kujawski W. (2015). Highly hydrophobic ceramic membranes applied to the removal of volatile organic compounds in pervaporation. Chem. Eng. J..

[395] Ji M., Gao X., Wang X., Zhang Y., Jiang J., Gu X. (2018). An ensemble synthesis strategy for fabrication of hollow fiber T-type zeolite membrane modules. J. Membr. Sci..

[396] Chaudhari S., Chang D.W., Cho K.Y., Shon M.Y., Kwon Y.S., Nam S.E., Park Y.I. (2020). Polyvinyl alcohol and graphene oxide blending surface coated alumina hollow fiber (AHF) membrane for pervaporation dehydration of epichlorohydrin (ECH)/isopropanol (IPA)/water ternary feed mixture. J. Taiwan Inst. Chem. Eng..

[397] Goh S.H., Lau H.S., Yong W.F. (2022). Metal-organic frameworks (MOFs)-based mixed matrix membranes (MMMs) for gas separation: A review on advanced materials in harsh environmental applications. Small.

[398] Li Y., Cao C., Chung T.-S., Pramoda K.P. (2004). Fabrication of dual-layer polyethersulfone (PES) hollow fiber membranes with an ultrathin dense-selective layer for gas separation. J. Membr. Sci..

[399] Jiang L., Chung T.-S., Li D.F., Cao C., Kulprathipanja S. (2004). Fabrication of Matrimid/polyethersulfone dual-layer hollow fiber membranes for gas separation. J. Membr. Sci..

[400] Liu Y., Chung T.-S., Wang R., Li D.F., Chng M.L. (2003). Chemical cross-linking modification of polyimide/poly(ether sulfone) dual-layer hollow-fiber membranes for gas separation. Ind. Eng. Chem. Res..

[401] Li Y., Chung T.-S., Xiao Y. (2008). Superior gas separation performance of dual-layer hollow fiber membranes with an ultrathin dense-selective layer. J. Membr. Sci..

[402] Li Y., Chung T.-S. (2010). Silver ionic modification in dual-layer hollow fiber membranes with significant enhancement in CO_2_/CH_4_ and O_2_/N_2_ separation. J. Membr. Sci..

[403] Li H., Choi W., Ingole P.G., Lee H.K., Baek I.H. (2016). Oxygen separation membrane based on facilitated transport using cobalt tetraphenylporphyrin-coated hollow fiber composites. Fuel.

[404] Lau C.H., Low B.T., Shao L., Chung T.-S. (2010). A vapor-phase surface modification method to enhance different types of hollow fiber membranes for industrial scale hydrogen separation. Int. J. Hydrog. Energy.

[405] Ingole P.G., Choi W.K., Baek I.-H., Lee H.K. (2015). Highly selective thin film composite hollow fiber membranes for mixed vapor/gas separation. RSC Adv..

[406] Ingole P.G., Choi W.K., Lee G.B., Lee H.K. (2017). Thin-film-composite hollow-fiber membranes for water vapor separation. Desalination.

[407] Roslan R.A., Lau W.J., Sakthivel D.B., Khademi S., Zulhairun A.K., Goh P.S., Ismail A.F., Chong K.C., Lai S.O. (2018). Separation of CO_2_/CH_4_ and O_2_/N_2_ by polysulfone hollow fiber membranes: Effects of membrane support properties and surface coating materials. J. Polym. Eng..

[408] Qin J.-J., Chung T.-S. (2006). Development of high-performance polysulfone/poly(4-vinylpyridine) composite hollow fibers for CO_2_/CH_4_ separation. Desalination.

[409] Li H.B., Shi W.Y., Li J.C., Zhang Y.F. (2014). Preparation of hollow fiber composite membrane for carbon dioxide/methane separation via orthogonal experimental design. Fibers Polym..

[410] Chong K.C., Lai S.O., Lau W.J., Thiam H.S., Ismail A.F., Roslan R.A. (2018). Preparation, characterization, and performance evaluation of polysulfone hollow fiber membrane with PEBAX or PDMS coating for oxygen enhancement process. Polymers.

[411] Ding X., Cao Y., Zhao H., Wang L., Yuan Q. (2008). Fabrication of high performance Matrimid/polysulfone dual-layer hollow fiber membranes for O_2_/N_2_ separation. J. Membr. Sci..

[412] Ingole P.G., Baig M.I., Choi W.K., Lee H.K. (2016). Synthesis and characterization of polyamide/polyester thin-film nanocomposite membranes achieved by functionalized TiO_2_ nanoparticles for water vapor separation. J. Mater. Chem. A.

[413] Baig M.I., Ingole P.G., Choi W.K., Park S.R., Kang E.C., Lee H.K. (2016). Development of carboxylated TiO_2_ incorporated thin film nanocomposite hollow fiber membranes for flue gas dehydration. J. Membr. Sci..

[414] Ingole P.G., Pawar R.R., Baig M.I., Jeon J.D., Lee H.K. (2017). Thin film nanocomposite (TFN) hollow fiber membranes incorporated with functionalized acid-activated bentonite (ABn-NH) clay: Towards enhancement of water vapor permeance and selectivity. J. Mater. Chem. A.

[415] Ingole P.G., Sohail M., Abou-Elanwar A.M., Irshad Baig M., Jeon J.-D., Choi W.K., Kim H., Lee H.K. (2018). Water vapor separation from flue gas using MOF incorporated thin film nanocomposite hollow fiber membranes. Chem. Eng. J..

[416] Choi O., Karki S., Pawar R.R., Hazarika S., Ingole P.G. (2021). A new perspective of functionalized MWCNT incorporated thin film nanocomposite hollow fiber membranes for the separation of various gases. J. Environ. Chem. Eng..

[417] An X., Ingole P.G., Choi W.K., Lee H.K., Hong S.U., Jeon J.-D. (2017). Enhancement of water vapor separation using ETS-4 incorporated thin film nanocomposite membranes prepared by interfacial polymerization. J. Membr. Sci..

[418] An X., Ingole P.G., Choi W.K., Lee H.K., Hong S.U., Jeon J.-D. (2018). Development of thin film nanocomposite membranes incorporated with sulfated β-cyclodextrin for water vapor/N_2_ mixture gas separation. J. Ind. Eng. Chem..

[419] Abou-Elanwar A.M., Shirke Y.M., Ingole P.G., Choi W.K., Lee H., Hong S.U., Lee H.K., Jeon J.-D. (2018). Nanocomposite hollow fiber membranes with recyclable β-cyclodextrin encapsulated magnetite nanoparticles for water vapor separation. J. Mater. Chem. A.

[420] Sridhar S., Veerapur R.S., Patil M.B., Gudasi K.B., Aminabhavi T.M. (2007). Matrimid polyimide membranes for the separation of carbon dioxide from methane. J. Appl. Polym. Sci..

[421] Dominic T.C., William J.K. (2000). Formation of defect-free polyimide hollow fiber membranes for gas separations. J. Membr. Sci..

[422] Falbo F., Tasselli F., Brunetti A., Drioli E., Barbieri G. (2014). Polyimide hollow fiber membranes for CO_2_ separation from wet gas mixtures. Braz. J. Chem. Eng..

[423] Kosuri M.R., Koros W.J. (2008). Defect-free asymmetric hollow fiber membranes from Torlon^®^, a polyamide-imide polymer, for high-pressure CO_2_ separations. J. Membr. Sci..

[424] Jue M.L., Breedveld V., Lively R.P. (2017). Defect-free PIM-1 hollow fiber membranes. J. Membr. Sci..

[425] Askari M., Yang T., Chung T.-S. (2012). Natural gas purification and olefin/paraffin separation using cross-linkable dual-layer hollow fiber membranes comprising β-Cyclodextrin. J. Membr. Sci..

[426] Peng N., Chung T.-S., Chng M.L., Aw W. (2010). Evolution of ultra-thin dense-selective layer from single-layer to dual-layer hollow fibers using novel Extem^®^ polyetherimide for gas separation. J. Membr. Sci..

[427] Mishra N.K., Patil N., Long C., Yi S.L., Hopkinson D., Grunlan J.C., Wilhite B.A. (2020). Enhancing H_2_-permselectivity of high-flux hollow fiber membrane via in-situ layer-by-layer surface treatment. J. Membr. Sci..

[428] Zhao B., Peng N., Liang C., Yong W.F., Chung T.-S. (2015). Hollow fiber membrane dehumidification device for air conditioning system. Membranes.

[429] Esposito E., Clarizia G., Bernardo P., Jansen J.C., Sedláková Z., Izák P., Curcio S., Cindio B.D., Tasselli F. (2015). Pebax^®^/PAN hollow fiber membranes for CO_2_/CH_4_ separation. Chem. Eng. Process. Process Intensif..

[430] Yong W.F., Ho Y.X., Chung T.-S. (2017). Nanoparticles embedded in amphiphilic membranes for carbon dioxide separation and dehumidification. ChemSusChem.

[431] Hu L., Cheng J., Li Y., Liu J., Zhou J., Cen K. (2018). In-situ grafting to improve polarity of polyacrylonitrile hollow fiber-supported polydimethylsiloxane membranes for CO_2_ separation. J. Colloid Interface Sci..

[432] Singh R.P., Dahe G.J., Dudeck K.W., Berchtold K.A. (2020). Macrovoid-free high performance polybenzimidazole hollow fiber membranes for elevated temperature H_2_/CO_2_ separations. Int. J. Hydrog. Energy.

[433] Wang K.Y., Weber M., Chung T.-S. (2022). Polybenzimidazoles (PBIs) and state-of-the-art PBI hollow fiber membranes for water, organic solvent and gas separations: A review. J. Mater. Chem. A.

[434] Hao L., Zuo J., Chung T.-S. (2014). Formation of defect-free polyetherimide/PIM-1 hollow fiber membranes for gas separation. AIChE J..

[435] Salih A.A.M., Yi C.H., Yang B.L., Chen P. (2013). Interfacially polymerized composite hollow fiber membrane for CO_2_ separation. Adv. Mat. Res..

[436] Dai Z., Deng J., Yu Q., Helberg R.M.L., Janakiram S., Ansaloni L., Deng L. (2019). Fabrication and evaluation of bio-based nanocomposite TFC hollow fiber membranes for enhanced CO_2_ capture. ACS Appl. Mater. Interfaces.

[437] Dai Z., Deng J., Ansaloni L., Janakiram S., Deng L. (2019). Thin-film-composite hollow fiber membranes containing amino acid salts as mobile carriers for CO_2_ separation. J. Membr. Sci..

[438] Dai Y., Ruan X., Bai F., Yu M., Li H., Zhao Z., He G. (2016). High solvent resistance PTFPMS/PEI hollow fiber composite membrane for gas separation. Appl. Surf. Sci..

[439] Kumbharkar S.C., Liu Y., Li K. (2011). High performance polybenzimidazole based asymmetric hollow fibre membranes for H_2_/CO_2_ separation. J. Membr. Sci..

[440] Kim D.Y., Ryu S., Kim H.-J., Ham H.C., Sohn H., Yoon S.P., Han J., Lim T.-H., Kim J.Y., Lee S.W. (2021). Highly selective asymmetric polybenzimidazole-4,4′-(hexafluoroisopropylidene) bis(benzoic acid) hollow fiber membranes for hydrogen separation. Sep. Purif. Technol..

[441] Lee K.R., Hwang S.T. (1992). Separation of propylene and propane by polyimide hollow-fiber membrane module. J. Membr. Sci..

[442] Krol J.J., Boerrigter M., Koops G.H. (2001). Polyimide hollow fiber gas separation membranes: Preparation and the suppression of plasticization in propane/propylene environments. J. Membr. Sci..

[443] Yoshino M., Nakamura S., Kita H., Okamoto K., Tanihara N., Kusuki Y. (2003). Olefin/paraffin separation performance of asymmetric hollow fiber membrane of 6FDA/BPDA–DDBT copolyimide. J. Membr. Sci..

[444] Liu G., Labreche Y., Li N., Liu Y., Zhang C., Miller S.J., Babu V.P., Bhuwania N., Koros W.J. (2019). Simultaneously tuning denseskin and porous substrate of asymmetric hollow fiber membranes for efficient purification of aggressive natural gas. AIChE J..

[445] Kim K., Ingole P.G., Kim J., Lee H. (2013). Separation performance of PEBAX/PEI hollow fiber composite membrane for SO_2_/CO_2_/N_2_ mixed gas. Chem. Eng. J..

[446] Kim K., Hong S., Kim J., Lee H. (2014). Preparation and performance evaluation of composite hollow fiber membrane for SO_2_ separation. AIChE J..

[447] Babu V.P., Kraftschik B.E., Koros W.J. (2018). Crosslinkable TEGMC asymmetric hollow fiber membranes for aggressive sour gas separations. J. Membr. Sci..

[448] Wang L.-Y., Yong W.F., Yu L.E., Chung T.-S. (2017). Design of high efficiency PVDF-PEG hollow fibers for air filtration of ultrafine particles. J. Membr. Sci..

[449] Chung T.-S., Teoh S.K. (1999). The ageing phenomenon of polyethersulfone hollow fiber membranes for gas separation and their characteristics. J. Membr. Sci..

[450] Zhao C., Xue J., Ran F., Sun S. (2013). Modification of polyethersulfone membranes—A review of methods. Prog. Mater. Sci..

[451] Zhang M., Deng L., Xiang D., Cao B., Hosseini S., Li P. (2019). Approaches to suppress CO_2_-induced plasticization of polyimide membranes in gas separation applications. Processes.

[452] Wang D., Li K., Teo W.K. (2000). Highly permeable polyethersulfone hollow fiber gas separation membranes prepared using water as non-solvent additive. J. Membr. Sci..

[453] Adib H., Hassanajili S., Mowla D., Esmaeilzadeh F. (2015). Fabrication of integrally skinned asymmetric membranes based on nanocomposite polyethersulfone by supercritical CO_2_ for gas separation. J. Supercrit. Fluids.

[454] Ismail A.F., Norida R., Rahman W.A.W.A., Matsuura T., Hashemifard S.A. (2011). Preparation and characterization of hyperthin-skinned and high performances asymmetric polyethersulfone membrane for gas separation. Desalination.

[455] Ma C., Koros W.J. (2018). Physical aging of ester-cross-linked hollow fiber membranes for natural gas separations and mitigation thereof. J. Membr. Sci..

[456] Clarizia G., Tasselli F., Bernardo P. (2020). Effect of physical aging on gas transport in asymmetric polyimide hollow fibers prepared by triple-orifice spinneret. Polymers.

[457] Choi S.-H., Jansen J.C., Tasselli F., Barbieri G., Drioli E. (2010). In-line formation of chemically cross-linked P84^®^ co-polyimide hollow fibre membranes for H_2_/CO_2_ separation. Sep. Purif. Technol..

[458] Omole I.C., Miller S.J., Koros W.J. (2008). Increased molecular weight of a crosslinkable polyimide for spinning plasticization resistant hollow fiber membranes. Macromolecules.

[459] Chen C.-C., Miller S.J., Koros W.J. (2012). Characterization of thermally cross-linkable hollow fiber membranes for natural gas separation. Ind. Eng. Chem. Res..

[460] Thür R., Lemmens V., Van Havere D., van Essen M., Nijmeijer K., Vankelecom I.F.J. (2020). Tuning 6FDA-DABA membrane performance for CO_2_ removal by physical densification and decarboxylation cross-linking during simple thermal treatment. J. Membr. Sci..

[461] Liu Y., Liu Z., Kraftschik B.E., Babu V.P., Bhuwania N., Chinn D., Koros W.J. (2021). Natural gas sweetening using TEGMC polyimide hollow fiber membranes. J. Membr. Sci..

[462] Chung T.-S., Ren J., Wang R., Li D., Liu Y., Pramoda K.P., Cao C., Loh W.W. (2003). Development of asymmetric 6FDA-2,6DAT hollow fiber membranes for CO_2_/CH_4_ separation: Part 2. Suppression of plasticization. J. Membr. Sci..

[463] Sheng L., Ren J., Hua K., Li H., Feng Y., Deng M. (2020). The enhancement of mechanical properties of P84 hollow fiber membranes by thermally annealing below and above T_g_. J. Membr. Sci..

[464] Shao L., Low B.T., Chung T.-S., Greenberg A.R. (2009). Polymeric membranes for the hydrogen economy: Contemporary approaches and prospects for the future. J. Membr. Sci..

[465] Masuda T., Nagai K. (2006). Synthesis and permeation properties of substituted polyacetylenes for gas separation and pervaporation. Materials Science of Membranes for Gas and Vapor Separation.

[466] Inoue R., Kanaya T., Hu Y., Masuda T., Nishida K., Yamamuro O. (2014). Relationship between the local dynamics and gas permeability of polyacetylenes containing polymethylated indan/tetrahydronaphtalene moieties. Polymer.

[467] Lee H.J., Kang S.W. (2020). CO_2_ separation with polymer/aniline composite membranes. Polymers.

[468] Gupta Y., Hellgardt K., Wakeman R.J. (2006). Enhanced permeability of polyaniline based nano-membranes for gas separation. J. Membr. Sci..

[469] Zhao J., Wang Z., Wang J., Wang S. (2012). High-performance membranes comprising polyaniline nanoparticles incorporated into polyvinylamine matrix for CO_2_/N_2_ separation. J. Membr. Sci..

[470] Wang Y., Zhang X., Li J., Liu C., Gao Y., Li N., Xie Z. (2019). Enhancing the CO_2_ separation performance of SPEEK membranes by incorporation of polyaniline-decorated halloysite nanotubes. J. Membr. Sci..

[471] Illing G., Hellgardt K., Wakeman R.J., Jungbauer A. (2001). Preparation and characterisation of polyaniline based membranes for gas separation. J. Membr. Sci..

[472] Stolarczyk A., Lapkowski M. (2001). Investigation of gas separation on polyaniline laminar composite membranes. Synth. Met..

[473] Orlov A.V., Kiseleva S.G., Karpacheva G.P., Teplyakov V.V., Syrtsova D.A., Starannikova L.E., Lebedeva T.L. (2003). Structure and gas separation properties of composite films based on polyaniline. J. Appl. Polym. Sci..

[474] Ghalei B., Kinoshita Y., Wakimoto K., Sakurai K., Mathew S., Yue Y., Kusuda H., Imahori H., Sivaniah E. (2017). Surface functionalization of high free-volume polymers as a route to efficient hydrogen separation membranes. J. Mater. Chem. A.

[475] Giel V., Kredatusová J., Trchová M., Brus J., Žitka J., Peter J. (2016). Polyaniline/polybenzimidazole blends: Characterisation of its physico-chemical properties and gas separation behaviour. Eur. Polym. J..

[476] Giel V., Morávková Z., Peter J., Trchová M. (2017). Thermally treated polyaniline/polybenzimidazole blend membranes: Structural changes and gas transport properties. J. Membr. Sci..

[477] Huang N.-Y., Wang C.-C., Chen C.-Y. (2021). Gas-permeation properties of sandwich-like polyaniline/poly(ethylene vinyl acetate) nanocomposite membranes. Chem. Eng. Res. Des..

[478] Kaner R.B., Anderson M.R., Reiss H., Mattes B.R. (1994). Membranes having selective permeability. U.S. Patent.

[479] Rebattet L., Escoubes M., Genies E., Pineri M. (1995). Effect of doping treatment on gas transport properties and on separation factors of polyaniline memebranes. J. Appl. Polym. Sci..

[480] Wang H.-L., Mattes B.R. (1999). Gas transport and sorption in polyaniline thin film. Synth. Met..

[481] Wang H.-L., Mattes B.R. (2004). Permeable Polyaniline Articles for Gas Separation. U.S. Patent.

[482] Parthasarathy R.V., Menon V.P., Martin C.R. (1997). Unusual gas-transport selectivity in a partially oxidized form of the conductive polymer polypyrrole. Chem. Mater..

[483] Martin C.R., Liang W., Menon V., Parthasarathy R., Parthasarathy A. (1993). Electronically conductive polymers as chemically-selective layers for membrane-based separations. Synth. Met..

[484] Andreeva D.V., Pientka Z., Brozová L., Bleha M., Polotskaya G.A., Elyashevich G.K. (2002). Effect of polymerization conditions of pyrrole on formation, structure and properties of high gas separation thin polypyrrole films. Thin Solid Films.

[485] Son W.-I., Hong J.-M., Kim B.-S. (2005). Polypyrrole composite membrane with high permeability prepared by interfacial polymerization. Korean J. Chem. Eng..

[486] Gulsen D., Hacarloglu P., Toppare L., Yilmaz L. (2001). Effect of preparation parameters on the performance of conductive composite gas separation membranes. J. Membr. Sci..

[487] Hacarlioglu P., Toppare L., Yilmaz L. (2003). Polycarbonate–polypyrrole mixed matrix gas separation membranes. J. Membr. Sci..

[488] Wang X., Ding X., Zhao H., Fu J., Xin Q., Zhang Y. (2020). Pebax-based mixed matrix membranes containing hollow polypyrrole nanospheres with mesoporous shells for enhanced gas permeation performance. J. Membr. Sci..

[489] Asghari M., Saadatmandi S., Parnian M.J. (2021). Polypyrrole-aided surface decoration of graphene oxide nanosheets as fillers for poly(ether-b-amid) mixed matrix membranes to enhance CO_2_ capture. Int. J. Energy Res..

[490] Musselman I.H., Li L., Washmon L., Varadarajan D., Riley S.J., Hmyene M., Ferraris J.P., Balkus K.J. (1999). Poly(3-dodecylthiophene) membranes for gas separations. J. Membr. Sci..

[491] Reid B.D., Ebron V.H.M., Musselman I.H., Ferraris J.P., Balkus K.J. (2002). Enhanced gas selectivity in thin film composite membranes of poly(3-(2-acetoxyethyl)thiophene). J. Membr. Sci..

[492] Zhang M., Jing X., Zhao S., Shao P., Zhang Y., Yuan S., Li Y., Gu C., Wang X., Ye Y. (2019). Electropolymerization of molecular-sieving polythiophene membranes for H_2_ separation. Angew. Chem. Int. Ed..

[493] Norris I.D., Fadeev A.G., Pellegrino J., Mattes B.R. (2005). Development of integrally skinned asymmetric polyaniline hollow fibers for membrane applications. Synth. Met..

[494] Mattes B.R. Transport and Other Physicochemical Properties of Polyaniline. Proceedings of the 8th Annual Meeeting of the North American Membrane Society.

[495] Mattes B.R., Wang H.-L., Yang D., Zhua Y.T., Blumenthala W.R., Hundleya M.F. (1997). Formation of conductive polyaniline fibers derived from highly concentrated emeraldine base solutions. Synth. Met..

[496] Mattes B.R., Wang H.-L., Yang D., Rupprecht L. (1999). Electrically conductive polyaniline fibers prepared by dry-wet spinning techniques. Conductive Polymers and Plastics.

[497] Wang H.-L., Romero R.J., Mattes B.R., Zhu Y., Winokur M.J. (2000). Effect of processing conditions on the properties of high molecular weight conductive polyaniline fiber. J. Polym. Sci. B Polym. Phys..

[498] Yang D., Fadeev A., Adams P.N., Mattes B.R. (2001). Controlling Macrovoid Formation in Wet-Spun Polyaniline Fibers. Smart Structures and Materials 2001: Electroactive Polymer Actuators and Devices.

[499] Adams P.N., Bowman D., Brown L., Yang D., Mattes B.R. (2001). Molecular weight dependence of the physical properties of protonated polyaniline films and fibers. Smart Structures and Materials 2001: Electroactive Polymer Actuators and Devices.

[500] Mattes B.R., Adams P.N., Yang D., Brown L.A., Fadeev A.G., Norris I.D. (2013). Spinning, Doping, Dedoping and Redoping Polyaniline Fiber. U.S. Patent.

[501] Benjamin R.M., Wang H.-L. (2000). Method for Preparing Polyaniline Fibers. U.S. Patent.

[502] Hadi A., Karimi-Sabet J., Nikkho S., Dastbaz A. (2021). Fabrication of ZIF-8/polyethersulfone (PES) mixed matrix hollow fiber membranes for O_2_/N_2_ separation. Chem. Pap..

[503] Park S., Jeong H.-K. (2020). Transforming polymer hollow fiber membrane modules to mixed-matrix hollow fiber membrane modules for propylene/propane separation. J. Membr. Sci..

[504] Zhang C., Zhang K., Xu L., Labreche Y., Kraftschik B., Koros W.J. (2014). Highly scalable ZIF-based mixed-matrix hollow fiber membranes for advanced hydrocarbon separations. AIChE J..

[505] Mubashir M., Yeong Y.F., Chew T.L., Lau K.K. (2019). Comparison of post-treatment methods on the performance of hollow fiber membranes containing metal organic framework in gases separation. Ind. Eng. Chem. Res..

[506] Zhu H., Jie X., Wang L., Kang G., Liu D., Cao Y. (2018). Enhanced gas separation performance of mixed matrix hollow fiber membranes containing post-functionalized S-MIL-53. J. Energy Chem..

[507] Li G., Kujawski W., Knozowska K., Kujawa J. (2021). Thin film mixed matrix hollow fiber membrane fabricated by incorporation of amine functionalized metal-organic framework for CO_2_/N_2_ separation. Materials.

[508] Mubashir M., Yeong Y.F., Chew T.L., Lau K.K. (2019). Optimization of spinning parameters on the fabrication of NH_2_-MIL-53(Al)/cellulose acetate (CA) hollow fiber mixed matrix membrane for CO_2_ separation. Sep. Purif. Technol..

[509] Mubashir M., Yin F.Y., Leng C.T., Keong L.K., Jusoh N. (2020). Study on the effect of process parameters on CO_2_/CH_4_ binary gas separation performance over NH_2_-MIL-53(Al)/cellulose acetate hollow fiber mixed matrix membrane. Polym. Test..

[510] Wijiyanti R., Ubaidillah A.N., Gunawan T., Karim Z.A., Ismail A.F., Smart S., Lin R., Widiastuti N. (2019). Polysulfone mixed matrix hollow fiber membranes using zeolite templated carbon as a performance enhancement filler for gas separation. Chem. Eng. Res. Des..

[511] Park H.B., Jung C.H., Lee Y.M., Hill A.J., Pas S.J., Mudie S.T., Van Wagner E., Freeman B.D., Cookson D.J. (2007). Polymers with cavities tuned for fast selective transport of small molecules and ions. Science.

[512] Kim S., Han S.H., Lee Y.M. (2012). Thermally rearranged (TR) polybenzoxazole hollow fiber membranes for CO_2_ capture. J. Membr. Sci..

[513] Woo K.T., Lee J., Dong G., Kim J.S., Do Y.S., Hung W.-S., Lee K.-R., Barbieri G., Drioli E., Lee Y.M. (2015). Fabrication of thermally rearranged (TR) polybenzoxazole hollow fiber membranes with superior CO_2_/N_2_ separation performance. J. Membr. Sci..

[514] Woo K.T., Lee J., Dong G., Kim J.S., Do Y.S., Jo H.J., Lee Y.M. (2016). Thermally rearranged poly(benzoxazole-co-imide) hollow fiber membranes for CO_2_ capture. J. Membr. Sci..

[515] Woo K.T., Dong G., Lee J., Kim J.S., Do Y.S., Lee W.H., Lee H.S., Lee Y.M. (2016). Ternary mixed-gas separation for flue gas CO_2_ capture using high performance thermally rearranged (TR) hollow fiber membranes. J. Membr. Sci..

[516] Lee J., Kim J.S., Kim J.F., Jo H.J., Park H., Seong J.G., Lee Y.M. (2019). Densification-induced hollow fiber membranes using crosslinked thermally rearranged (XTR) polymer for CO_2_ capture. J. Membr. Sci..

[517] Lee J., Kim J.S., Moon S.-y., Park C.Y., Kim J.F., Lee Y.M. (2020). Dimensionally-controlled densification in crosslinked thermally rearranged (XTR) hollow fiber membranes for CO_2_ capture. J. Membr. Sci..

[518] Brunetti A., Cersosimo M., Kim J.S., Dong G., Fontananova E., Lee Y.M., Drioli E., Barbieri G. (2017). Thermally rearranged mixed matrix membranes for CO_2_ separation: An aging study. Int. J. Greenh. Gas Control.

[519] Smith S.J.D., Hou R., Lau C.H., Konstas K., Kitchin M., Dong G., Lee J., Lee W.H., Seong J.G., Lee Y.M. (2019). Highly permeable thermally rearranged mixed matrix membranes (TR-MMM). J. Membr. Sci..

[520] Nocoń-Szmajda K., Wolińska-Grabczyk A., Jankowski A., Szeluga U., Wójtowicz M., Konieczkowska J., Hercog A. (2020). Gas transport properties of mixed matrix membranes based on thermally rearranged poly(hydroxyimide)s filled with inorganic porous particles. Sep. Purif. Technol..

[521] Soto C., Aguilar Lugo C., Rodríguez S., Palacio L., Lozano Á.E., Prádanos P., Hernandez A. (2020). Enhancement of CO_2_/CH_4_ permselectivity via thermal rearrangement of mixed matrix membranes made from an o-hydroxy polyamide with an optimal load of a porous polymer network. Sep. Purif. Technol..

[522] Japip S., Erifin S., Chung T.-S. (2019). Reduced thermal rearrangement temperature via formation of zeolitic imidazolate framework (ZIF)-8-based nanocomposites for hydrogen purification. Sep. Purif. Technol..

[523] Kim J.S., Moon S.J., Wang H.H., Kim S., Lee Y.M. (2019). Mixed matrix membranes with a thermally rearranged polymer and ZIF-8 for hydrogen separation. J. Membr. Sci..

[524] Dong L., Zhang W., Qu Z., Wan C., Yao Z., Xu J., Kang X., Bai Y., Zhang C. (2020). Cardo-type porous organic nanospheres: Tailoring interfacial compatibility in thermally rearranged mixed matrix membranes for improved hydrogen purification. J. Membr. Sci..

[525] Patel A.K., Acharya N.K. (2020). Thermally rearranged (TR) HAB-6FDA nanocomposite membranes for hydrogen separation. Int. J. Hydrog. Energy.

[526] Yang S., Kwon Y.H., Koh D.-Y., Min B., Liu Y., Nair S. (2019). Highly selective SSZ-13 zeolite hollow fiber membranes by ultraviolet activation at near-ambient temperature. ChemNanoMat.

[527] Yang S., Chiang Y., Nair S. (2019). Scalable one-step gel conversion route to high-performance CHA zeolite hollow fiber membranes and modules for CO_2_ separation. Energy Technol..

[528] Sihar A.S., Othman N.H., Alias N.H., Shahruddin M.Z., Asghrar Syed-Hassan S.S., Rahman M.A., Ismail A.F., Wu Z. (2019). Fabrication of lanthanum-based perovskites membranes on porous alumina hollow fibre (AHF) substrates for oxygen enrichment. Ceram. Int..

[529] Lei L., Lindbråthen A., Hillestad M., Sandru M., Favvas E.P., He X. (2019). Screening cellulose spinning parameters for fabrication of novel carbon hollow fiber membranes for gas separation. Ind. Eng. Chem. Res..

[530] Lei L., Lindbråthen A., Zhang X., Favvas E.P., Sandru M., Hillestad M., He X. (2020). Preparation of carbon molecular sieve membranes with remarkable CO_2_/CH_4_ selectivity for high-pressure natural gas sweetening. J. Membr. Sci..

[531] Karousos D.S., Lei L., Lindbråthen A., Sapalidis A.A., Kouvelos E.P., He X., Favvas E.P. (2020). Cellulose-based carbon hollow fiber membranes for high-pressure mixed gas separations of CO_2_/CH_4_ and CO_2_/N_2_. Sep. Purif. Technol..

[532] Yang R., Chen M.Y., Li P. (2022). Carbon molecular sieve hollow fiber composite membrane derived from PMDA-ODA polyimide for gas separation. High Perform. Polym..

[533] Wey M.-Y., Chen H.-H., Lin Y.-T., Tseng H.-H. (2020). Thin carbon hollow fiber membrane with Knudsen diffusion for hydrogen/alkane separation: Effects of hollow fiber module design and gas flow mode. Int. J. Hydrog. Energy.

[534] Kim S.-J., Lee P.S., Chang J.-S., Nam S.-E., Park Y.-I. (2018). Preparation of carbon molecular sieve membranes on low-cost alumina hollow fibers for use in C_3_H_6_/C_3_H_8_ separation. Sep. Purif. Technol..

[535] Rungta M., Wenz G.B., Zhang C., Xu L., Qiu W., Adams J.S., Koros W.J. (2017). Carbon molecular sieve structure development and membrane performance relationships. Carbon.

[536] Bhuwania N., Labreche Y., Achoundong C.S.K., Baltazar J., Burgess S.K., Karwa S., Xu L., Henderson C.L., Williams P.J., Koros W.J. (2014). Engineering substructure morphology of asymmetric carbon molecular sieve hollow fiber membranes. Carbon.

[537] Song J., Feng B., Tan X., Han N., Sunarso J., Liu S. (2019). Oxygen selective perovskite hollow fiber membrane bundles. J. Membr. Sci..

[538] Leo A., Smart S., Liu S., Diniz da Costa J.C. (2011). High performance perovskite hollow fibres for oxygen separation. J. Membr. Sci..

[539] Han N., Shen Z., Zhao X., Chen R., Thakur V.K. (2022). Perovskite oxides for oxygen transport: Chemistry and material horizons. Sci. Total Environ..

[540] Hu Y., An R., Chu Y., Tan X., Sunarso J., Wang S., Liu S. (2018). Perovskite hollow fiber membranes supported in a porous and catalytically active perovskite matrix for air separation. Sep. Purif. Technol..

[541] Wang T., Liu Z., Xu X., Zhu J., Zhang G., Jin W. (2020). Insights into the design of nineteen-channel perovskite hollow fiber membrane and its oxygen transport behaviour. J. Membr. Sci..

[542] Li Y., Li M., Gao J., Lun Y., Li C., Sunarso J., Tan X. (2020). Characterization of La_0.6_Sr_0.4_CoO_3−δ_ oxygen selective hollow fiber made from acetate precursor-derived powder. Ceram. Int..

[543] Buck F., Feldhoff A., Caro J., Schiestel T. (2021). Permeation improvement of LCCF hollow fiber membranes by spinning and sintering optimization. Sep. Purif. Technol..

[544] Buck F., Bunjaku O., Caro J., Schiestel T. (2022). High-flux CO_2_-stable oxygen transport hollow fiber membranes through surface engineering. J. Eur. Ceram. Soc..

[545] Wei Y., Tang J., Zhou L., Li Z., Wang H. (2011). Oxygen permeation through U-shaped K_2_NiF_4_-type oxide hollow-fiber membranes. Ind. Eng. Chem. Res..

[546] Han N., Zhang S., Meng X., Yang N., Meng B., Tan X., Liu S. (2016). Effect of enhanced oxygen reduction activity on oxygen permeation of La_0.6_Sr_0.4_Co_0.2_Fe_0.8_O_3−δ_ membrane decorated by K_2_NiF_4_-type oxide. J. Alloys Compd..

[547] Han N., Zhang S., Meng B., Tan X. (2015). The effect of microstructure and surface decoration with K_2_NiF_4_-type oxide upon the oxygen permeability of perovskite-type La_0.7_Sr_0.3_FeO_3−δ_ hollow fiber membranes. RSC Adv..

[548] Han N., Meng B., Yang N., Sunarso J., Zhu Z., Liu S. (2018). Enhancement of oxygen permeation fluxes of La_0.6_Sr_0.4_CoO_3−δ_ hollow fiber membrane via macrostructure modification and (La_0.5_Sr_0.5_)_2_CoO_4+δ_ decoration. Chem. Eng. Res. Des..

[549] Han N., Chen R., Chang T., Li L., Wang H., Zeng L. (2019). A novel lanthanum strontium cobalt iron composite membrane synthesised through beneficial phase reaction for oxygen separation. Ceram. Int..

[550] Ma T., Han N., Meng B., Yang N., Zhu Z., Liu S. (2019). Enhancing oxygen permeation via the incorporation of silver inside perovskite oxide membranes. Processes.

[551] Gao J., Lun Y., Han N., Tan X., Fan C., Liu S. (2019). Influence of nitric oxide on the oxygen permeation behavior of La_0.6_Sr_0.4_Co_0.2_Fe_0.8_O_3−δ_ perovskite membranes. Sep. Purif. Technol..

[552] Alqaheem Y., Alomair A.A. (2020). Sealing perovskite membranes for long-term oxygen separation from air. Chem. Pap..

[553] Yeo J.Y.J., Li C., Sunarso J. (2021). Modeling and simulation study of oxygen permeation in La_0.8_Ca_0.2_Fe_0.95_O_3−δ_-Ag hollow fiber membrane module. Mater. Today Proc..

[554] Zhang S., Yeo J.Y.J., Li C., Meng X., Yang N., Sunarso J., Liu S. (2022). Oxygen permeation simulation of La_0.8_Ca_0.2_Fe_0.95_O_3−δ_-Ag hollow fiber membrane at different modes and flow configurations. AIChE J..

[555] Song J., Feng B., Chu Y., Tan X., Gao J., Han N., Liu S. (2019). One-step thermal processing to prepare BaCo_0.95−x_Bi_0.05_Zr_x_O_3−δ_ membranes for oxygen separation. Ceram. Int..

[556] Abdel-Karim A., Leaper S., Skuse C., Zaragoza G., Gryta M., Gorgojo P. (2021). Membrane cleaning and pretreatments in membrane distillation—A review. Chem. Eng. J..

[557] Leaper S., Abdel-Karim A., Gad-Allah T.A., Gorgojo P. (2019). Air-gap membrane distillation as a one-step process for textile wastewater treatment. Chem. Eng. J..

[558] Laqbaqbi M., García-Payo M., Khayet M., El Kharraz J., Chaouch M. (2019). Application of direct contact membrane distillation for textile wastewater treatment and fouling study. Sep. Purif. Technol..

[559] Yang G., Zhang J., Peng M., Du E., Wang Y., Shan G., Ling L., Ding H., Gray S., Xie Z. (2021). A mini review on antiwetting studies in membrane distillation for textile wastewater treatment. Processes.

[560] Hubadillah S.K., Othman M.H.D., Matsuura T., Rahman M.A., Jaafar J., Ismail A., Amin S.Z.M. (2018). Green silica-based ceramic hollow fiber membrane for seawater desalination via direct contact membrane distillation. Sep. Purif. Technol..

[561] Gopi G., Arthanareeswaran G., Ismail A. (2019). Perspective of renewable desalination by using membrane distillation. Chem. Eng. Res. Des..

[562] Jiang L., Chen L., Zhu L. (2020). Fouling process of membrane distillation for seawater desalination: An especial focus on the thermal-effect and concentrating-effect during biofouling. Desalination.

[563] Reis B.G., Araújo A.L.B., Amaral M.C.S., Ferraz H.C. (2018). Comparison of Nanofiltration and Direct Contact Membrane Distillation as an alternative for gold mining effluent reclamation. Chem. Eng. Process. Process. Intensif..

[564] Silva M., Reis B., Grossi L., Amaral M. (2020). Improving the energetic efficiency of direct-contact membrane distillation in mining effluent by using the waste-heat-and-water process as the cooling fluid. J. Clean. Prod..

[565] Ghaffour N., Soukane S., Lee J.-G., Kim Y., Alpatova A. (2019). Membrane distillation hybrids for water production and energy efficiency enhancement: A critical review. Appl. Energy.

[566] Wang W., Shi Y., Zhang C., Hong S., Shi L., Chang J., Li R., Jin Y., Ong C., Zhuo S. (2019). Simultaneous production of fresh water and electricity via multistage solar photovoltaic membrane distillation. Nat. Commun..

[567] Li W., Yang Z., Zhang G., Meng Q. (2013). Heat-treated polyacrylonitrile (PAN) hollow fiber structured packings in isopropanol (IPA)/water distillation with improved thermal and chemical stability. Ind. Eng. Chem. Res..

[568] Su P., Zhang X., Li Y., Chen H., Meng Q., Zhang G. (2019). Distillation of alcohol/water solution in hybrid metal–organic framework hollow fibers. AIChE J..

[569] Chang J., Zuo J., Zhang L., O’Brien G.S., Chung T.-S. (2017). Using green solvent, triethyl phosphate (TEP), to fabricate highly porous PVDF hollow fiber membranes for membrane distillation. J. Membr. Sci..

[570] Li H., Liu H., Shi W., Zhang H., Zhou R., Qin X. (2020). Preparation of hydrophobic zeolitic imidazolate framework-71 (ZIF-71)/PVDF hollow fiber composite membrane for membrane distillation through dilute solution coating. Sep. Purif. Technol..

[571] Khayet M., Essalhi M., Qtaishat M., Matsuura T. (2019). Robust surface modified polyetherimide hollow fiber membrane for long-term desalination by membrane distillation. Desalination.

[572] Liu Z., Pan Q., Xiao C. (2019). Preparation and vacuum membrane distillation performance of a silane coupling agent-modified polypropylene hollow fiber membrane. Desalination.

[573] Song L., Huang Q., Huang Y., Bi R., Xiao C. (2019). An electro-thermal braid-reinforced PVDF hollow fiber membrane for vacuum membrane distillation. J. Membr. Sci..

[574] Suga Y., Takagi R., Matsuyama H. (2022). Effect of hollow fiber membrane properties and operating conditions on preventing scale precipitation in seawater desalination with vacuum membrane distillation. Desalination.

[575] Davenport D.M., Gui M., Ormsbee L.R., Bhattacharyya D. (2016). Development of PVDF membrane nanocomposites via various functionalization approaches for environmental applications. Polymers.

[576] Qu D., Wang J., Hou D., Luan Z., Fan B., Zhao C. (2009). Experimental study of arsenic removal by direct contact membrane distillation. J. Hazard. Mater..

[577] Wang J.-W., Li L., Zhang J.-W., Xu X., Chen C.-S. (2016). β-Sialon ceramic hollow fiber membranes with high strength and low thermal conductivity for membrane distillation. J. Eur. Ceram. Soc..

[578] Gabelman A., Hwang S.-T. (1999). Hollow fiber membrane contactors. J. Membr. Sci..

[579] Yang M.C., Cussler E. (1986). Designing hollow-fiber contactors. AIChE J..

[580] Bazhenov S.D., Bildyukevich A.V., Volkov A.V. (2018). Gas-liquid hollow fiber membrane contactors for different applications. Fibers.

[581] Bakeri G., Naeimifard S., Matsuura T., Ismail A. (2015). A porous polyethersulfone hollow fiber membrane in a gas humidification process. RSC Adv..

[582] Huang X., Wang W., Zheng Z., Fan W., Mao C., Shi J., Li L. (2016). Surface monofunctionalized polymethyl pentene hollow fiber membranes by plasma treatment and hemocompatibility modification for membrane oxygenators. Appl. Surf. Sci..

[583] Su J., Wei Y. (2019). Novel tri-bore PVDF hollow fiber membranes for the control of dissolved oxygen in aquaculture water. J. Water Process. Eng..

[584] Merle T., Pronk W., Von Gunten U. (2017). MEMBRO_3_X, a novel combination of a membrane contactor with advanced oxidation (O_3_/H_2_O_2_) for simultaneous micropollutant abatement and bromate minimization. Environ. Sci. Technol. Lett..

[585] Tilahun E., Bayrakdar A., Sahinkaya E., Çalli B. (2017). Performance of polydimethylsiloxane membrane contactor process for selective hydrogen sulfide removal from biogas. Waste Manag..

[586] Zhang N., Pan Z., Zhang L., Zhang Z. (2019). Decarburization characteristics of coalbed methane by membrane separation technology. Fuel.

[587] Abdolahi-Mansoorkhani H., Seddighi S. (2020). CO_2_ capture by modified hollow fiber membrane contactor: Numerical study on membrane structure and membrane wettability. Fuel Process. Technol..

[588] Qazi S., Gómez-Coma L., Albo J., Druon-Bocquet S., Irabien A., Sanchez-Marcano J. (2020). CO_2_ capture in a hollow fiber membrane contactor coupled with ionic liquid: Influence of membrane wetting and process parameters. Sep. Purif. Technol..

[589] Nakhjiri A.T., Heydarinasab A., Bakhtiari O., Mohammadi T. (2018). The effect of membrane pores wettability on CO_2_ removal from CO_2_/CH_4_ gaseous mixture using NaOH, MEA and TEA liquid absorbents in hollow fiber membrane contactor. Chin. J. Chem. Eng..

[590] Zhang Z., Chen F., Rezakazemi M., Zhang W., Lu C., Chang H., Quan X. (2018). Modeling of a CO_2_-piperazine-membrane absorption system. Chem. Eng. Res. Des..

[591] Zhang Z., Yan Y., Zhang L., Chen Y., Ran J., Pu G., Qin C. (2014). Theoretical study on CO_2_ absorption from biogas by membrane contactors: Effect of operating parameters. Ind. Eng. Chem. Res..

[592] Bazhenov S., Lyubimova E. (2016). Gas-liquid membrane contactors for carbon dioxide capture from gaseous streams. Pet. Chem..

[593] Zhao S., Feron P.H., Deng L., Favre E., Chabanon E., Yan S., Hou J., Chen V., Qi H. (2016). Status and progress of membrane contactors in post-combustion carbon capture: A state-of-the-art review of new developments. J. Membr. Sci..

[594] Jin P., Huang C., Shen Y., Zhan X., Hu X., Wang L., Wang L. (2017). Simultaneous separation of H_2_S and CO_2_ from biogas by gas-liquid membrane contactor using single and mixed absorbents. Energy Fuels.

[595] Al-Marzouqi M.H., Marzouk S.A., Abdullatif N. (2017). High pressure removal of acid gases using hollow fiber membrane contactors: Further characterization and long-term operational stability. J. Nat. Gas Sci. Eng..

[596] Tilahun E., Sahinkaya E., Çalli B. (2018). A hybrid membrane gas absorption and bio-oxidation process for the removal of hydrogen sulfide from biogas. Int. Biodeterior. Biodegrad..

[597] Hosseinzadeh A., Hosseinzadeh M., Vatani A., Mohammadi T. (2017). Mathematical modeling for the simultaneous absorption of CO_2_ and SO_2_ using MEA in hollow fiber membrane contactors. Chem. Eng. Process. Process. Intensif..

[598] Zhang Z., Yan Y., Wood D.A., Zhang W., Li L., Zhang L., Van der Bruggen B. (2015). Influence of the membrane module geometry on SO_2_ removal: A numerical study. Ind. Eng. Chem. Res..

[599] Dai Z., Ansaloni L., Deng L. (2016). Precombustion CO_2_ capture in polymeric hollow fiber membrane contactors using ionic liquids: Porous membrane versus nonporous composite membrane. Ind. Eng. Chem. Res..

[600] Bazhenov S., Malakhov A., Bakhtin D., Khotimskiy V., Bondarenko G., Volkov V., Ramdin M., Vlugt T.J., Volkov A. (2018). CO_2_ stripping from ionic liquid at elevated pressures in gas-liquid membrane contactor. Int. J. Greenh. Gas Control.

[601] Dai Z., Noble R.D., Gin D.L., Zhang X., Deng L. (2016). Combination of ionic liquids with membrane technology: A new approach for CO_2_ separation. J. Membr. Sci..

[602] Yu H., Thé J., Tan Z., Feng X. (2016). Modeling SO_2_ absorption into water accompanied with reversible reaction in a hollow fiber membrane contactor. Chem. Eng. Sci..

[603] Li Y., Wang L.A., Zhang Z., Hu X., Cheng Y., Zhong C. (2018). Carbon dioxide absorption from biogas by amino acid salt promoted potassium carbonate solutions in a hollow fiber membrane contactor: A numerical study. Energy Fuels.

[604] Taheri M., Mohebbi A., Hashemipour H., Rashidi A.M. (2016). Simultaneous absorption of carbon dioxide (CO_2_) and hydrogen sulfide (H_2_S) from CO_2_-H_2_S-CH_4_ gas mixture using amine-based nanofluids in a wetted wall column. J. Nat. Gas Sci. Eng..

[605] Rezakazemi M., Darabi M., Soroush E., Mesbah M. (2019). CO_2_ absorption enhancement by water-based nanofluids of CNT and SiO_2_ using hollow-fiber membrane contactor. Sep. Purif. Technol..

[606] Stowe H.M., Hwang G.S. (2017). Molecular insights into the enhanced rate of CO_2_ absorption to produce bicarbonate in aqueous 2-amino-2-methyl-1-propanol. Phys. Chem. Chem. Phys..

[607] Yuan S., Yang Z., Ji X., Chen Y., Sun Y., Lu X. (2017). CO_2_ absorption in mixed aqueous solution of MDEA and cholinium glycinate. Energy Fuels.

[608] Martić I., Maslarević A., Mladenović S., Lukić U., Budimir S. (2015). Water deoxygenation using hollow fiber membrane module with nitrogen as inert gas. Desalin. Water Treat..

[609] Kattan O., Ebbers K., Koolaard A., Vos H., Bargeman G. (2018). Membrane contactors: An alternative for de-aeration of salt solutions?. Sep. Purif. Technol..

[610] Zhang Y., Li K., Wang J., Hou D., Liu H. (2017). Ozone mass transfer behaviors on physical and chemical absorption for hollow fiber membrane contactors. Water Sci. Technol..

[611] Stylianou S., Sklari S., Zamboulis D., Zaspalis V., Zouboulis A. (2015). Development of bubble-less ozonation and membrane filtration process for the treatment of contaminated water. J. Membr. Sci..

[612] Stylianou S.K., Katsoyiannis I.A., Mitrakas M., Zouboulis A.I. (2018). Application of a ceramic membrane contacting process for ozone and peroxone treatment of micropollutant contaminated surface water. J. Hazard. Mater..

[613] Dalane K., Svendsen H.F., Hillestad M., Deng L. (2018). Membrane contactor for subsea natural gas dehydration: Model development and sensitivity study. J. Membr. Sci..

[614] Qu M., Abdelaziz O., Gao Z., Yin H. (2018). Isothermal membrane-based air dehumidification: A comprehensive review. Renew. Sust. Energ. Rev..

[615] Fakharnezhad A., Keshavarz P. (2016). Experimental investigation of gas dehumidification by tri-ethylene glycol in hollow fiber membrane contactors. J. Ind. Eng. Chem..

[616] Bettahalli N.S., Lefers R., Fedoroff N., Leiknes T., Nunes S.P. (2016). Triple-bore hollow fiber membrane contactor for liquid desiccant based air dehumidification. J. Membr. Sci..

[617] Pantelic J., Teitelbaum E., Bozlar M., Kim S., Meggers F. (2018). Development of moisture absorber based on hydrophilic nonporous membrane mass exchanger and alkoxylated siloxane liquid desiccant. Energy Build..

[618] Kirsch V., Roldugin V., Ovcharova A., Bildyukevich A. (2017). Modeling of ethylene absorption from an ethylene-ethane mixture by silver nitrate aqueous solution in a hollow-fiber membrane contactor. Pet. Chem..

[619] Ovcharova A., Vasilevsky V., Borisov I., Bazhenov S., Volkov A., Bildyukevich A., Volkov V. (2017). Polysulfone porous hollow fiber membranes for ethylene-ethane separation in gas-liquid membrane contactor. Sep. Purif. Technol..

[620] Smith K.R., Frumkin H., Balakrishnan K., Butler C.D., Chafe Z.A., Fairlie I., Kinney P., Kjellstrom T., Mauzerall D.L., McKone T.E. (2013). Energy and human health. Annu. Rev. Public Health.

[621] Lee S.-J., Yoon T.-U., Kim A.-R., Kim S.-Y., Cho K.-H., Hwang Y.K., Yeon J.-W., Bae Y.-S. (2016). Adsorptive separation of xenon/krypton mixtures using a zirconium-based metal-organic framework with high hydrothermal and radioactive stabilities. J. Hazard. Mater..

[622] Stern S., Leone S. (1980). Separation of krypton and xenon by selective permeation. AIChE J..

[623] Stern S., Wang S.C. (1980). Permeation cascades for the separation of krypton and xenon from nuclear reactor atmospheres. AIChE J..

[624] Kwon Y.H., Min B., Yang S., Koh D.-Y., Bhave R.R., Nair S. (2018). Ion-exchanged SAPO-34 membranes for krypton-xenon separation: Control of permeation properties and fabrication of hollow fiber membranes. ACS Appl. Mater. Interfaces.

[625] Wang X., Zhou T., Zhang P., Yan W., Li Y., Peng L., Veerman D., Shi M., Gu X., Kapteijn F. (2021). High-silica CHA zeolite membrane with ultra-high selectivity and irradiation stability for krypton/xenon separation. Angew. Chem. Int. Ed..

[626] Kandwal P., Ansari S., Mohapatra P. (2012). A highly efficient supported liquid membrane system for near quantitative recovery of radio-strontium from acidic feeds. Part II: Scale up and mass transfer modeling in hollow fiber configuration. J. Membr. Sci..

[627] Chaudhury S., Bhattacharyya A., Ansari S.A., Goswami A. (2018). A new approach for selective Cs+ separation from simulated nuclear waste solution using electrodriven cation transport through hollow fiber supported liquid membranes. J. Membr. Sci..

[628] Jagasia P., Ansari S.A., Raut D.R., Dhami P.S., Gandhi P.M., Kumar A., Mohapatra P.K. (2016). Hollow fiber supported liquid membrane studies using a process compatible solvent containing calix [4] arene-mono-crown-6 for the recovery of radio-cesium from nuclear waste. Sep. Purif. Technol..

[629] Song J., Chen G., Li X., He T., Jiang B. (2020). Membrane chemical exchange for lithium isotope enrichment (II): Multistage cascade process. Fusion Eng. Des..

[630] Kaleekkal N.J. (2021). Heparin immobilized graphene oxide in polyetherimide membranes for hemodialysis with enhanced hemocompatibility and removal of uremic toxins. J. Membr. Sci..

[631] Verma S.K., Modi A., Singh A.K., Teotia R., Bellare J. (2018). Improved hemodialysis with hemocompatible polyethersulfone hollow fiber membranes: In vitro performance. J. Biomed. Mater. Res. B Appl. Biomater..

[632] Wang J., Liu Z., Qiu M., He C. (2021). Heparin-mimicking semi-interpenetrating composite membrane with multiple excellent performances for promising hemodialysis. J. Membr. Sci..

[633] Wang H., Li J., Liu F., Li T., Zhong Y., Lin H., He J. (2018). Enhanced hemocompatibility of flat and hollow fiber membranes via a heparin free surface crosslinking strategy. React. Funct. Polym..

[634] Narayanaswamy R., Torchilin V.P. (2019). Hydrogels and their applications in targeted drug delivery. Molecules.

[635] Luraghi A., Peri F., Moroni L. (2021). Electrospinning for drug delivery applications: A review. J. Control. Release.

[636] Al-Baadani M.A., Yie K.H.R., Al-Bishari A.M., Alshobi B.A., Zhou Z., Fang K., Dai B., Shen Y., Ma J., Liu J. (2021). Co-electrospinning polycaprolactone/gelatin membrane as a tunable drug delivery system for bone tissue regeneration. Mater. Des..

[637] Yang Y., Li W., Yu D.-G., Wang G., Williams G.R., Zhang Z. (2019). Tunable drug release from nanofibers coated with blank cellulose acetate layers fabricated using tri-axial electrospinning. Carbohydr. Polym..

[638] Wu H., Shi C., Zhu Q., Li Y., Xu Z., Wei C., Chen D., Huang X. (2021). Capillary-driven blood separation and in-situ electrochemical detection based on 3D conductive gradient hollow fiber membrane. Biosens. Bioelectron..

[639] Wu H., Li T., Bao Y., Zhang X., Wang C., Wei C., Xu Z., Tong W., Chen D., Huang X. (2021). MOF-enzyme hybrid nanosystem decorated 3D hollow fiber membranes for in-situ blood separation and biosensing array. Biosens. Bioelectron..

[640] Razali M., Kim J.F., Attfield M., Budd P.M., Drioli E., Lee Y.M., Szekely G. (2015). Sustainable wastewater treatment and recycling in membrane manufacturing. Green Chem..

[641] Kim D., Salazar O.R., Nunes S.P. (2016). Membrane manufacture for peptide separation. Green Chem..

[642] Jung J.T., Wang H.H., Kim J.F., Jeon S.M., Park S.H., Lee W.H., Moon S.J., Drioli E., Lee Y.M. (2021). Microfiber aligned hollow fiber membranes from immiscible polymer solutions by phase inversion. J. Membr. Sci..

[643] Zhao J., Chong J.Y., Shi L., Wang R. (2019). Explorations of combined nonsolvent and thermally induced phase separation (N-TIPS) method for fabricating novel PVDF hollow fiber membranes using mixed diluents. J. Membr. Sci..

[644] Fang C., Jeon S., Rajabzadeh S., Cheng L., Fang L., Matsuyama H. (2018). Tailoring the surface pore size of hollow fiber membranes in the TIPS process. J. Mater. Chem. A.

[645] Lee J., Park B., Kim J., Park S.B. (2015). Effect of PVP, lithium chloride, and glycerol additives on PVDF dual-layer hollow fiber membranes fabricated using simultaneous spinning of TIPS and NIPS. Macromol. Res..

[646] Russo F., Tiecco M., Galiano F., Mancuso R., Gabriele B., Figoli A. (2022). Launching deep eutectic solvents (DESs) and natural deep eutectic solvents (NADESs), in combination with different harmless co-solvents, for the preparation of more sustainable membranes. J. Membr. Sci..

[647] Sanguineti A., Di Nicolo E., Lee Y.M., Drioli E., Zhaoliang C., Hassankiadeh N.T., Lee S.Y. (2020). Process for Manufacturing Fluoropolymer Membranes. U.S. Patent.

[648] Hassankiadeh N.T., Cui Z., Kim J.H., Shin D.W., Lee S.Y., Sanguineti A., Arcella V., Lee Y.M., Drioli E. (2015). Microporous poly (vinylidene fluoride) hollow fiber membranes fabricated with PolarClean as water-soluble green diluent and additives. J. Membr. Sci..

[649] Ursino C., Russo F., Ferrari R., De Santo M., Di Nicolò E., He T., Galiano F., Figoli A. (2020). Polyethersulfone hollow fiber membranes prepared with Polarclean^®^ as a more sustainable solvent. J. Membr. Sci..

[650] Matveev D., Vasilevsky V., Volkov V., Plisko T., Shustikov A., Volkov A., Bildyukevich A. (2022). Fabrication of ultrafiltration membranes from non-toxic solvent dimethylsulfoxide: Benchmarking of commercially available acrylonitrile co-polymers. J. Environ. Chem. Eng..

[651] Lu F., Liu H., Xiao C., Wang X., Chen K., Huang H. (2019). Effect of on-line stretching treatment on the structure and performance of polyvinyl chloride hollow fiber membranes. RSC Adv..

[652] Pelzer M., Vad T., Becker A., Gries T., Markova S., Teplyakov V. (2021). Melt spinning and characterization of hollow fibers from poly (4-methyl-1-pentene). J. Appl. Polym. Sci..

[653] Lv D., Zhu M., Jiang Z., Jiang S., Zhang Q., Xiong R., Huang C. (2018). Green electrospun nanofibers and their application in air filtration. Macromol. Mater. Eng..

[654] Chandavasu C., Xanthos M., Sirkar K., Gogos C. (2003). Fabrication of microporous polymeric membranes by melt processing of immiscible blends. J. Membr. Sci..

[655] Ji D., Xiao C., An S., Chen K., Gao Y., Zhou F., Zhang T. (2020). Completely green and sustainable preparation of PVDF hollow fiber membranes via melt-spinning and stretching method. J. Hazard. Mater..

[656] Ji D., Xiao C., Chen K., Zhou F., Gao Y., Zhang T., Ling H. (2021). Solvent-free green fabrication of PVDF hollow fiber MF membranes with controlled pore structure via melt-spinning and stretching. J. Membr. Sci..

[657] He H.-W., Wang L., Yan X., Zhang L.-H., Yu M., Yu G.-F., Dong R.-H., Xia L.-H., Ramakrishna S., Long Y.-Z. (2016). Solvent-free electrospinning of UV curable polymer microfibers. RSC Adv..

[658] Li X., Zhang Y., Li H., Chen H., Ding Y., Yang W. (2014). Effect of oriented fiber membrane fabricated via needleless melt electrospinning on water filtration efficiency. Desalination.

[659] Markova S.Y., Dukhov A.V., Pelzer M., Shalygin M.G., Vad T., Gries T., Teplyakov V.V. (2021). Designing 3D membrane modules for gas separation based on hollow fibers from poly (4-methyl-1-pentene). Membranes.

[660] Tocci E., Rizzuto C., Macedonio F., Drioli E. (2020). Effect of green solvents in the production of PVDF-specific polymorphs. Ind. Eng. Chem. Res..

[661] Fam W., Mansouri J., Li H., Chen V. (2017). Improving CO_2_ separation performance of thin film composite hollow fiber with Pebax^®^ 1657/ionic liquid gel membranes. J. Membr. Sci..

[662] Gebreyohannes A.Y., Upadhyaya L., Silva L.P., Falca G., Carvalho P.J., Nunes S.P. (2020). Hollow fibers with encapsulated green amino acid-based ionic liquids for dehydration. ACS Sustain. Chem. Eng..

[663] Li M.-S., Zhao Z.-P., Wang M.-X. (2016). Green hydrophilic modification of PE hollow fiber membranes in a module scale via long-distance and dynamic low-temperature H_2_O plasma flow. Appl. Surf. Sci..

[664] Zhang Y., Yang L., Pramoda K.P., Gai W., Zhang S. (2019). Highly permeable and fouling-resistant hollow fiber membranes for reverse osmosis. Chem. Eng. Sci..

[665] Schneiderman D.K., Hillmyer M.A. (2017). 50th anniversary perspective: There is a great Future in sustainable polymers. Macromolecules.

[666] Papageorgiou G.Z. (2018). Thinking green: Sustainable polymers from renewable resources. Polymers.

[667] Zou D., Nunes S.P., Vankelecom I.F.J., Figoli A., Lee Y.M. (2021). Recent advances in polymer membranes employing non-toxic solvents and materials. Green Chem..

[668] Loeb S., Sourirajan S., Gould R.F. (1963). Sea water demineralization by means of an osmotic membrane. Saline Water Conversion—II.

[669] Chen K., Xiao C., Liu H., Ling H., Chu Z., Hu Z. (2019). Design of robust twisted fiber bundle-reinforced cellulose triacetate hollow fiber reverse osmosis membrane with thin separation layer for seawater desalination. J. Membr. Sci..

[670] Kumar M., RaoT S., Isloor A.M., Ibrahim G.P.S., Inamuddin, Ismail N., Ismail A.F., Asiri A.M. (2019). Use of cellulose acetate/polyphenylsulfone derivatives to fabricate ultrafiltration hollow fiber membranes for the removal of arsenic from drinking water. Int. J. Biol. Macromol..

[671] Kumar M., Isloor A.M., Somasekhara Rao T., Ismail A.F., Farnood R., Nambissan P.M.G. (2020). Removal of toxic arsenic from aqueous media using polyphenylsulfone/cellulose acetate hollow fiber membranes containing zirconium oxide. Chem. Eng. J..

[672] Kumar M., Isloor A.M., Todeti S.R., Nagaraja H.S., Ismail A.F., Susanti R. (2021). Effect of binary zinc-magnesium oxides on polyphenylsulfone/cellulose acetate derivatives hollow fiber membranes for the decontamination of arsenic from drinking water. Chem. Eng. J..

[673] Falca G., Musteata V.-E., Behzad A.R., Chisca S., Nunes S.P. (2019). Cellulose hollow fibers for organic resistant nanofiltration. J. Membr. Sci..

[674] Mubashir M., Yeong Y.F., Lau K.K., Chew T.L. (2019). Effect of spinning conditions on the fabrication of cellulose acetate hollow fiber membrane for CO_2_ separation from N_2_ and CH_4_. Polym. Test..

[675] Liu Y., Liu Z., Morisato A., Bhuwania N., Chinn D., Koros W.J. (2020). Natural gas sweetening using a cellulose triacetate hollow fiber membrane illustrating controlled plasticization benefits. J. Membr. Sci..

[676] Aseri N.S., Lau W.J., Goh P.S., Hasbullah H., Othman N.H., Ismail A.F. (2019). Preparation and characterization of polylactic acid-modified polyvinylidene fluoride hollow fiber membranes with enhanced water flux and antifouling resistance. J. Water Process. Eng..

[677] Shen P., Tu K., Yang C.Y., Li J., Du R.X. (2014). Preparation of anti-fouling poly(lactic acid) (PLA) hollow fiber membranes via non-solvent induced phase separation. Adv. Mat. Res..

[678] Xiao C., Xiao C., Chen M., Huang H. (2019). Study on structure and properties of tubular braid-reinforced poly(lactic acid) (PLA) hollow fiber membranes. Desalin. Water Treat..

[679] Zhu L., Liu F., Yu X., Xue L. (2015). Poly(lactic acid) hemodialysis membranes with poly(lactic acid)-*block*-poly(2-hydroxyethyl methacrylate) copolymer as additive: Preparation, characterization, and performance. ACS Appl. Mater. Interfaces.

[680] Rajesh S., Murthy Z.V.P. (2014). Ultrafiltration membranes from waste polyethylene terephthalate and additives: Synthesis and characterization. Quím. Nova.

[681] Kusumocahyo S.P., Ambani S.K., Kusumadewi S., Sutanto H., Widiputri D.I., Kartawiria I.S. (2020). Utilization of used polyethylene terephthalate (PET) bottles for the development of ultrafiltration membrane. J. Environ. Chem. Eng..

[682] Kusumocahyo S.P., Ambani S.K., Marceline S. (2021). Improved permeate flux and rejection of ultrafiltration membranes prepared from polyethylene terephthalate (PET) bottle waste. Sustain. Environ. Res..

[683] Doan H.N., Phong Vo P., Hayashi K., Kinashi K., Sakai W., Tsutsumi N. (2020). Recycled PET as a PDMS-functionalized electrospun fibrous membrane for oil-water separation. J. Environ. Chem. Eng..

[684] Baggio A., Doan H.N., Vo P.P., Kinashi K., Sakai W., Tsutsumi N., Fuse Y., Sangermano M. (2021). Chitosan-functionalized recycled polyethylene terephthalate nanofibrous membrane for sustainable on-demand oil-water separation. Glob. Chall..

[685] Kiani S., Mousavi S.M., Bidaki A. (2021). Preparation of polyethylene terephthalate/xanthan nanofiltration membranes using recycled bottles for removal of diltiazem from aqueous solution. J. Clean. Prod..

[686] Bonfim D.P.F., Cruz F.G.S., Bretas R.E.S., Guerra V.G., Aguiar M.L. (2021). A sustainable recycling alternative: Electrospun PET-membranes for air nanofiltration. Polymers.

[687] Zhuang G.-L., Tseng H.-H., Wey M.-Y. (2016). Feasibility of using waste polystyrene as a membrane material for gas separation. Chem. Eng. Res. Des..

[688] Zhuang G.-L., Wey M.-Y., Tseng H.-H. (2016). A novel technique using reclaimed tire rubber for gas separation membranes. J. Membr. Sci..

[689] Tseng H.-H., Lin Z.-Y., Chen S.-H., Lai W.-H., Wey M.-Y. (2019). Reuse of reclaimed tire rubber for gas-separation membranes prepared by hot-pressing. J. Clean. Prod..

[690] Lai W.-H., Wang D.K., Wey M.-Y., Tseng H.-H. (2020). Recycling waste plastics as hollow fiber substrates to improve the anti-wettability of supported ionic liquid membranes for CO_2_ separation. J. Clean. Prod..

[691] Jamalludin M.R., Harun Z., Othman M.H.D., Hubadillah S.K., Yunos M.Z., Ismail A.F. (2018). Morphology and property study of green ceramic hollow fiber membrane derived from waste sugarcane bagasse ash (WSBA). Ceram. Int..

[692] Tai Z.S., Hubadillah S.K., Othman M.H.D., Dzahir M.I.H.M., Koo K.N., Tendot N.I.S.T.I., Ismail A.F., Rahman M.A., Jaafar J., Aziz M.H.A. (2019). Influence of pre-treatment temperature of palm oil fuel ash on the properties and performance of green ceramic hollow fiber membranes towards oil/water separation application. Sep. Purif. Technol..

